# Supplementary material for: Assessing the quality of tuberculosis care using routine surveillance data: a process evaluation employing the Zero TB Indicator Framework in Mongolia
Source: BMJ Open. 2022 Aug 16;12(8):e061229. doi: 10.1136/bmjopen-2022-061229 (PMC9386240; doi:10.1136/bmjopen-2022-061229)
Supplement: Supplementary data [file bmjopen-2022-061229supp001.pdf]

SUPPLEMENTAL FIGURES AND TABLES

Table S0. Treat cascade indicators and cumulative probability of receiving high-quality TB diagnosis and treatment, stratified by study site.

Indicator	Bayanzurkh	Khan-Uul	Chingeltei	Mandal	p-value
Screened Positive	n=3,245	n=2,620	n=2,160	n=493	
Referred	2,867 (88%)	2,217 (85%)	1,455 (67%)	493 (100%)	<0.001
Evaluated	1,349 (47%)	1,372 (62%)	1,208 (83%)	487 (99%)	<0.001
Diagnosed[1]	595 (44%)	249 (18%)	276 (23%)	42 (9%)	<0.001
Started Treatment[3]	577 (97%)	237 (95%)	271 (98%)	39 (93%)	0.090
Completed Treatment[4]	416 (72%)	202 (85%)	216 (80%)	34 (87%)	<0.001
Cumulatively Diagnosed & Treated	29%	42%	44%	80%	<0.001

Legend: The Zero TB Treat indicators are numbered using superscripts as defined in Table 1. Shaded rows indicate steps whose yield is determined by the quality of care, while non-shaded rows indicate steps whose yield is determined by epidemiology and the sensitivity of screen and diagnostic tools.

Table S1. Predictors of Starting Active TB Treatment (Treatment Cascade)

Characteristic (n=1162)	aOR	95% CI	p-Value
Age (per decade)	0.72	0.66-0.79	<0.001
Male gender	0.73	0.62-0.86	<0.001

Abbreviations: aOR, adjusted odds ratio. 95% CI, 95% Confidence Intervals.

Table S2. Predictors of Completing Active TB Treatment (Treatment Cascade)

Characteristic (n=1124)	aOR	95% CI	p-Value
Age (per decade)	0.76	0.71-0.83	<0.001
Male gender	0.56	0.36-0.88	0.013

Abbreviations: aOR, adjusted odds ratio. 95% CI, 95% Confidence Intervals.

Table S3. Predictors of Completing TB Evaluation (Prevent Cascade)

Characteristic (n=2352)	aOR	95% CI	p-Value
Age of contact (per decade)	0.92	0.86-0.98	0.015
Male gender of contact	0.90	0.73-1.12	0.35
Immediate family member of index patient	0.92	0.71-1.17	0.48
Age of index patient (per decade)	1.01	0.93-1.10	0.81
Male gender of index patient	0.89	0.64-1.24	0.47

Abbreviations: aOR, adjusted odds ratio. 95% CI, 95% Confidence Intervals.

Table S4. Predictors of Being Prescribed and Initiating TPT (Prevent Cascade)

Characteristic (n=86)	aOR	95% CI	p-Value
Age of contact (per year)	0.76	0.55-1.06	0.11
Male gender of contact	0.78	0.22-2.82	0.71
Immediate family member of index patient	0.46	0.10-1.99	0.30
Age of index patient (per decade)	0.60	0.38-0.93	0.022
Male gender of index patient	0.97	0.37-2.57	0.95

Abbreviations: aOR, adjusted odds ratio. 95% CI, 95% Confidence Intervals.

Appendix S1.

Search Strategy

We search all articles in PubMed in all language published between January 1, 2000, and September 3, 2021, using the following strategy:

1. Search terms for tuberculosis

"tuberculosis"[MeSH] OR *Mycobacterium tuberculosis*[tiab] OR TB[tiab]

2. Search terms for cascade analysis

cascade[tiab] OR cascades[tiab] OR continuum[tiab] OR continua[tiab] or Process Assessment, Health Care[MeSH]

3. Search terms for low- and middle-income countries

(Deprived Countries[tw] OR Deprived Population[tw] OR Deprived Populations[tw] OR Developing Countries[tw] OR Developing Country[tw] OR Developing Economies[tw] OR Developing Economy[tw] OR Developing Nation[tw] OR Developing Nations[tw] OR Developing Population[tw] OR Developing Populations[tw] OR Developing World[tw] OR LAMI Countries[tw] OR LAMI Country[tw] OR Less Developed Countries[tw] OR Less Developed Country[tw] OR Less Developed Economies [tw] OR Less Developed Nation[tw] OR Less Developed Nations[tw] OR Less Developed World[tw] OR Lesser Developed Countries[tw] OR Lesser Developed Nations[tw] OR LMIC[tw] OR LMICS[tw] OR Low GDP[tw] OR Low GNP[tw] OR Low Gross Domestic[tw] OR Low Gross National[tw] OR Low Income Countries[tw] OR Low Income Country[tw] OR Low Income Economies [tw] OR Low Income Economy[tw] OR Low Income Nations[tw] OR Low Income Population[tw] OR Low Income Populations[tw] OR Lower GDP[tw] OR lower gross domestic[tw] OR Lower Income Countries[tw] OR Lower Income Country[tw] OR Lower Income Nations[tw] OR Lower Income Population[tw] OR Lower Income Populations[tw] OR Middle Income Countries[tw] OR Middle Income Country[tw] OR Middle Income Economies [tw] OR Middle Income Nation[tw] OR Middle Income Nations[tw] OR Middle Income Population[tw] OR Middle Income Populations[tw] OR Poor Countries[tw] OR Poor Country[tw] OR Poor Economies [tw] OR Poor Economy[tw] OR Poor Nation[tw] OR Poor Nations[tw] OR Poor Population[tw] OR Poor Populations[tw] OR poor world[tw] OR Poorer Countries[tw] OR Poorer Economies [tw] OR Poorer Economy[tw] OR Poorer Nations[tw] OR Poorer Population[tw] OR Poorer Populations[tw] OR Third World[tw] OR Transitional Countries[tw] OR Transitional Country[tw] OR Transitional Economies[tw] OR Transitional Economy[tw] OR Under Developed Countries[tw] OR Under Developed Country[tw] OR under developed nations[tw] OR Under Developed World[tw] OR Under Served Population[tw] OR Under Served Populations[tw] OR Underdeveloped Countries[tw] OR Underdeveloped Country[tw] OR underdeveloped economies[tw] OR underdeveloped nations[tw] OR underdeveloped population[tw] OR Underdeveloped World[tw] OR Underserved Countries[tw] OR Underserved Nations[tw] OR Underserved Population[tw] OR Underserved Populations[tw])

The 23 studies that we identified were:

1. Davis JL, Katamba A, Vasquez J, Crawford E, Sserwanga A, Kakeeto S, Kizito F, Dorsey G, den Boon S, Vittinghoff E, Huang L, Adatu F, Kamya MR, Hopewell PC, Cattamanchi A. Evaluating tuberculosis case detection via real-time monitoring of tuberculosis diagnostic services. Am J Respir Crit Care Med. 2011;184(3):362-7. PMID: 21471088; PMCID: 3175538.

2. Davis JL, Dowdy DW, den Boon S, Walter ND, Katamba A, Cattamanchi A. Test and treat: a new standard for smear-positive tuberculosis. J Acquir Immune Defic Syndr. 2012;61(1):e6-e8. PMID: 22918128; PMCID: 3427531.

3. Marquez C, Davis JL, Katamba A, Haguma P, Ochom E, Ayakaka I, Chamie G, Dorsey G, Kamya MR, Charlebois E, Havlir DV, Cattamanchi A. Assessing the quality of tuberculosis evaluation for children with prolonged cough presenting to routine community health care settings in rural Uganda. PLoS One. 2014;9(8):e105935. PMID: 25170875; PMCID: 4149493.

4. Sun AY, Denkinger CM, Dowdy DW. The impact of novel tests for tuberculosis depends on the diagnostic cascade. Eur Respir J. 2014;44(5):1366-9. PMID: 25186263; PMCID: PMC4254765.

5. Chaisson LH, Katamba A, Haguma P, Ochom E, Ayakaka I, Mugabe F, Miller C, Vittinghoff E, Davis JL, Handley MA, Cattamanchi A. Theory-informed interventions to improve the quality of tuberculosis evaluation at Ugandan health centers: a quasi-experimental study. PLoS ONE. 2015;10(7):e0132573; PMCID: 4501843.

6. Alsdurf H, Hill PC, Matteelli A, Getahun H, Menzies D. The cascade of care in diagnosis and treatment of latent tuberculosis infection: a systematic review and meta-analysis. Lancet Infect Dis. 2016;16(11):1269-78. PMID: 27522233.

7. Daftary A, Jha N, Pai M. Enhancing the role of pharmacists in the cascade of tuberculosis care. Journal of Epidemiology and Global Health. 2016;7(1):1.

8. Subbaraman R, Nathavitharana RR, Satyanarayana S, Pai M, Thomas BE, Chadha VK, Rade K, Swaminathan S, Mayer KH. The Tuberculosis Cascade of Care in India's Public Sector: A Systematic Review and Meta-analysis. PLOS Medicine. 2016;13(10):e1002149.

9. Armstrong-Hough M, Turimumahoro P, Meyer AJ, Ochom E, Babirye D, Ayakaka I, Mark D, Ggita J, Cattamanchi A, Dowdy D, Mugabe F, Fair E, Haberer JE, Katamba A, Davis JL. Drop-out from the tuberculosis contact investigation cascade in a routine public health setting in urban Uganda: A prospective, multi-center study. PLoS One. 2017;12(11):e0187145. PMID: 29108007; PMCID: PMC5673209.

10. Cazabon D, Alsdurf H, Satyanarayana S, Nathavitharana R, Subbaraman R, Daftary A, Pai M. Quality of tuberculosis care in high burden countries: the urgent need to address gaps in the care cascade. Int J Infect Dis. 2017;56:111-6. PMID: 27794468; PMCID: PMC5346036.

11. Hanrahan CF, Van Rie A. A proposed novel framework for monitoring and evaluation of the cascade of HIV-associated TB care at the health facility level. Journal of the International AIDS Society. 2017;20(1):21375.

12. Mwangwa F, Chamie G, Kwarisiima D, Ayieko J, Owaraganise A, Ruel TD, Plenty A, Tram KH, Clark TD, Cohen CR, Bukusi EA, Petersen M, Kamya MR, Charlebois ED, Havlir DV, Marquez C. Gaps in the Child Tuberculosis Care Cascade in 32 Rural Communities in Uganda and Kenya. J Clin Tuberc Other Mycobact Dis. 2017;9:24-9. PMID: 29291251; PMCID: PMC5743212.

13. Naidoo P, Theron G, Rangaka MX, Chihota VN, Vaughan L, Brey ZO, Pillay Y. The South African Tuberculosis Care Cascade: Estimated Losses and Methodological Challenges. J Infect Dis. 2017;216(suppl_7):S702-s13. PMID: 29117342; PMCID: PMC5853316.

14. Yuen C, Becerra M, Codlin A, Creswell J, Ditiu L, Keshavjee S, Khan A, Nicholson T, Page-Shipp L, Sahu S, Wilson M. A best-practice framework of program indicators for monitoring a comprehensive approach to the tuberculosis epidemic. Zero TB Initiative, 2017.

15. Shapiro AE, van Heerden A, Schaafsma TT, Hughes JP, Baeten JM, van Rooyen H, Tumwesigye E, Celum CL, Barnabas RV. Completion of the tuberculosis care cascade in a community-based HIV linkage-to-care study in South Africa and Uganda. J Int AIDS Soc. 2018;21(1). PMID: 29381257; PMCID: PMC5810338.

16. Agins BD, Ikeda DJ, Reid MJA, Goosby E, Pai M, Cattamanchi A. Improving the cascade of global tuberculosis care: moving from the “what” to the “how” of quality improvement. The Lancet Infectious Diseases. 2019;19(12):e437-e43.

17. Arsenault C, Roder-DeWan S, Kruk ME. Measuring and improving the quality of tuberculosis care: A framework and implications from the Lancet Global Health Commission. J Clin Tuberc Other Mycobact Dis. 2019;16:100112. PMID: 31497655; PMCID: PMC6716550.

18. Kim J, Keshavjee S, Atun R. Health systems performance in managing tuberculosis: analysis of tuberculosis care cascades among high-burden and non-high-burden countries. J Glob Health. 2019;9(1):010423. PMID: 31263546; PMCID: PMC6592589 www.icmje.org/coi_disclosure.pdf (available upon request from the corresponding author), and declare no conflicts of interest.

19. Subbaraman R, Nathavitharana RR, Mayer KH, Satyanarayana S, Chadha VK, Arinaminpathy N, Pai M. Constructing care cascades for active tuberculosis: A strategy for program monitoring and identifying gaps in quality of care. PLoS Med. 2019;16(2):e1002754. PMID: 30811385; PMCID: PMC6392267.

20. Der JB, Grint D, Narh CT, Bonsu F, Grant AD. Where are patients missed in the tuberculosis diagnostic cascade? A prospective cohort study in Ghana. PLoS One. 2020;15(3):e0230604. PMID: 32191768; PMCID: PMC7081980.

21. Diaz G, Victoria AM, Meyer AJ, Nino Y, Luna L, Ferro BE, Davis JL. Evaluating the Quality of Tuberculosis Contact Investigation in Cali, Colombia: A Retrospective Cohort Study. Am J Trop Med Hyg. 2021. PMID: 33617470; PMCID: PMC8045602.

22. van de Water B, Meyer T, Wilson M, Young C, Gaunt B, le Roux K. TB prevention cascade at a district hospital in rural Eastern Cape, South Africa. Public Health Action. 2021;11(2):97-100.

23. Yasobant S, Bhavsar P, Kalpana P, Memon F, Trivedi P, Saxena D. Contributing Factors in the Tuberculosis Care Cascade in India: A Systematic Literature Review. Risk Management and Healthcare Policy. 2021;Volume 14:3275-86.

Findings of the systematic review

We searched PubMed in all languages using a systematic search strategy to identify all articles published between January 1, 2000, and September 3, 2021 including compound search terms related to tuberculosis ("tuberculosis"[MeSH] OR *Mycobacterium tuberculosis*[tiab] OR TB[tiab]); cascades, continua of care, or process evaluations in health care (cascade[tiab] OR cascades[tiab] OR continuum[tiab] OR continua[tiab] or Process Assessment, Health Care[MeSH]); and low- and middle-income countries using a complex search strategy (Appendix S1). We screened titles for articles related to TB case-finding, treatment, and/or prevention services, and augmented our search by reviewing citations from retrieved articles to identify additional studies. We identified 22 articles and one monograph from the grey literature, all published between 2011 and 2021 and employing a wide variety of approaches to constructing TB care cascades. Sixteen articles focused on clinic-based TB case finding and treatment, two on TB contact investigation, three on TB preventive therapy, and three on case-finding, prevention, and treatment in combination. Two studies focused on children, while the remaining studies included adults only or adults and children. Eleven studies constructed cascades using primary patient data; four used parameters derived from systematic reviews; three studies used focused literature reviews to parameterize mathematical models; three mentioned the cascade of care as a concept in a review or editorial but did not construct their own cascades; and two proposed integrated frameworks for assessing the quality of case-finding, treatment, and prevention, including one focused on persons living with HIV. Two of the studies constructed cascades by integrating two of these approaches. Only two studies employed cascades as a quality improvement strategy, with both providing real-time performance feedback. Among the 11 studies that constructed cascades from patient data, only two did so using routine surveillance data, and both of these relied on aggregated

national data rather than on individual patient data. The remaining nine studies used subnational data collected by research staff using research databases. We did not identify any studies that collected individual-patient data from routine public health databases.

Appendix S2. Rationale for Zero TB Indicator targets provided in Table 1.

Indicator* (Proportion)	Target/ Source	Rationale
**SEARCH CASCADE**		
1. (Screening) Coverage	100%[1]	WHO guidelines state that all patients at risk for TB should be screened.
2. Positive TB symptom screen or chest radiography	25-50%[1]	Systematic reviews undertaken to inform the WHO guidelines examined the WHO 4-symptom screen in the general population and found a positivity rate for symptom screening of approximately 36%, with the 95% Confidence Interval (CI) ranging from 25 to 48%. Concurrent reviews of chest radiography found a lower positivity rate of approximately 11%, with the 95% CI ranging from 8 to 15%. Since symptom screening is used in concert with chest radiography, we have based the target proportion on symptom screening. Substantially higher or lower proportions may suggest the need for quality assurance procedures to ensure that screeners are asking the questions appropriately.
3. Diagnostic evaluation	100%[1]	WHO guidelines state that all patients who are symptom-positive should be evaluated for TB.
4. (Active) TB Diagnosis	10-20%[2]	The Stop TB Partnership sputum smear microscopy guidelines state that the typical smear positivity rate is 10-20%.† Higher or lower proportions suggest the need for quality assurance procedures to ensure that screening and testing are being carried out appropriately.
5. Linkage to TB Treatment	100%[3]	WHO guidelines state that all patients are diagnosed with active TB should be linked to TB treatment.
**TREAT CASCADE**		
1. (Active) TB Diagnosis	10-20%[2]	The Stop TB Partnership sputum smear microscopy guidelines state that the typical smear positivity rate is 10-20%.† Higher or lower proportions suggest the need for quality assurance procedures to ensure that screening and testing are being carried out appropriately.
2. Bacteriologic Confirmation	70-90%[4,5]	WHO-commissioned systematic reviews cited in the WHO guidelines suggest that microbiologic tests detect just 70-90% of TB cases relative to a reference standard that includes clinically diagnosed cases.
3. Linkage to TB Treatment	100%[3]	WHO guidelines state that all patients who are diagnosed with active TB should be linked to TB treatment.
4. Treatment Success	≥90%[6]	WHO guidelines state that all patients at risk for TB should be screened.
5. TB-free Survival	≥95%[7]	A high-quality systematic review suggests that ≥95% of patients with drug-susceptible TB can be cured with standard 6-month drug regimens.

Appendix S2 continued. Rationale for Zero TB Indicator targets provided in Table 1.

**PREVENT CASCADE**		
TB Screening	100%[8]	WHO guidelines state that all close contacts of TB patients should be screened for active TB.
6. TB Evaluation	≥90%[6]	The END TB Strategy has set a target TB evaluation rate of ≥90% of symptomatic close contacts.
7. (Active) TB Diagnosis	10-20%[2]	Stop TB Partnership sputum microscopy guidelines state that the typical positivity rate is 10-20%.† Higher or lower proportions suggest the need for quality assurance procedures to ensure that screening and testing are being carried out appropriately.
TPT Eligibility	95%[9]	
8. (TPT) Prescription	≥90%[6]	The END TB Strategy has set a target prescription rate for TB preventive therapy of ≥90% of eligible individuals.
9. (TPT) Uptake	100%[6]	The END TB Strategy has set a target uptake rate for TB preventive therapy of ≥90% of eligible individuals
10. (TPT) Completion	≥80%[9]	WHO guidelines state that TPT regimen is considered complete when ≥80% doses have been completed.
11. TB-free survival	100%[9]	Systematic reviews cited within WHO guidelines had a very low TB incidence among those completing TB preventive therapy.

Abbreviations: TB, tuberculosis.
Legend: *Indicator numbers as proposed by the Zero TB Indicator Framework[10]. †The target proportion might be adjusted if a different diagnostic testing modality is in use, or based on robust empirical data.

Appendix S3. Derivation of the variance and confidence interval for estimated cumulative probability of being diagnosed & treated

Let p_1 be the probability of being referred among subjects who were screened positive, p_2 be the probability of being evaluated among subjects who were referred, p_3 be the probability of starting treatment among subjects who were diagnosed TB positive, and p_4 be the probability of complete treatment among subjects who started treatment. Then the cumulative probability of being properly diagnosed and treated for TB given a positive TB diagnosis $p = p_1 p_2\ p_3 p_4$.

Denote n the number of subjects who were screened positive, n_e the number of subjects who were evaluated, n_d the number of subjects who were diagnosed TB positive, and n_c the number of subjects who completed the treatment. The estimated p is

$$\hat{p} = \frac{n_e}{n}\frac{n_c}{n_d},$$

where $\frac{n_e}{n}$ is an estimator of $p_1 p_2$ and $\frac{n_c}{n_d}$ is an estimator of $p_3 p_4$.

We derive the variance of $\log(\hat{p}) = \log\left(\frac{n_e}{n}\right) + \log\left(\frac{n_c}{n_d}\right)$. Suppose the two probabilities $\frac{n_e}{n}$ and $\frac{n_c}{n_d}$ are independent. This is reasonable since the evaluation process and completion of treatment are different.

$$\widehat{Var}[\log(\hat{p})] = \widehat{Var}\left[\log\left(\frac{n_e}{n}\right)\right] + \widehat{Var}\left[\log\left(\frac{n_c}{n_d}\right)\right] = \left(\frac{n_e}{n}\right)^{-2}\widehat{Var}\left(\frac{n_e}{n}\right) + \left(\frac{n_c}{n_d}\right)^{-2}\widehat{Var}\left(\frac{n_c}{n_d}\right)$$
$$= \frac{n - n_e}{n n_e} + \frac{n_d - n_c}{n_d n_c}.$$

The first equality is due to independence of probabilities, the second is due to the delta method, and the last one is simple algebra by substituting the variance of probabilities. The variance of $\hat{p}$ is then by the delta method again,

$$\widehat{Var}[\hat{p}] = \hat{p}^2 * \widehat{Var}[\log(\hat{p})],$$

and the 95% CI is

$$\left(\hat{p} * \exp\left(-1.96 * \widehat{Var}[\log(\hat{p})]\right),\quad \hat{p} * \exp\left(1.96 * \widehat{Var}[\log(\hat{p})]\right)\right).$$

SUPPLEMENTAL REFERENCES

1. World Health Organization. WHO operational handbook on tuberculosis. Module 2: Screening. Systematic screening for tuberculosis disease. Geneva: World Health Organization; 2021.
2. Stop TB Partnership. Laboratory diagnosis of tuberculosis by sputum microscopy. In: Global Laboratory Initiative, editor. Adelaide, Australia: SA Pathology; 2013.
3. World Health Organization. WHO consolidated guidelines on tuberculosis: module 4: treatment: drug-resistant tuberculosis treatment: World Health Organization; 2020.
4. World Health Organization. Policy statement: The use of liquid medium for culture and DST in low- and middle-income country settings. Geneva: World Health Organization; 2007.
5. World Health Organization. WHO Meeting Report of a Technical Expert Consultation: Non-inferiority analysis of Xpert MTB/RIF Ultra compared to Xpert MTB/RIF Geneva: World Health Organization; 2017.
6. World Health Organization. The End TB Strategy. Geneva: World Health Organization; 2015.
7. Imperial MZ, Nahid P, Phillips PPJ, et al. A patient-level pooled analysis of treatment-shortening regimens for drug-susceptible pulmonary tuberculosis. *Nat Med* 2018; **24**(11): 1708-15.
8. World Health Organization. Recommendations for investigating contacts of persons with infectious tuberculosis in low- and middle-income countries. Geneva: World Health Organization; 2012.
9. World Health Organization. Latent tuberculosis infection: Updated and consolidated guidelines for programmatic management. Geneva: World Health Organization; 2018.
10. Yuen C, Becerra M, Codlin A, et al. A best-practice framework of program indicators for monitoring a comprehensive approach to the tuberculosis epidemic: Zero TB Initiative, 2017.
