# Supplementary material for: Assessing the quality of tuberculosis care using routine surveillance data: a process evaluation employing the Zero TB Indicator Framework in Mongolia
Source: BMJ Open. 2022 Aug 16;12(8):e061229. doi: 10.1136/bmjopen-2022-061229 (PMC9386240; doi:10.1136/bmjopen-2022-061229)
Supplement: Author's manuscript [file bmjopen-2022-061229.draft_revisions.pdf]

BMJ Open

BMJ Open is committed to open peer review. As part of this commitment we make the peer review history of every article we publish publicly available.

When an article is published we post the peer reviewers' comments and the authors' responses online. We also post the versions of the paper that were used during peer review. These are the versions that the peer review comments apply to.

The versions of the paper that follow are the versions that were submitted during the peer review process. They are not the versions of record or the final published versions. They should not be cited or distributed as the published version of this manuscript.

BMJ Open is an open access journal and the full, final, typeset and author-corrected version of record of the manuscript is available on our site with no access controls, subscription charges or pay-per-view fees (http://bmjopen.bmj.com).

If you have any questions on BMJ Open's open peer review process please email info.bmjopen@bmj.com

BMJ Open

Assessing the Quality of Tuberculosis Care Using Routine Surveillance Data: A process evaluation employing the Zero TB Indicator Framework in Mongolia

Journal:	*BMJ Open*
Manuscript ID	bmjopen-2022-061229
Article Type:	Original research
Date Submitted by the Author:	09-Feb-2022

Complete List of Authors:	Saranjav, Ariunzaya; Mongolian Health Initiative, Zero TB Mongolia<br>Parisi, Christina; Yale University School of Public Health<br>Zhou, Xin; Yale University School of Public Health<br>Dorjnamjil, Khulan; Mongolian Health Initiative<br>Samdan, Tumurkhuyag; Mongolian Health Initiative<br>Erdenebaatar, Sumiya; Mongolian Health Initiative<br>Chuluun, Altantogoskhon; Ulaanbaatar City Health Department, Governor's Office of Capital City Ulaanbaatar<br>Dalkh, Tserendagva; Mongolian National University of Medical Sciences<br>Ganbaatar, Gantungalag ; National Center for Communicable Diseases, Mongolia<br>Brooks, Meredith; Harvard Medical School, Department of Global Health and Social Medicine<br>Spiegelman, Donna; Yale University School of Public Health, Center for Methods in Implementation and Preventive Science and Department of Biostatistics<br>Ganmaa, Davaasambuu ; Harvard University T H Chan School of Public Health<br>Davis, J.L.; Yale University School of Public Health, Epidemiology of Microbial Diseases; Yale School of Medicine,

Keywords:	Tuberculosis < INFECTIOUS DISEASES, Public health < INFECTIOUS DISEASES, Respiratory infections < THORACIC MEDICINE

SCHOLARONE™
Manuscripts

I, the Submitting Author has the right to grant and does grant on behalf of all authors of the Work (as defined in the below author licence), an exclusive licence and/or a non-exclusive licence for contributions from authors who are: i) UK Crown employees; ii) where BMJ has agreed a CC-BY licence shall apply, and/or iii) in accordance with the terms applicable for US Federal Government officers or employees acting as part of their official duties; on a worldwide, perpetual, irrevocable, royalty-free basis to BMJ Publishing Group Ltd ("BMJ") its licensees and where the relevant Journal is co-owned by BMJ to the co-owners of the Journal, to publish the Work in this journal and any other BMJ products and to exploit all rights, as set out in our licence.

The Submitting Author accepts and understands that any supply made under these terms is made by BMJ to the Submitting Author unless you are acting as an employee on behalf of your employer or a postgraduate student of an affiliated institution which is paying any applicable article publishing charge ("APC") for Open Access articles. Where the Submitting Author wishes to make the Work available on an Open Access basis (and intends to pay the relevant APC), the terms of reuse of such Open Access shall be governed by a Creative Commons licence – details of these licences and which Creative Commons licence will apply to this Work are set out in our licence referred to above.

Other than as permitted in any relevant BMJ Author's Self Archiving Policies, I confirm this Work has not been accepted for publication elsewhere, is not being considered for publication elsewhere and does not duplicate material already published. I confirm all authors consent to publication of this Work and authorise the granting of this licence.

For peer review only

Assessing the Quality of Tuberculosis Care Using Routine Surveillance Data: A process evaluation employing the Zero TB Indicator Framework in Mongolia

Saranjav Ariunzaya, MD*[1], Christina Parisi, MPH*[5,9], Xin Zhou, PhD[6,7], Dorjnamjil Khulan, MD[1], Samdan Tumurkhuyag, BSc[1], Erdenebaatar Sumiya, BSc[1], Chuluun Altantogoskhon, MD[2], Dalkh Tserendagva, PhD[3], Ganbaatar Gantungalag, MPH[4], Meredith B. Brooks, PhD[10], Donna Spiegelman, ScD[6,7], Davaasambuu Ganmaa†, MD[1, 11], J. Lucian Davis†, MD[5,7,8]

*Authors contributed equally; †Authors contributed equally

Corresponding Author:

Dr. Davaasambuu Ganmaa, Department of Nutrition, Harvard T.H. Chan School of Public Health, Bldg. 2, Rm. 211, 655 Huntington Ave., Boston, MA 02115 USA

Affiliations:

[1]Mongolian Health Initiative, [2]Ulaanbaatar City Health Department, Governor's Office of Capital City Ulaanbaatar, [3]International School of Mongolian Medicine, Mongolian National University of Medical Sciences, and [4]National Center for Communicable Disease, Ulaanbaatar, Mongolia;

[5]Departments of Epidemiology of Microbial Diseases and [6]Biostatistics, and the [7]Center for Methods in Implementation and Prevention Science, Yale School of Public Health, and [8]Pulmonary, Critical Care, and Sleep Medicine Section, Yale School of Medicine, New Haven, Connecticut, USA

[9]Department of Epidemiology, University of Florida, Gainesville, Florida, USA

[10]Department of Medicine, Brigham and Women's Hospital, Harvard Medical School, and [11]Department of Nutrition, Harvard School of Public Health, Boston, Massachusetts, USA

For peer review only - http://bmjopen.bmj.com/site/about/guidelines.xhtml

Funding: This work was sponsored by a grant from the National Institute of Environmental Health Sciences (DP1 ES025459 to DLS) and an anonymous foundation based in the United Kingdom. Christina Parisi is funded by NIAAA T32AA025877.

Author Email addresses:

1. Saranjav Ariunzaya, ariunzaya.sar@gmail.com
2. Christina Parisi, christina.parisi@ufl.edu
3. Xin Zhou, xin.zhou@yale.edu
4. Dorjnamjil Khulan, khulan2111@gmail.com
5. Samdan Tumurkhuyag, tumurkhuyagsamdan@gmail.com
6. Erdenebaatar Sumiya, sumiya.mhisumiya@gmail.com
7. Chuluun Altantogoskhon, achuluun95@gmail.com
8. Dalkh Tserendagva, tserendagva_d@yahoo.com
9. Gantungalag Ganbaatar, gantungalag22@gmail.com
10. Meredith Brooks, Meredith_Brooks@hms.harvard.edu
11. Donna Spiegelman, donna.spiegelman@yale.edu
12. Davaasambuu Ganmaa, gdavaasa@hsph.harvard.edu
13. J. Lucian Davis, lucian.davis@yale.edu

Word Count (Limit 5000 words): 3728

Keywords: tuberculosis; implementation science; cascade of care; continuum of care; quality of care; process evaluation

For peer review only

ABSTRACT

Objectives: To evaluate the feasibility of the Zero TB Indicator Framework as a tool for assessing the quality of tuberculosis (TB) case-finding, treatment, and prevention services in Mongolia.

Setting: Primary health centres, tuberculosis dispensaries, and surrounding communities in four districts of Mongolia

Participants: 15,947 community members from high tuberculosis-risk populations; 8,518 patients screened for tuberculosis in primary health centers and referred to dispensaries; 857 index tuberculosis patients and 2352 household contacts

Primary and Secondary Outcome Measures: 14 indicators of the quality of tuberculosis care defined by the Zero TB Indicator Framework; individual and health-system predictors of the quality of TB care.

Results: The cumulative proportions of participants receiving guideline-adherent care varied widely, from 96% for community-based active case-finding, to 79% for TB preventative therapy among household contacts, to only 57% for active case-finding in primary health centers (range 29% to 80% across districts). The odds of patients completing active TB treatment decreased substantially with increasing age (aOR 0.76 per decade, 95%CI 0.71-0.83, p<0.001) and among men (aOR 0.56, 95%CI 0.36-0.88, p=0.013). Contacts of older index patients also had lower odds of initiating and completing of TB preventive therapy (aOR 0.60 per decade, 95%CI 0.38-0.93, p=0.022).

For peer review only - http://bmjopen.bmj.com/site/about/guidelines.xhtml

Conclusions: The Zero TB Framework provided a feasible and adaptable approach for using routine surveillance data to evaluate the quality of TB care and for identifying individual and health system factors associated with the quality of care. Future research should evaluate strategies for collecting process indicators more efficiently; gather qualitative data on explanations for low-quality care; and deploy quality improvement interventions.

Abstract Word Count (Limit 300 words): 255

Strengths and limitations of this study:

- This study provides a comprehensive evaluation of the quality of TB diagnostic, treatment, and prevention service in Mongolia using routine surveillance data.
- This study provides a method for identifying individual and health-system factors associated with lower quality care, which may facilitate targeting of quality improvement interventions.
- Routine tuberculosis (TB) surveillance data is susceptible to outcome misclassification in the absence of unique identifiers.
- Results may not be generalizable to all TB units in Mongolia given the purposive sampling strategy.

For peer review only

For peer review only - http://bmjopen.bmj.com/site/about/guidelines.xhtml

SUMMARY BOX

What is already known?

- Cascade analysis is a form of process evaluation that is increasingly applied to characterize the quality of care for tuberculosis (TB), HIV, and other global infectious diseases.
- We carried did not identify any studies that collected individual-patient data from routine public health databases.

What are the new findings?

This study demonstrate that constructing quality of care cascades by applying the Zero TB Indicator Framework to routine surveillance data is a feasible approach for assessing the quality of TB care and identifying specific processes for improvement. To our knowledge, this is among the first studies to implement an integrated and standardized framework for assessing the overall quality of TB programs by constructing TB care cascades using routinely collected data and defining evidence-based indicators of quality.

What do the new findings imply?

Our work helps advance prior research on TB quality-of-care cascades by demonstrating a particular evaluation tool that is applicable to other high-burden settings that employ WHO's standardized approach to TB monitoring and reporting. Future research and policy initiatives should develop plans for integrating cascade assessments into routine TB surveillance systems and prospectively evaluating their impact on the adoption of strategies for improving the quality of routine TB care in high-burden countries.

For peer review only - http://bmjopen.bmj.com/site/about/guidelines.xhtml

INTRODUCTION

Tuberculosis (TB) incidence and mortality rates have been declining globally for two decades, but with recent global disruptions to TB services and surging poverty rates related to the COVID-19 pandemic, the absolute number of annual TB cases (10 million) and deaths (1.4 million) is likely to increase significantly in coming years.[1] As the global TB community prepares for surging caseloads and declining resources, the crisis in the quality of TB care has never been more salient. Before the pandemic, one-third of TB patients did not access care or were not reported to public health authorities, and even among those who sought care for TB, there was wide geographic variability in treatment coverage and success rates.[2] Other evidence-based strategies for reducing TB incidence, including active case finding[3,4] and TB preventive therapy[5] remain unavailable in most low and middle-income countries.[6] The Lancet Commission on High Quality Health Systems has estimated that almost 50% of annual TB deaths may be attributable to low-quality care.[7]

Although improving access to high-quality services is the ultimate goal, there is first a need for a revolution in measuring and reporting on quality. The general metrics of high-quality care –safety, timeliness, effectiveness, efficiency, equity, patient-centeredness, and integration of services—are widely agreed upon.[8,9] However, these metrics have not been widely adapted or adopted for reporting data from TB programs in real time. For example, national TB programs have traditionally focused public health reporting on treatment coverage and treatment success rates, two narrow measures of effectiveness, and only recently have added process measures such as the proportion of eligible patients initiating TB preventive therapy.[10-12] If improving quality is to be "hard-wired" into national TB programs, as a recent Lancet Commission on Tuberculosis recommended[13], there is an urgent need for research on

For peer review only - http://bmjopen.bmj.com/site/about/guidelines.xhtml

expanding measures of quality for use as benchmarks, as called for by the WHO TB Program[14] and others[15], starting with measures that make better use of routine TB surveillance data.[16]

Structured evaluations of care processes through cascade analyses have increasingly been proposed[15,17-22] and implemented to describe gaps in quality of TB care in low- and middle-income countries, including for clinic-based TB diagnosis and treatment in adults [23-32] and children[33,34], active case-finding[29,35,36], and prevention[29,37,38](Appendix S1). The Zero TB Initiative[39] has introduced an expansive quality indicator framework that integrates all three of these cascade types[19]; similar approaches have been proposed for persons living with HIV.[18] Here, we evaluate the feasibility of the Zero TB Indicator Framework as a tool for assessing the quality of TB services, using data from Mongolia, a Zero TB Initiative country.

METHODS

Setting

Mongolia, a lower middle-income country of 3 million people, has the fourth-highest TB incidence in the WHO Western Pacific Region, at 428 cases per 100,000, and among the world's lowest TB treatment coverage rates, at 31%.[1] Mongolia's high TB prevalence rate(757 per 100,000) exceeds its annual TB incidence rate, indicating a prolonged average duration of illness and the presence of diagnostic delays before treatment.[12,40] More favorably, Mongolia's TB treatment success rate of 91% surpasses the global average, and the country funds 77% of its TB program from domestic sources.[6]

The Mongolian Government provides free TB services in centralized TB dispensaries located in every urban district and rural province. National TB Program(NTP) guidelines recommend TB symptom

For peer review only - http://bmjopen.bmj.com/site/about/guidelines.xhtml

screening of all individuals attending primary health centers and referral of those screening positive to centralized dispensaries for diagnostic evaluation with sputum smear microscopy and chest radiography. Molecular testing is available only in select dispensaries and the National Center for Communicable Diseases, where mycobacterial culture is also available. NTP guidelines recommend active TB case-finding among high-risk groups(people living with HIV; miners; those unhoused, incarcerated, or living in poverty; people living in remote areas; healthcare workers; school staff; orphans) using community-based symptom screening and referral to dispensaries for chest radiography. NTP guidelines also recommend referral of all household contacts of sputum smear-positive TB patients to dispensaries for TB symptom screening and chest radiography, plus tuberculin skin testing(TST); TST-positive children without evidence of active TB are eligible for TB preventive therapy with six months of daily isoniazid.

Study Design, Participants, and Sites

Between January 1 and December 31, 2017, we retrospectively carried out three cross-sectional studies, with two nested longitudinal cohort studies, to construct the three Zero TB Indicator Framework cascades. The Search study examined community-based, active TB case-finding among selected high-risk populations screened by NTP-partner non-governmental organizations(NGOs). The Treat study examined clinic-based, TB diagnosis and treatment among patients screened in primary health centers and referred to dispensaries, including first episodes of care only. Finally, the Prevent study examined diagnosis of latent TB infection(LTBI) and delivery of TB preventive therapy(TPT) among household contacts of index TB patients. The Treat and Prevent studies also included nested longitudinal cohort studies evaluating TB treatment and TPT outcomes, respectively.

For peer review only - http://bmjopen.bmj.com/site/about/guidelines.xhtml

We purposively selected as study sites three TB dispensaries in and around the capital city of Ulaanbaatar(Chingeltei, Bayanzurkh, and Khan-Uul) that draw from densely populated residential areas with both urban and rural economic contexts. We also selected one general hospital located outside the capital in Mandal *soum*(sub-province) within rural Selenge province.

Measurements

Mongolian Health Initiative staff(S.A., D. K., S.T., E.S.) digitally photographed official NTP laboratory, treatment, and contact registers during site visits. They double-entered demographic and clinical data on possible TB patients, confirmed TB patients, and household TB contacts from these photos into electronic data collection forms(REDCap, Nashville, TN, USA). They also extracted aggregated data on active case-finding from NGO reports to the NTP, and on TB symptom screening in primary health centers from records of the Mongolian Association of Family Medicine Specialists.

We successfully defined and adapted 14 of 16 Zero TB Indicators to fit data routinely collected by the Mongolia NTP and partners,[19] and proposed evidence-based performance targets derived from published guidelines or systematic reviews(Table 1). We could not obtain data on who was TB-free one year after completing active TB treatment or TPT because post-treatment follow-up is not routinely performed in Mongolia.

For peer review only - http://bmjopen.bmj.com/site/about/guidelines.xhtml

Table 1 – Process indicators of performance, adapted for Mongolia from the Zero TB Indicator Framework.

Indicator* (Proportion)	Numerator (Count)	Denominator (Count)	Interpretation	Target/ Source
**SEARCH CASCADE**				
1. (Screening) Coverage	Screened for possible active TB disease	Estimated individuals in target population	Effectiveness of outreach to target population	100%[63]
2. Positive TB symptom screen	Positive TB symptom or CXR screening	Screened for active TB disease	Accuracy & efficiency of screening implementation	25-50%[63]
3. Diagnostic evaluation	Completing TB evaluation	Positive TB symptom or CXR screening	Effectiveness of linkage to testing	100%[63]
4. (Active) TB Diagnosis	Diagnosed with active TB disease	Completed TB evaluation	Yield of diagnostic evaluation	10-20%[64]
5. Linkage to TB Treatment	Beginning active TB treatment	Diagnosed with active TB disease	Effectiveness of linkage to treatment	100%[65]
**TREAT CASCADE**				
1. (Active) TB Diagnosis	Diagnosed with active TB disease	Evaluated for active TB disease	Yield of diagnostic evaluation	10-20%[64]
2. Bacteriologic Confirmation	*Mtb*-positive bacteriologic test	Diagnosed with active TB disease	Accuracy & efficiency of screening implementation	70-90%[66,67]
3. Linkage to TB Treatment	Started active TB treatment	Diagnosed with active TB disease	Effectiveness of linkage to treatment	100%[65]
4. Treatment Success	Treatment outcome successful	Started active TB treatment	Effectiveness of treatment	≥90%[11]
5. TB-free Survival	TB-free one year later	Treatment outcome successful	Effectiveness of treatment follow-up	≥95%[55]
**PREVENT CASCADE**				
TB Screening	Completing TB screening	Estimated household contacts of smear-positive index TB patients	Effectiveness of outreach to target population	100%[68]
1. TB Evaluation	Completed TB clinical/lab evaluation	Completed TB screening	Effectiveness of linkage to testing	≥90%[11]
2. (Active) TB Diagnosis	Diagnosed with active TB disease	Completed TB evaluation	Yield of diagnostic evaluation	10-20%[64]
TPT Eligibility	Confirmed not to have active TB disease & TST-positive†	Completed TB evaluation	Accuracy & efficiency of testing implementation	95%[5]
3. (TPT) Prescription	Prescribed preventive therapy	Eligible for preventive therapy	Adoption of TPT by providers	≥90%[11]
4. (TPT) Uptake	Started preventive therapy	Prescribed preventive therapy	Reach of TPT to patients	100%[11]
5. (TPT) Completion	Completed preventive therapy	Started preventive therapy	Effectiveness of TPT implementation	≥80%[5]
6. TB-free survival	TB-free one year later	No TB present at initial evaluation	Effectiveness of treatment follow-up	100%[5]

Abbreviations: CXR, chest radiograph; TB, tuberculosis; TPT, TB preventive therapy; TST, tuberculin skin test

Legend: Indicators and cascades derived from the Zero TB Indicator Framework, with numerators adapted using the Mongolian National TB Guidelines and source data, and targets derived from WHO guidelines and targets. Additional details available in Appendix S1. *Indicator numbers as proposed by the Zero TB Indicator Framework[19]; †Mongolian national guidelines require TST positivity, while WHO guidelines recommend that TPT decisions do not require TST or interferon-gamma release assay (IGRA) testing.

For peer review only - http://bmjopen.bmj.com/site/about/guidelines.xhtml

Analysis

We presented baseline demographic and clinical characteristics of those completing referral for TB evaluation for the Treat and Prevent studies only; demographic and clinical characteristics were not available for the Search study. We calculated proportions for dichotomous variables and medians with upper and lower quartiles for continuous variables.

For the process evaluation, we determined indicators for the Search, Treat, and Prevent cascades differently. The Search and Treat cascade indicators were calculated as simple proportions, and the yield as counts diagnosed and initiating treatment divided by total counts screened, with the number-needed-to-screen reported as the inverse of this ratio. Within the Prevent Cascade, we assessed for correlation of process outcomes among contacts within households by calculating Intraclass Correlation Coefficients(ICC). For outcomes with ICC ≥0.10, we estimated the probability of completion by fitting a multivariate, logistic regression model using generalized estimating equations(GEE),[41] with an exchangeable working correlation structure to account for households. For each cascade, we presented indicators within flow diagrams(Figure 1), and calculated stepwise and cumulative probabilities of receiving guideline-adherent care. For the Treat Cascade, we also calculated site-level cumulative probabilities of symptomatic individuals at primary health centers being referred to a dispensary, evaluated for active TB, and starting and completing treatment if diagnosed. We derived a formula to estimate 95% confidence intervals for cumulative probabilities (Appendix S2). We assessed between-site differences in performance using the chi-squared test of independence. Finally, for process outcomes with the greatest losses, we constructed multivariate, logistic regression models using GEE to identify individual demographic (age, gender) predictors of dropout, with 95% CI and p-values provided by Wald tests.

For peer review only

For peer review only - http://bmjopen.bmj.com/site/about/guidelines.xhtml

We based sample size on available participants. We performed all analyses in SAS(Version 9.4, The SAS Institute, Cary, NC, USA).

Patient and Public Involvement in Research

Patients who had currently or previously had TB were not involved in defining the research design or implementation, but members of the research team (S.A., D.K.) met with staff at the primary health centers and the dispensaries intermittently during data collection to refine the study measures and to disseminate the research findings.

For peer review only

For peer review only - http://bmjopen.bmj.com/site/about/guidelines.xhtml

RESULTS

Search Cascade

There were 15,947 people in high-risk populations screened for active TB within the four study communities, including 6,670(42%) in Chingeltei district, 4,632(29%) in Bayanzurkh district, 3,865(24%) in Khan-Uul district, and 780(5%) in Mandal sub-province. This included 12,082(76%) vulnerable individuals of low socioeconomic status in Chingeltei and Bayanzurkh districts and in Mandal sub-province; and 3,865(24%) students, teachers, and administrative officials in Khan-Uul district. Of the original 15,947, 15,294(96%) underwent symptom screening and chest radiography for possible active TB disease(Figure 2). Screening was positive for 1,592(10%). Of these, 1,571(99%) completed TB evaluation, with 97(6%) diagnosed with active TB disease and 89(92%) starting treatment.

Of all 15,947 community members screened for active TB in the Search Cascade, 15,261(95.7%) received all services defined as indicative of high-quality care (Figure 5a). The cumulative yield of active TB treatments among these communities was 558 per 100,000 screened, providing a number-needed-to-screen of 179.

Treat Cascade

There were 8,518 people who screened positive for TB symptoms at 62 primary health care centers spread across the four communities(Figure 3), including 3245(38%) from Bayanzurkh, 2620(31%) from Khan-Uul, 2160(25%) from Chingeltei, and 493(6%) from Mandal. Of the 8,518 individuals screened, 1,486(17%) were assigned non-TB diagnoses. The remaining 7,032(83%) were referred to dispensaries for TB evaluation, but only 4,416(63%) arrived and underwent TB testing. Those tested included 2,348 males(53%) and four people living with HIV(0.1%). Median age was 40 years(lower quartile(Q1) 26 to upper quartile(Q3) 55). Of those visiting the dispensaries and tested, 1,162(26%) were diagnosed with

For peer review only - http://bmjopen.bmj.com/site/about/guidelines.xhtml

active TB, 824(71%) of whom were bacteriologically confirmed. Of these, 192(23%) had drug-resistant TB. Among the 1,124(97%) who began treatment, 868(77%) were successfully treated, including 81% of drug-susceptible and 55% of drug-resistant cases. Among those who did not complete treatment, 94(8%) were lost to follow up, 52(5%) failed treatment, 31(3%) died, and 79(7%) had unknown treatment outcomes.

Of all 8,518 individuals entering the Treat Cascade by screening positive for TB symptoms at primary health centers, 5,726(67.2%) received all services defined as indicative of high-quality care (Figure 5b).

Treat cascade indicators showed significant variation across sites(Table S0). Among those referred from primary health centers, evaluation rates ranged widely, from nearly 100% in Mandal *soum* to just 47% in Bayanzurkh(Chi-squared test for independence, p<0.001). The proportions starting treatment, in contrast, were consistently high, from 93% to 98% across sites(p=0.090). Finally, treatment success varied markedly, with a high of 87% in Mandal *soum* to a low of 72% in Bayanzurkh(p<0.001). The cumulative probabilities of being properly diagnosed and treated for TB given a positive TB diagnosis also varied substantially(p<0.001) and were highest at Mandal *soum*(80%, 95% CI 68%-92%) and lowest at Bayanzurkh(29%, 95% CI 27%-31%), with similarly low probabilities at Khan-Uul(42%, 95% CI 40%-45%) and Chingeltei(44%, 95% CI 41%-47%).

The odds of starting treatment among active TB patients were lower among older individuals(adjusted Odds Ratio(aOR) 0.72 per decade, 95%CI 0.66-0.79, p<0.001) and males(aOR 0.73, 95%CI 0.62-0.86, p<0.001)(Table S1). The odds of completing TB treatment were also lower in older individuals(aOR 0.76 per decade; 95% CI 0.71-0.83, p<0.001), and males(aOR 0.56; 95% CI 0.36-0.88, p=0.013)(Table S2).

Prevent Cascade

For peer review only - http://bmjopen.bmj.com/site/about/guidelines.xhtml

Among 857 index patients diagnosed with smear-positive active TB, 75 lived alone or did not report on household contacts, leaving 782(91%) eligible index patients with 2,352 household contacts (mean 3 household contacts per household) eligible for contact investigation. Among contacts in the same household, there was modest correlation in eligibility for TPT(ICC=0.14), and a moderate-to-high correlation for evaluation completion(ICC=0.30) and prescription and initiation of TPT(ICC=0.41).

Of the 2352 household contacts in the target population, including 270 children under 5 years old, only 1932(82%, 95%CI 80-84%) visited the dispensary and were evaluated for active TB (Figure 4).

Older patients were less likely to complete TB evaluation(aOR 0.92 per decade, 95%CI 0.86-0.98, p=0.015), after adjusting for the gender of the contact, the contact's relationship to index patient, and the age and gender of the index patient(Table S3).

Of the household contacts who reached the dispensary, 179(9%) were diagnosed with active TB, yielding 7,600 cases per 100,000, or a number-needed-to-screen among all household contacts of 13. Concurrently, 1753(91%) contacts were found not to have active TB, including 254(15%; 95%CI 13-16%) under 5 years of age who were eligible for screening for latent TB infection(LTBI). Eighty-six(34%; 95%CI 29-41%) child contacts were tuberculin skin test(TST) positive. Among these, 36(43%; 95%CI 33-55%) were prescribed and initiated IPT and all 36(100%) completed TPT. Of all 8,518 individuals entering the Prevent Cascade as household contacts being screened for active TB, 1868(79.4%) received all services defined as indicative high-quality care (Figure 5c).

Older index patient age was associated with substantially lower odds of an eligible child being prescribed preventive therapy(aOR 0.60 per decade, 95%CI 0.38-0.93, p=0.022), after adjusting for age and gender of the contact, the contact's relationship to index patient, and gender of the index patient(Table S4).

For peer review only - http://bmjopen.bmj.com/site/about/guidelines.xhtml

DISCUSSION

We systematically evaluated the quality of TB care at the sub-national and facility levels by applying the Zero TB Indicator Framework to routinely collected TB surveillance data from four administrative regions of Mongolia. Overall, the quality of care in the Search Cascade was high, with almost everyone receiving all recommended active case-finding services. In contrast, the quality of care in the Prevent Cascade was only moderate-to-high, with only four out of every five receiving all recommended household TB screening and prevention services, and the quality of care in the Treat Cascade was only moderate, with only two out of every three receiving all recommended passive case finding and treatment services. The exercise also enabled us to localize important gaps in the efficiency, individual and public health effectiveness, and equity of TB active and passive case finding and prevention in reference to evidence-based standards. In addition, we identified individual and facility-level factors associated with poor outcomes, providing a starting point for quality improvement interventions.

This work is an important contribution in several ways. First, it is among the first efforts to assimilate analyses of quality across the spectrum of TB services, including TB active case-finding, TB diagnosis and treatment, and TB preventive therapy simultaneously. Second, the cascades were constructed directly using routinely collected data, without the requirement for intermediate epidemiological assumptions, an important advance toward the goal of integrating actionable, real-time quality assessments into routine TB surveillance at local level. Third, using the Zero TB Indicator Framework to achieve this goal provided the opportunity to evaluate its feasibility as a tool for monitoring the quality of TB care in a resource-constrained, high TB-incidence country, a critical need in global public health.

For peer review only - http://bmjopen.bmj.com/site/about/guidelines.xhtml

In addition, our analysis illustrates how the systematic evaluation of care processes using the Zero TB Indicator framework could enable national programs to identify areas for additional evaluation and quality improvement activities. First, in Mongolia, although 96% of community members invited to screen in the “Search” cascade ultimately received all recommended services, the cumulative effectiveness as inferred from the diagnostic and treatment yield was unexpectedly low, barely exceeding the national TB prevalence rate. The low yield is partly attributable to the use of sputum smear microscopy for confirmatory testing, a low sensitivity diagnostic test in the context of active-case finding for early stage disease, rather than mycobacterial culture or molecular methods as recommended in international guidelines.[42] These higher quality diagnostics remain prohibitively costly, even in middle-income countries; continued efforts are needed to increase financial commitments to advance TB elimination efforts by implementing internationally recommended diagnostics. Also, the absolute number of participants reached and the additional cases identified by these small, short-term active case-finding initiatives were modest, pointing to the need to better target active case-finding towards TB “hotspots”[43] and to sustain screening efforts over time.[4] In contrast, the yield of active TB case finding among household contacts in the Prevent Cascade was an order of magnitude higher and contact investigation also facilitated TB prevention efforts, potentially offering much greater value.

Second, we identified the largest gaps in quality of care within the Treat cascade, where small numbers of patients dropping out from individual care processes accumulated into large losses across the cascade. In Mongolia’s centralized system for TB evaluation, one-third of symptomatic individuals did not reach diagnostic centers, a metric that varied widely by geographic area. Even after patients reached TB dispensaries, diagnostic yield and treatment completion also varied widely. While one low-volume, rural site delivered effective diagnosis and treatment to 80% of those presenting for evaluation, only one-quarter to one-half of patients at the other three sites completed all of the Treat Cascade steps that

For peer review only - http://bmjopen.bmj.com/site/about/guidelines.xhtml

we defined as high quality of care. In addition, treatment completion was below the national average at all four sites, especially among older adults and men, who were also less likely to initiate treatment than younger adults and women.[6] These data suggest a need to further investigate patient-level barriers to accessing diagnostics and treatment at Mongolia's centralized TB dispensaries. In a systematic review of nearly 60 studies of TB diagnostic and treatment delay worldwide, initial presentation to low-level government facilities, geographic isolation, and poverty were three of the most important risk factors.[44] According to a 2017 Mongolia NTP survey, >50% TB patients are unemployed and 70% have incomes below the poverty line.[19] If our data are representative of the broader symptomatic population identified at primary health centers, as found elsewhere,[45,46] travel and related opportunity costs may be prohibitive to completing referrals and treatment. Decentralizing TB care to primary health centers is one policy intervention to be considered. In a small study from Southern Brazil, a variety of diagnostic and treatment outcomes were better in a municipality with decentralized care than in a comparable municipality with centralized care.[47] In central Sulawesi, Indonesia, a TB program that began offering education, evaluation, and treatment in a community-based setting tripled their case-detection rates relative to baseline, and also increased treatment completion considerably.[48]

Third, in our evaluation of the Prevent cascade, almost 80% of household contacts completed TB evaluation, approaching the WHO's 90% target.[49] This compares favorably to the 20-53% who completed contact investigation in prior studies, and the number needed to screen of 13 was far below the 56 reported in a prior systematic review.[35,36,50,51] Nevertheless, over half of child contacts eligible for TPT did not start it, similar to results reported elsewhere[51-53], and the likelihood of initiation decreased substantially with increasing age of index patients. In a prior systematic review[37], a low perceived risk of active TB for one's children was among the most frequent reasons for low uptake, emphasizing the need for personalized education and counseling to address parental concerns. In addition, high rates of TPT

For peer review only - http://bmjopen.bmj.com/site/about/guidelines.xhtml

completion among a small group children and high rates of referral completion among adults suggest additional capacity to expand TPT in Mongolia to older child and adult contacts, as recommended by the 2018 WHO guidelines.[5]

Strengths

Our study had several strengths. Most prior studies have focused on only one specific aspect of TB services such diagnosis and treatment, or prevention. In contrast, we adopted a more comprehensive perspective[20], evaluating multiple evidence-based interventions concurrently using 14 indicators within the Zero TB Indicator Framework. We successfully adapted the indicators to fit local data and guidelines, and developed evidence-based targets as comparators. Second, building on previous studies that relied on research data, aggregated national data, or incorporated epidemiologic assumptions about TB prevalence,[54] we generated cascades directly from data collected at the basic TB management unit and referring diagnostic centers. We believe this approach could help address the global need to operationalize standardized measures of quality within routine data collection systems, so that cascades may be actionable at facility and district level.[14,27] Although this study's manual extraction procedures would not be feasible for routine use, our approach could help justify adapting routine TB registers to include process measures of quality. Third, we identified individual and health-system factors associated with failure to complete key steps of the cascade, providing important information for tailoring future quality improvement interventions.

For peer review only - http://bmjopen.bmj.com/site/about/guidelines.xhtml

Limitations

Our study also had some limitations. First, we were unable to evaluate one-year post-completion outcomes of active TB treatment and TB preventative therapy as called for by the Zero TB indicator Framework. Although this follow-up data is important for evaluating effectiveness, few programs in high-incidence, resource-constrained countries collect it, and the feasibility and costs of doing so must be considered given low rates of relapse of active TB[55] or progression of latent TB after treatment completion.[5] Second, our study sites may not be representative of all sites in Mongolia, given our purposive selection strategy. However, evaluating a large random sample was beyond the scope of this preliminary evaluation, and so we selected communities representing different population densities, housing types, income levels, and economic settings. Third, some records may have been duplicated because we relied on aggregated data for some analyses and lacked unique identifiers to link different visits to the same individual. Similarly, some outcome misclassification may be related to inaccurate tracking of handwritten records across multiple official primary data sources, and some records were incomplete. These challenges highlight the need to standardize and expand use of Mongolia's nascent electronic TB registration system(*i.e.,* TUBIS.mn), to improve both the quality of reporting and the quality of care. In Zambia, for example, regular review and cross-validation of TB registers at diagnostic and treatment units was associated with significant reductions in the proportion of non-assessed cases.[56]

Conclusion

We found that using standardized treatment indicators and cascades to evaluate core processes of evidence-based TB interventions was feasible and informative for evaluating the quality of TB services. This approach may be generalizable to other high-burden settings that employ WHO's standardized

For peer review only - http://bmjopen.bmj.com/site/about/guidelines.xhtml

approach to TB monitoring and reporting.[57,58] To complement such analyses, there is a need to incorporate qualitative and mixed-methods studies[32,59,60] to contextualize the barriers and facilitators of care processes, as well as structural assessments of quality such as service availability and readiness surveys[61], to help enhance the outcomes of TB care.[62] Above all, it is time for researchers and practitioners to strengthen routine TB surveillance data systems to collect rigorous, accessible, and reliable data on the quality of TB services worldwide.

For peer review only

For peer review only - http://bmjopen.bmj.com/site/about/guidelines.xhtml

ACKNOWLEDGMENTS

The authors wish to acknowledge the Mongolian National TB Program; doctors, nurses, and staff at the primary health centers and the TB dispensaries who provided the care under evaluation in this project; the patients and community members whose data contributed to this analysis; Courtney Yuen, Mercedes Becerra, and Michael Wilson for their assistance in establishing the Zero TB Initiative in Mongolia; the Mongolian Association of Family Medicine Specialists, and the staff of the Mongolian Health Initiative.

For peer review only

AUTHOR CONTRIBUTIONS

1. Conceptualisation: JLD, DG, DS
2. Data Curation: SA, CP, DK, ST, CA
3. Formal Analysis: SA, CP, XZ, JLD
4. Funding Acquisition: DS
5. Methodology: JLD, XZ, DLS
6. Project Administration: SA, DG, JLD, GG, DT, CA, MB
7. Verification of Underlying Data: SA, DK, DG, ES
8. Visualisation: CP, JLD
9. Writing – Original Draft: SA, CP, JLD
10. Writing – Review & Editing: SA, CP, XZ, DK, ST, ES, CA, DT, GG, MB, DS, DG, JLD

Legend

Saranjav Ariunzaya (SA)

Christina Parisi (CP)

Xin Zhou (XZ)

Dorjnamjil Khulan (DK)

Samdan Tumurkhuyag (ST)

Erdenebaatar Sumiya (ES)

Chuluun Altantogoskhon (CA)

Dalkh Tserendagva (DT)

Ganbaatar Gantungalag (GG)

Meredith Brooks (MB)

Donna Spiegelman (DS)

Davaasambuu Ganmaa (DG)

J. Lucian Davis (JLD)

For peer review only

For peer review only - http://bmjopen.bmj.com/site/about/guidelines.xhtml

Funding Statement

This work was sponsored by a grant from the National Institute of Environmental Health Sciences (DP1 ES025459 to DLS) and an anonymous foundation based in the United Kingdom. Christina Parisi is funded by NIAAA T32AA025877. The study funders had no role in design, collection, analysis, or interpretation of the data, in writing this report, or in the decision to submit it for publication.

Conflict of Interest Statement

The authors declare that there is no conflict of interest.

Data Availability Statement

The data that support the findings of this study are available from the corresponding author, Ariunzaya Saranjav, upon reasonable request.

Ethics Approval Statement

Institutional review boards at Yale University (#2000025951), the Harvard School of Public Health (#19-1489), and the Mongolian National University of Medical Sciences (#2019/3-10) approved the study protocol as minimal risk research and waived requirements for informed consent.

For peer review only

REFERENCES

1. World Health Organization. Global tuberculosis control: WHO Report 2020. Geneva: World Health Organization; 2020.

2. Cattamanchi A, Berger CA, Shete PB, et al. Implementation science to improve the quality of tuberculosis diagnostic services in Uganda. *Journal of Clinical Tuberculosis and Other Mycobacterial Diseases* 2020; **18**: 100136.

3. Corbett EL, Bandason T, Duong T, et al. Comparison of two active case-finding strategies for community-based diagnosis of symptomatic smear-positive tuberculosis and control of infectious tuberculosis in Harare, Zimbabwe (DETECTB): A cluster-randomised trial. *Lancet* 2010; **376**(9748): 1244-53.

4. Marks GB, Nguyen NV, Nguyen PTB, et al. Community-wide Screening for Tuberculosis in a High-Prevalence Setting. *N Engl J Med* 2019; **381**(14): 1347-57.

5. World Health Organization. Latent tuberculosis infection: Updated and consolidated guidelines for programmatic management. Geneva: World Health Organization; 2018.

6. World Health Organization. Global tuberculosis control: WHO Report 2020. Geneva: World Health Organization, 2020.

7. Kruk ME, Gage AD, Arsenault C, et al. High-quality health systems in the Sustainable Development Goals era: time for a revolution. *The Lancet Global Health* 2018; **6**(11): e1196-e252.

8. Delivering Quality Health Services: A Global Imperative for Universal Health Coverage. Geneva: World Health Organization, Organisation for Economic Co-operation and Development, and The World Bank; 2018.

9. Improving Quality of Care in Low- and Middle-Income Countries: Workshop Summary. Washington, DC: The National Academies Press; 2015.

For peer review only - http://bmjopen.bmj.com/site/about/guidelines.xhtml

10. World Health Organization. Framework for conducting reviews of tuberculosis programmes: World Health Organization; 2014.

11. World Health Organization. The End TB Strategy. Geneva: World Health Organization; 2015.

12. World Health Organization. Global tuberculosis control: WHO Report 2019. Geneva: World Health Organization, 2019.

13. Reid MJA, Goosby E. Improving quality is necessary to building a TB-free world: Lancet Commission on Tuberculosis. *Journal of clinical tuberculosis and other mycobacterial diseases* 2020; **19**: 100156-.

14. Floyd K, Glaziou P, Houben RMGJ, Sumner T, White RG, Raviglione M. Global tuberculosis targets and milestones set for 2016-2035: Definition and rationale. *International Journal of Tuberculosis and Lung Disease* 2018; **22**(7): 723-30.

15. Subbaraman R, Nathavitharana RR, Mayer KH, et al. Constructing care cascades for active tuberculosis: A strategy for program monitoring and identifying gaps in quality of care. *PLoS Med* 2019; **16**(2): e1002754.

16. Theron G, Jenkins HE, Cobelens F, et al. Data for action: collection and use of local data to end tuberculosis. *The Lancet* 2015; **386**(10010): 2324-33.

17. Daftary A, Jha N, Pai M. Enhancing the role of pharmacists in the cascade of tuberculosis care. *Journal of Epidemiology and Global Health* 2016; **7**(1): 1.

18. Hanrahan CF, Van Rie A. A proposed novel framework for monitoring and evaluation of the cascade of HIV-associated TB care at the health facility level. *Journal of the International AIDS Society* 2017; **20**(1): 21375.

19. Yuen C, Becerra M, Codlin A, et al. A best-practice framework of program indicators for monitoring a comprehensive approach to the tuberculosis epidemic: Zero TB Initiative, 2017.

For peer review only

For peer review only - http://bmjopen.bmj.com/site/about/guidelines.xhtml

20. Cazabon D, Alsdurf H, Satyanarayana S, et al. Quality of tuberculosis care in high burden countries: the urgent need to address gaps in the care cascade. *Int J Infect Dis* 2017; **56**: 111-6.

21. Agins BD, Ikeda DJ, Reid MJA, Goosby E, Pai M, Cattamanchi A. Improving the cascade of global tuberculosis care: moving from the "what" to the "how" of quality improvement. *The Lancet Infectious Diseases* 2019; **19**(12): e437-e43.

22. Arsenault C, Roder-DeWan S, Kruk ME. Measuring and improving the quality of tuberculosis care: A framework and implications from the Lancet Global Health Commission. *J Clin Tuberc Other Mycobact Dis* 2019; **16**: 100112.

23. Davis JL, Katamba A, Vasquez J, et al. Evaluating tuberculosis case detection via real-time monitoring of tuberculosis diagnostic services. *Am J Respir Crit Care Med* 2011; **184**(3): 362-7.

24. Davis JL, Dowdy DW, den Boon S, Walter ND, Katamba A, Cattamanchi A. Test and treat: a new standard for smear-positive tuberculosis. *J Acquir Immune Defic Syndr* 2012; **61**(1): e6-e8.

25. Sun AY, Denkinger CM, Dowdy DW. The impact of novel tests for tuberculosis depends on the diagnostic cascade. *Eur Respir J* 2014; **44**(5): 1366-9.

26. Chaisson LH, Katamba A, Haguma P, et al. Theory-informed interventions to improve the quality of tuberculosis evaluation at Ugandan health centers: a quasi-experimental study. *PloS one* 2015; **10**(7): e0132573.

27. Subbaraman R, Nathavitharana RR, Satyanarayana S, et al. The Tuberculosis Cascade of Care in India's Public Sector: A Systematic Review and Meta-analysis. *PLOS Medicine* 2016; **13**(10): e1002149.

28. Naidoo P, Theron G, Rangaka MX, et al. The South African Tuberculosis Care Cascade: Estimated Losses and Methodological Challenges. *J Infect Dis* 2017; **216**(suppl_7): S702-s13.

29. Shapiro AE, van Heerden A, Schaafsma TT, et al. Completion of the tuberculosis care cascade in a community-based HIV linkage-to-care study in South Africa and Uganda. *J Int AIDS Soc* 2018; **21**(1).

For peer review only - http://bmjopen.bmj.com/site/about/guidelines.xhtml

30. Kim J, Keshavjee S, Atun R. Health systems performance in managing tuberculosis: analysis of tuberculosis care cascades among high-burden and non-high-burden countries. *J Glob Health* 2019; **9**(1): 010423.

31. Der JB, Grint D, Narh CT, Bonsu F, Grant AD. Where are patients missed in the tuberculosis diagnostic cascade? A prospective cohort study in Ghana. *PloS one* 2020; **15**(3): e0230604.

32. Yasobant S, Bhavsar P, Kalpana P, Memon F, Trivedi P, Saxena D. Contributing Factors in the Tuberculosis Care Cascade in India: A Systematic Literature Review. *Risk Management and Healthcare Policy* 2021; **Volume 14**: 3275-86.

33. Marquez C, Davis JL, Katamba A, et al. Assessing the quality of tuberculosis evaluation for children with prolonged cough presenting to routine community health care settings in rural Uganda. *PloS one* 2014; **9**(8): e105935.

34. Mwangwa F, Chamie G, Kwarisiima D, et al. Gaps in the Child Tuberculosis Care Cascade in 32 Rural Communities in Uganda and Kenya. *J Clin Tuberc Other Mycobact Dis* 2017; **9**: 24-9.

35. Armstrong-Hough M, Turimumahoro P, Meyer AJ, et al. Drop-out from the tuberculosis contact investigation cascade in a routine public health setting in urban Uganda: A prospective, multi-center study. *PloS one* 2017; **12**(11): e0187145.

36. Diaz G, Victoria AM, Meyer AJ, et al. Evaluating the Quality of Tuberculosis Contact Investigation in Cali, Colombia: A Retrospective Cohort Study. *Am J Trop Med Hyg* 2021.

37. Alsdurf H, Hill PC, Matteelli A, Getahun H, Menzies D. The cascade of care in diagnosis and treatment of latent tuberculosis infection: a systematic review and meta-analysis. *Lancet Infect Dis* 2016; **16**(11): 1269-78.

38. van de Water B, Meyer T, Wilson M, Young C, Gaunt B, le Roux K. TB prevention cascade at a district hospital in rural Eastern Cape, South Africa. *Public Health Action* 2021; **11**(2): 97-100.

For peer review only

39. Zero TB Initiative. The Zero TB Initiative. 2021. https://www.zerotbinitiative.org/ (accessed January 10 2021).

40. Mongolia Ministry of Health, National Center for Communicable Diseases. Report of the First National Tuberculosis Prevalence Survey in Mongolia, 2014-2015. Ulaanbaatar, Mongolia; 2016.

41. Diggle P, Diggle PJ, Heagerty P, Liang K-Y, Heagerty PJ, Zeger S. Analysis of longitudinal data: Oxford University Press; 2002.

42. World Health Organization. Tuberculosis Diagnostics. Geneva: World Health Organization, 2016.

43. Shrestha S, Reja M, Gomes I, et al. Quantifying geographic heterogeneity in TB incidence and the potential impact of geographically targeted interventions in South and North City Corporations of Dhaka, Bangladesh: a model-based study. *Epidemiology and Infection* 2021; **149**: 1-27.

44. Storla DG, Yimer S, Bjune GA. A systematic review of delay in the diagnosis and treatment of tuberculosis. *BMC Public Health* 2008; **8**(1): 15.

45. Liu X, Thomson R, Gong Y, et al. How affordable are tuberculosis diagnosis and treatment in rural China? An analysis from community and tuberculosis patient perspectives. *Tropical Medicine & International Health* 2007; **12**(12): 1464-71.

46. Xu L, Gai R, Wang X, et al. Socio-economic factors affecting the success of tuberculosis treatment in six counties of Shandong Province, China. *The international journal of tuberculosis and lung disease : the official journal of the International Union against Tuberculosis and Lung Disease* 2010; **14**(4): 440-6.

47. Scheffer MC, Prim RI, Wildner LM, et al. Performance of centralized versus decentralized tuberculosis treatment services in Southern Brazil, 2006–2015. *BMC Public Health* 2018; **18**(1): 554.

48. Becx-Bleumink M, Wibowo H, Apriani W, Vrakking H. High tuberculosis notification and treatment success rates through community participation in central Sulawesi, Republic of Indonesia. *International Journal of Tuberculosis and Lung Disease* 2001; **5**(10): 920-5.

For peer review only - http://bmjopen.bmj.com/site/about/guidelines.xhtml

49. World Health Organization. Implementing the End TB strategy: The essentials. Geneva: World Health Organization, 2015.

50. Kigozi G, Engelbrecht M, Heunis C, Janse van Rensburg A. Household contact non-attendance of clinical evaluation for tuberculosis: A pilot study in a high burden district in South Africa. *BMC Infectious Diseases* 2018; **18**(1): 106.

51. Blok L, Sahu S, Creswell J, Alba S, Stevens R, Bakker MI. Comparative meta-analysis of tuberculosis contact investigation interventions in eleven high burden countries. *PloS one* 2015; **10**(3): e0119822.

52. Singh AR, Kharate A, Bhat P, et al. Isoniazid preventive therapy among children living with tuberculosis patients: Is it working? A mixed-method study from Bhopal, India. *Journal of Tropical Pediatrics* 2017; **63**(4): 274-85.

53. Yuen CM, Millones AK, Puma D, et al. Closing delivery gaps in the treatment of tuberculosis infection: lessons from implementation research in Peru. *PloS one* 2021; **16**(2): e0247411.

54. Subbaraman R, Jhaveri T, Nathavitharana RR. Closing gaps in the tuberculosis care cascade: An action-oriented research agenda. *Journal of clinical tuberculosis and other mycobacterial diseases* 2020; **19**: 100144-.

55. Imperial MZ, Nahid P, Phillips PPJ, et al. A patient-level pooled analysis of treatment-shortening regimens for drug-susceptible pulmonary tuberculosis. *Nat Med* 2018; **24**(11): 1708-15.

56. Mfungwe V, Ota M, Koyama K, et al. 'Transfer out' tuberculosis patients: Treatment outcomes after cross-checking registers, 2012-2013, Lusaka, Zambia. *Public Health Action* 2016; **6**(2): 118-21.

57. World Health Organization. Standards and benchmarks for tuberculosis surveillance and vital registration systems. Geneva: World Health Organization; 2014.

58. World Health Organization. Toolkit for Analysis and Use of Routine Health Facility Data: Guidance for tuberculosis programme managers. Geneva: World Health Organization; 2020.

For peer review only - http://bmjopen.bmj.com/site/about/guidelines.xhtml

59. Armstrong-Hough M, Ggita J, Ayakaka I, et al. Brief Report: "Give Me Some Time": Facilitators of and Barriers to Uptake of Home-Based HIV Testing During Household Contact Investigation for Tuberculosis in Kampala, Uganda. *J Acquir Immune Defic Syndr* 2018; **77**(4): 400-4.

60. Armstrong-Hough M, Ggita J, Turimumahoro P, et al. 'Something so hard': a mixed-methods study of home sputum collection for tuberculosis contact investigation in Uganda. *The international journal of tuberculosis and lung disease : the official journal of the International Union against Tuberculosis and Lung Disease* 2018; **22**(10): 1152-9.

61. World Health Organization. Service Availability and Readiness Assessment (SARA): an annual monitoring system for service delivery. In: Health Statistics and Information Systems, editor. Geneva: World Health Organization; 2015.

62. Donabedian A. The quality of Care: How can it be assessed? *JAMA* 1988; **260**(12).

63. World Health Organization. WHO operational handbook on tuberculosis. Module 2: Screening. Systematic screening for tuberculosis disease. Geneva: World Health Organization; 2021.

64. Stop TB Partnership. Laboratory diagnosis of tuberculosis by sputum microscopy. In: Global Laboratory Initiative, editor. Adelaide, Australia: SA Pathology; 2013.

65. World Health Organization. WHO consolidated guidelines on tuberculosis: module 4: treatment: drug-resistant tuberculosis treatment: World Health Organization; 2020.

66. World Health Organization. Policy statement: The use of liquid medium for culture and DST in low- and middle-income country settings. Geneva: World Health Organization; 2007.

67. World Health Organization. WHO Meeting Report of a Technical Expert Consultation: Non-inferiority analysis of Xpert MTB/RIF Ultra compared to Xpert MTB/RIF Geneva: World Health Organization; 2017.

68. World Health Organization. Recommendations for investigating contacts of persons with infectious tuberculosis in low- and middle-income countries. Geneva: World Health Organization; 2012.

For peer review only

For peer review only - http://bmjopen.bmj.com/site/about/guidelines.xhtml

Figure 1. Zero TB Indicator Framework Cascades, adapted for Mongolia.

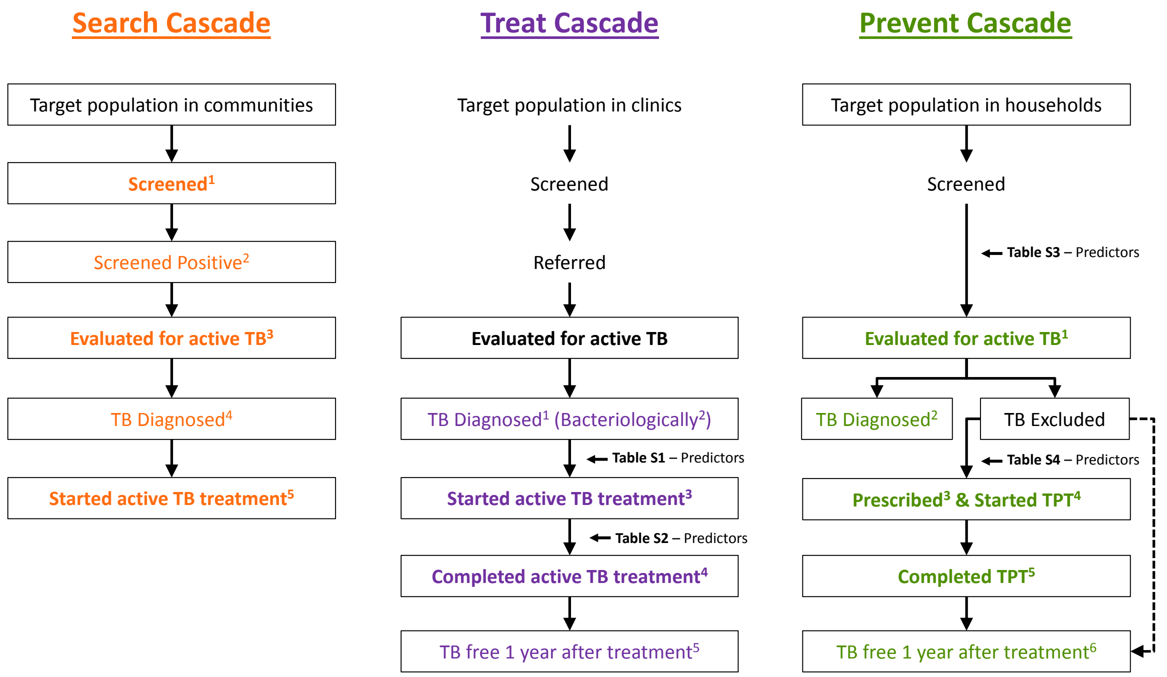

Abbreviations: TB, tuberculosis.

Legend: Flow diagrams showing the movement of individuals through each individual step of the three TB delivery cascades within the Zero TB Indicator Framework. These have been modified to fit data available in Mongolia, and color coded to differentiate among the Search, Treat, and Prevent service cascades. Steps framed within a rectangular box are derived from the Zero TB Indicator Framework, while unframed steps indicate additional important steps within the cascades. Solid arrows show the direction of patient flow through the cascades; a dashed arrow shows where excluded individuals re-enter the cascade. Colored box labels correspond to the 16 Zero TB Framework indicators, with superscripts identifying indicator numbers as defined in Table 1; 5 come from the Search Cascade, 5 from the Treat Cascade, and 6 from the Prevent Cascade. The indicators in **bolded text** describe the quality of care at a given step in the cascade, while the non-bolded indicators describe the diagnostic and/or epidemiological yield of that step in TB care. Finally, brief references to the following supplemental tables are located adjacent to the steps where they will identify barriers to TB care through multivariate models: Table S1, Predictors of Starting Active TB treatment (Treat Cascade); Table S2, Predictors of Completing Active TB Treatment (Treat Cascade); Table S3, Predictors of Competing Evaluation (Prevent Cascade); Table S4, Predictors of Being Prescribed and Initiating TPT (Prevent Cascade).

For peer review only - http://bmjopen.bmj.com/site/about/guidelines.xhtml

Figure 2. Flow diagram showing the Search Cascade as adapted from the Zero TB Indicator Framework for Mongolia.

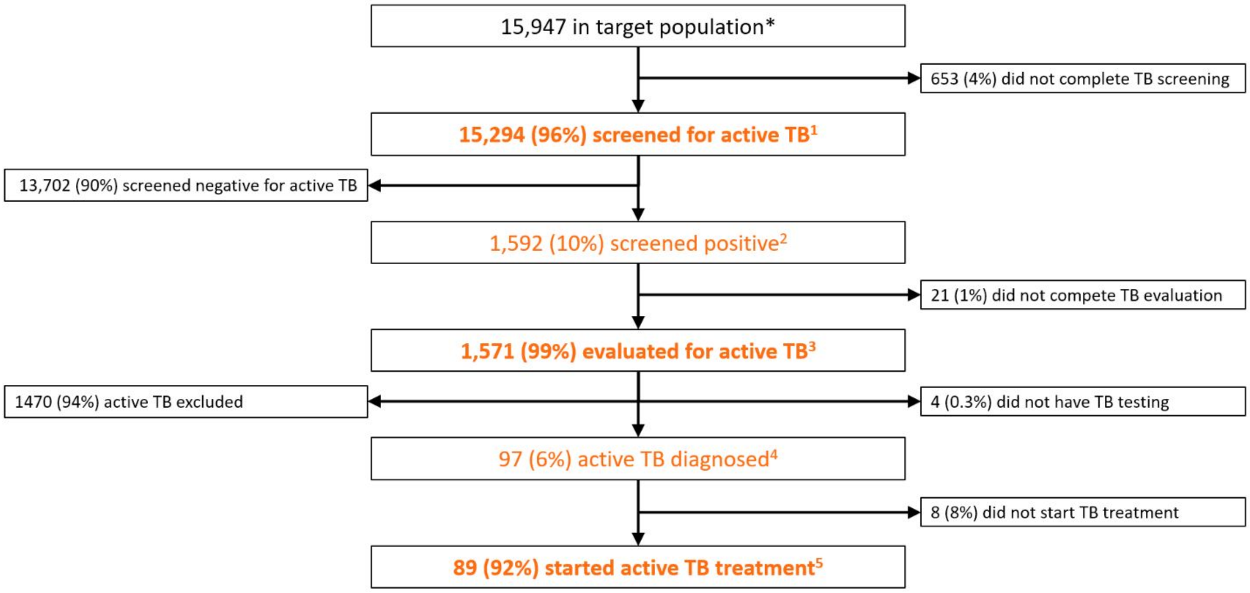

Abbreviations: TB, tuberculosis.

Legend: Flow diagrams showing individuals entering and exiting at each step of the three cascades, either appropriately to the left side after having received guideline-adherent care or inappropriately to the right side after having received guideline-nonadherent care. Zero TB Indicators Treat indicators are shown in orange, with superscripts identifying indicator numbers as defined in Table 1. The indicators in **bolded text** describe the quality of care at a given step in the cascade, while the non-bolded indicators describe the diagnostic and/or epidemiological yield of that step in TB care.

For peer review only - http://bmjopen.bmj.com/site/about/guidelines.xhtml

Figure 3. Flow diagram showing the Treat Cascade as adapted from the Zero TB Indicator Framework for Mongolia

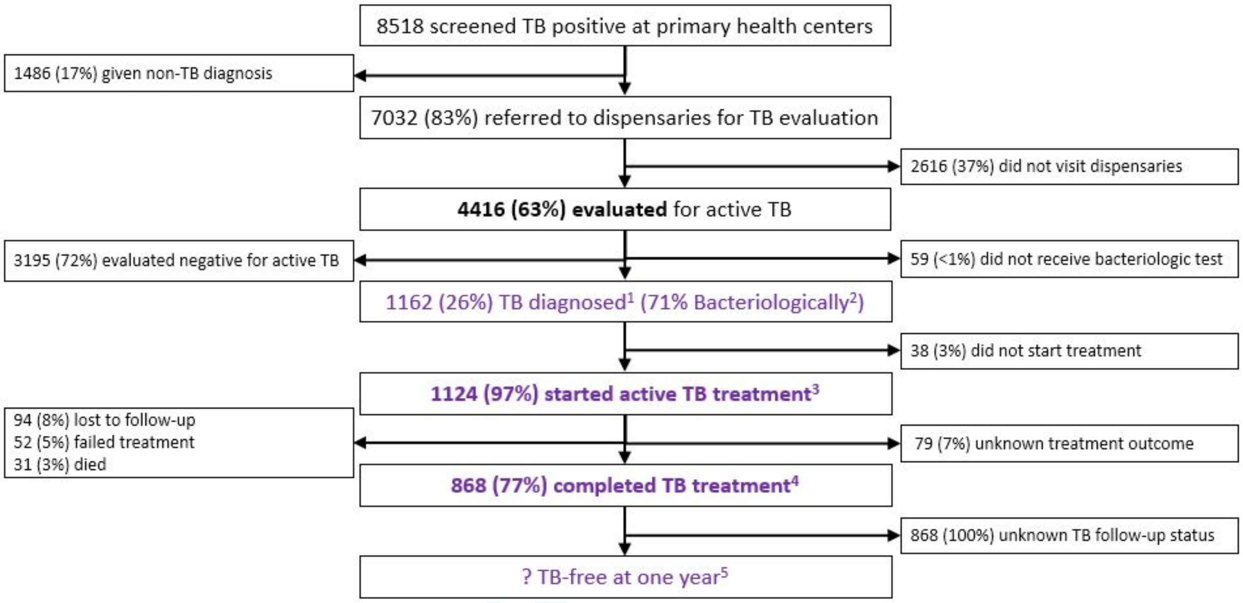

Abbreviations: TB, tuberculosis.
Legend: Flow diagrams showing individuals entering and exiting at each step of the three cascades, either appropriately to the left side after having received guideline-adherent care or inappropriately to the right side after having received guideline-nonadherent care. Zero TB Treat indicators are shown in purple, with superscripts identifying indicator numbers as defined in Table 1. The indicators in **bolded text** describe the quality of care at a given step in the cascade, while the non-bolded indicators describe the diagnostic and/or epidemiological yield of that step in TB care.

For peer review only - http://bmjopen.bmj.com/site/about/guidelines.xhtml

Figure 4. Flow diagram showing the Prevent Cascade, as adapted from the Zero TB Indicator Framework for Mongolia.

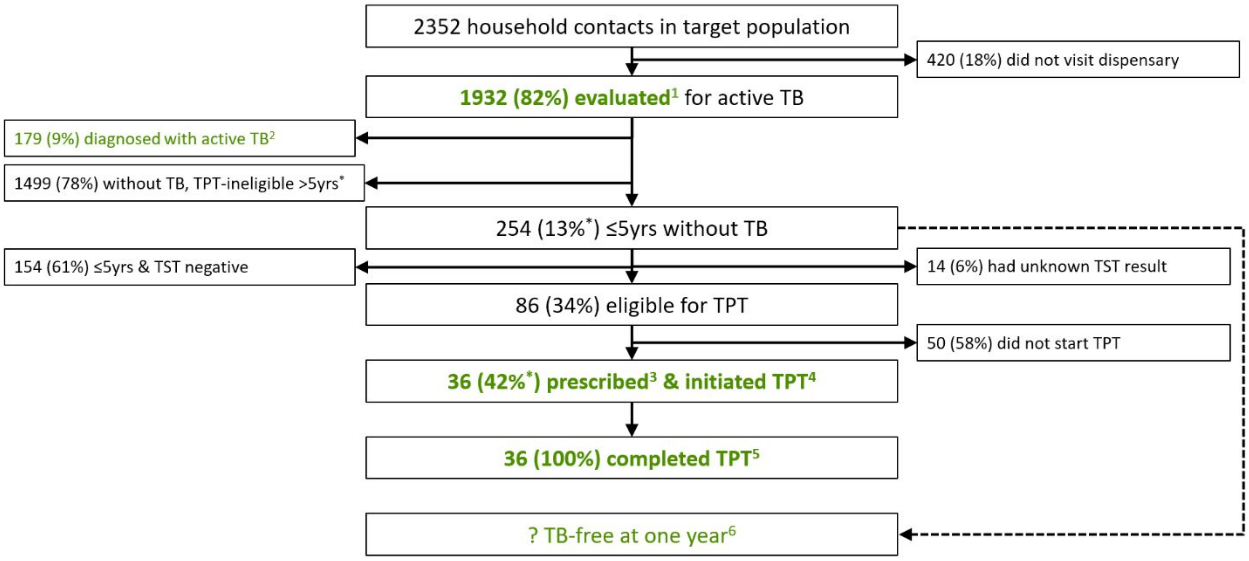

Abbreviations: TB, tuberculosis.

Legend: Flow diagrams showing individuals entering and exiting at each step of the three cascades, either appropriately to the left side after having received guideline-adherent care or inappropriately to the right side after having received guideline-nonadherent care. The adapted Zero TB Treat indicators are shown in green and numbered using superscripts as defined in Table 1. The indicators in **bolded text** describe the quality of care at a given step in the cascade, while the non-bolded indicators describe the diagnostic and/or epidemiological yield of that step in TB care. *Percentages derived from multivariate, logistic regression models using generalizing estimations and may differ from crude proportions. ?, unknown.

For peer review only - http://bmjopen.bmj.com/site/about/guidelines.xhtml

Figure 5. Cumulative proportions of participants receiving high-quality TB care, by delivery cascade

Panel A. Search Cascade (n=15,947)

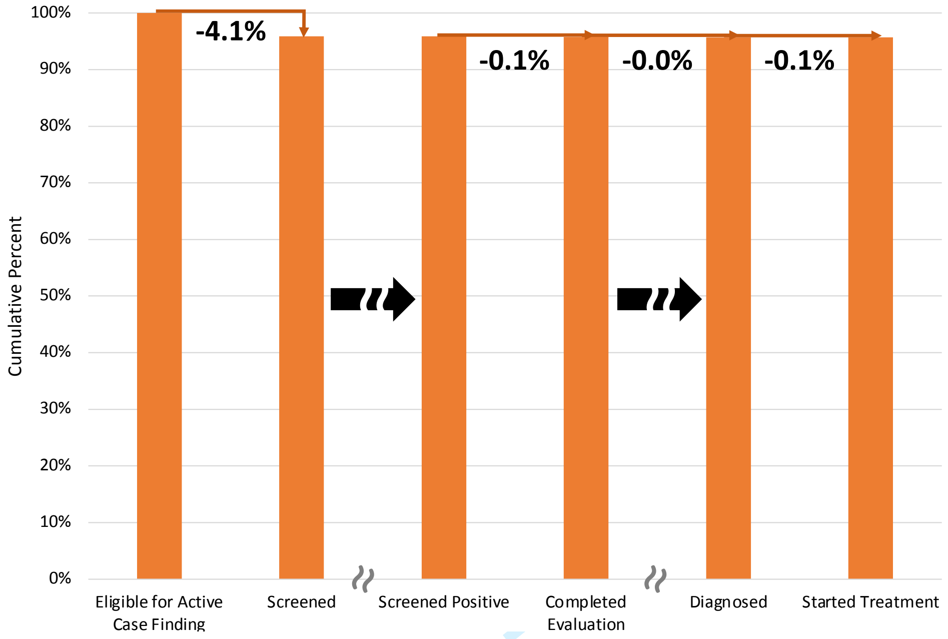

Panel B. Treat Cascade (n=8,518)

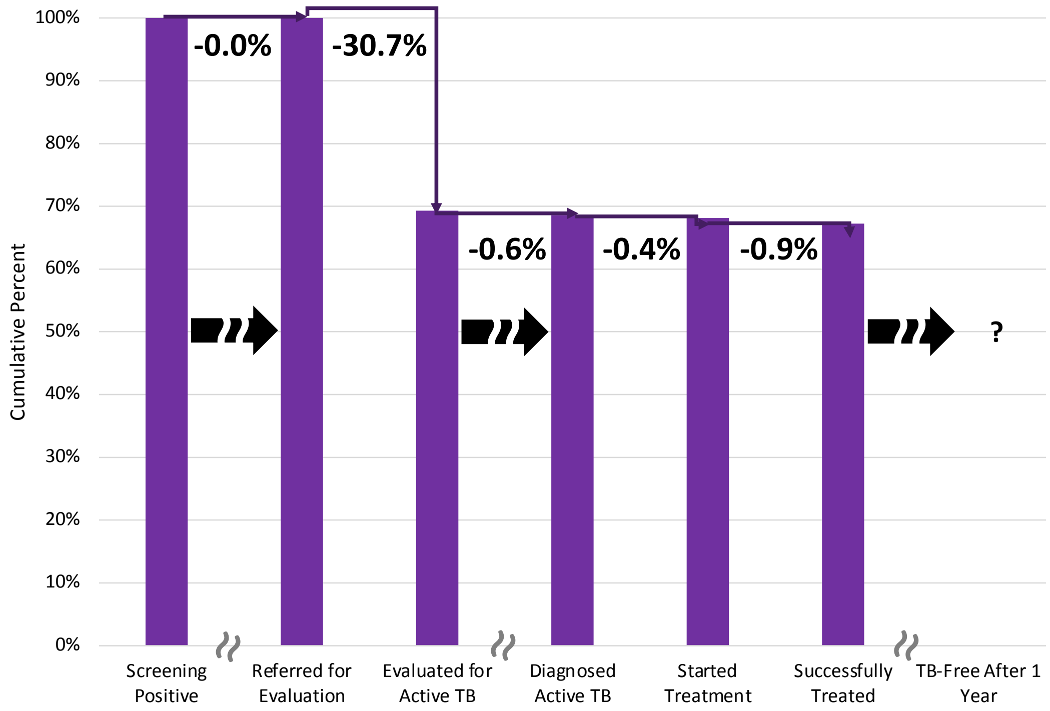

For peer review only - http://bmjopen.bmj.com/site/about/guidelines.xhtml

Figure 5, continued.

Panel C. Prevent Cascade (n=2352)

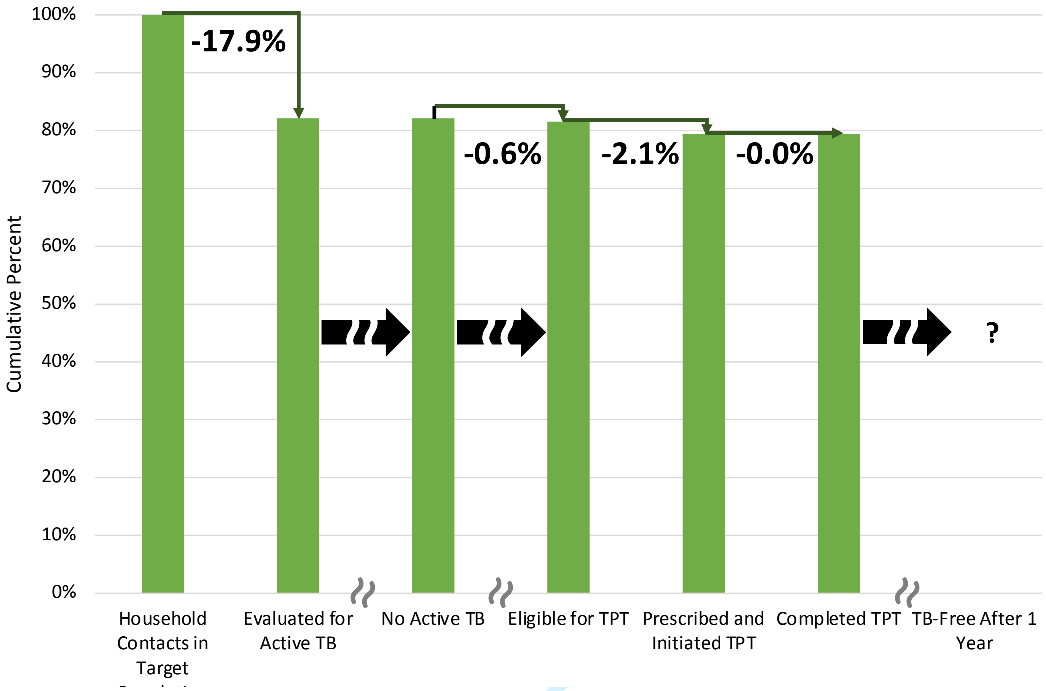

Legend: Bar graphs showing the cumulative probability of completing the key processes with each of the three TB delivery cascades. The probabilities for each step, shown as bars, were calculated as the simple proportion of all individuals receiving guideline-recommended care at the end of that step and all previous steps, divided by the total number entering the cascade. Thin elbow connector lines show the percentage lost with each step, calculated as the simple proportion of individuals not receiving guideline-recommended care at that step divided by the total number entering the cascade. Block arrows with discontinuity lines shows steps where individuals exit the cascade having received all guideline-recommended care.

For peer review only - http://bmjopen.bmj.com/site/about/guidelines.xhtml

ONLINE SUPPLEMENT

Supplemental Methods

Setting

Chingeltei, Bayanzurkh and Khan-Uul are three densely populated districts, among nine total districts in the Mongolian capital, Ulaanbaatar. Chingeltei District has both urban and rural areas and was recognized as a model district at the 2018 National TB Forum. Bayanzurkh and Khan-Uul are over 50% rural. Mandal is the highest-population sub-district in Selenge, and has two prisons and three large mines, both of which are high-risk TB settings, and has established active surveillance and screening programs at high schools. Case notification rates in all four communities approximate the national average case notification rate of 124/100,000 persons.

For peer review only

For peer review only - http://bmjopen.bmj.com/site/about/guidelines.xhtml

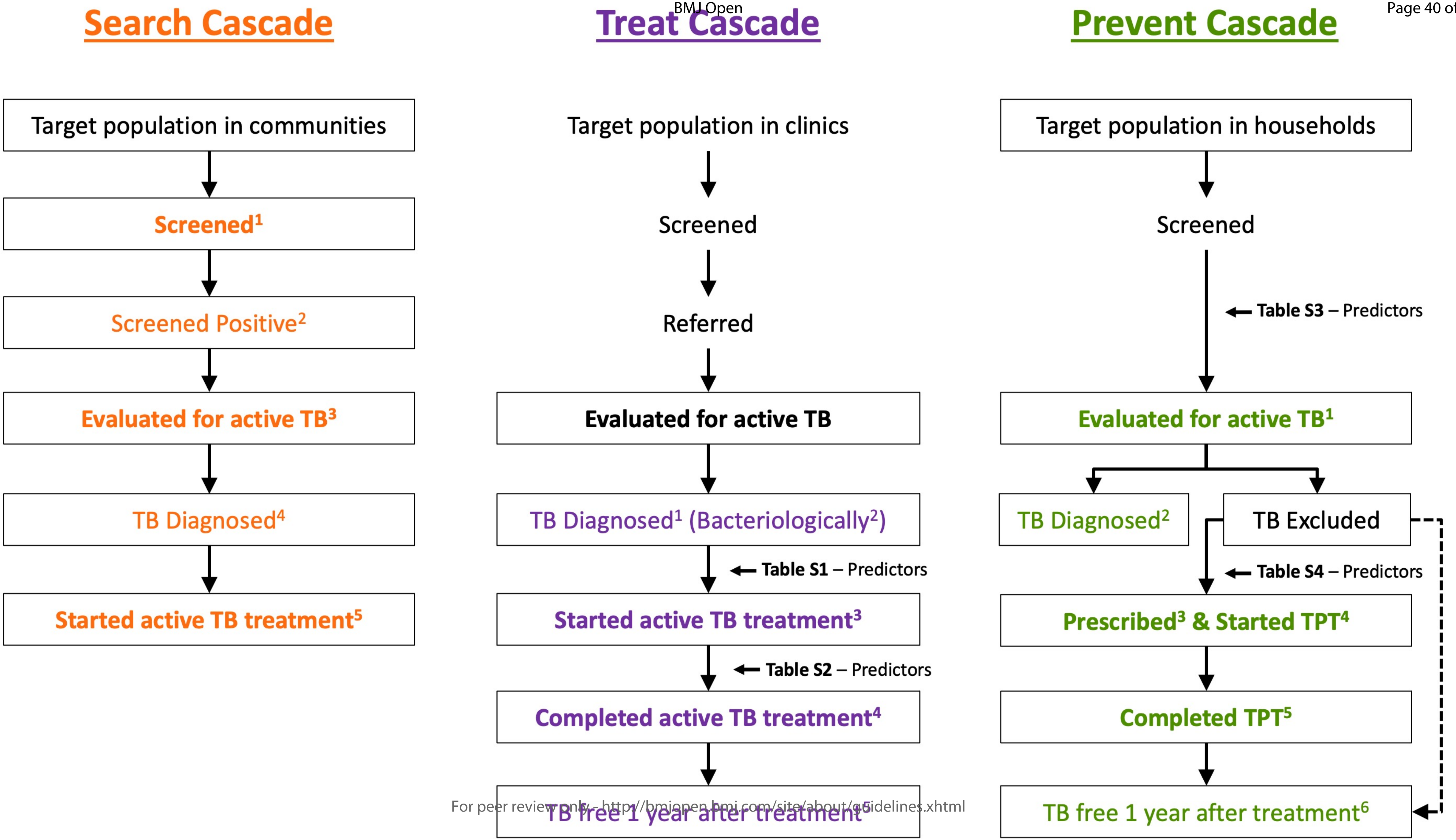
Search Cascade
Treat Cascade
Prevent Cascade
Target population in communities
Screened[1]
Screened Positive[2]
Evaluated for active TB[3]
TB Diagnosed[4]
Started active TB treatment[5]
Target population in clinics
Screened
Referred
Evaluated for active TB
TB Diagnosed[1] (Bacteriologically[2])
Table S1 – Predictors
Started active TB treatment[3]
Table S2 – Predictors
Completed active TB treatment[4]
TB free 1 year after treatment[5]
Target population in households
Screened
Table S3 – Predictors
Evaluated for active TB[1]
TB Diagnosed[2]
TB Excluded
Table S4 – Predictors
Prescribed[3] & Started TPT[4]
Completed TPT[5]
TB free 1 year after treatment[6]

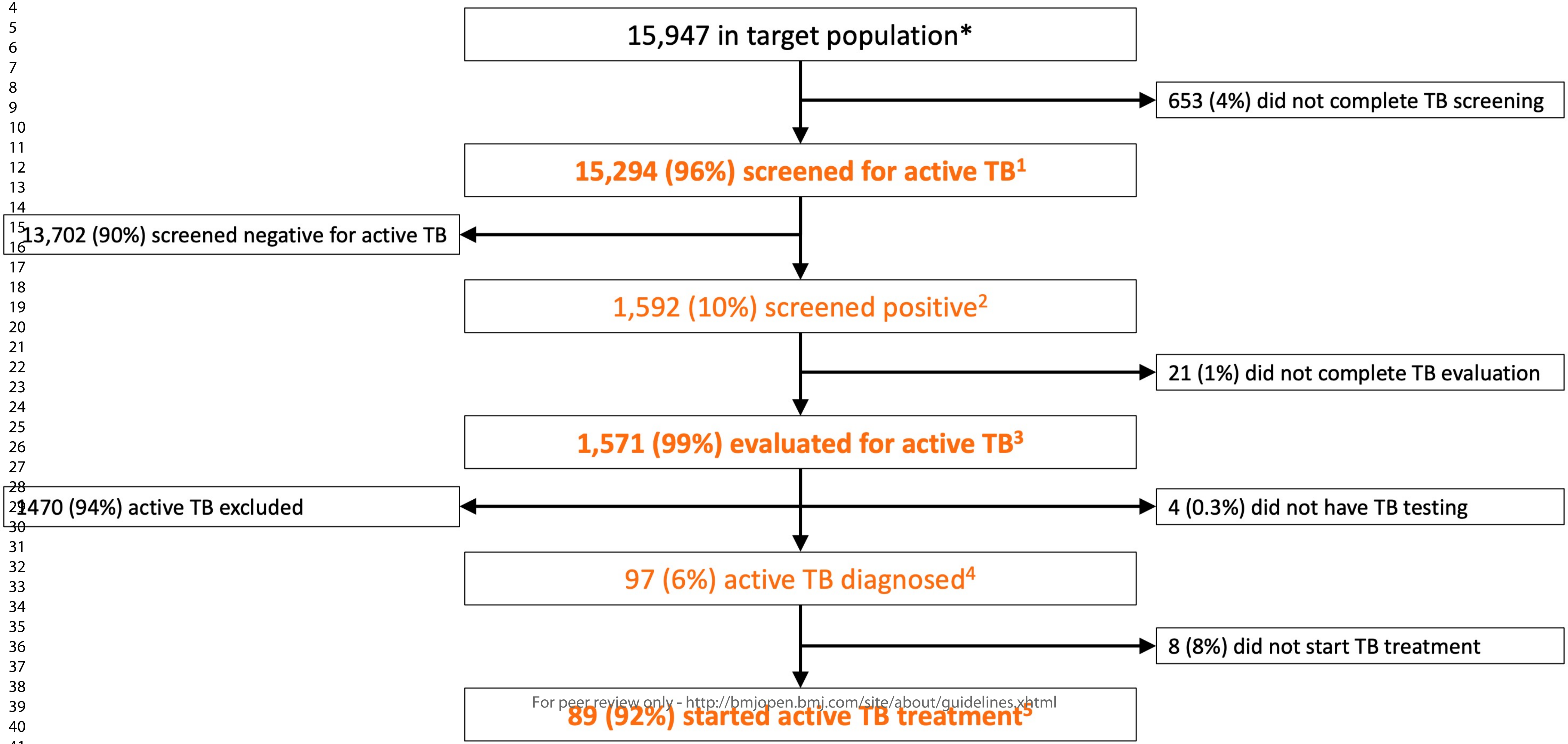

For peer review only - http://bmjopen.bmj.com/site/about/guidelines.xhtml

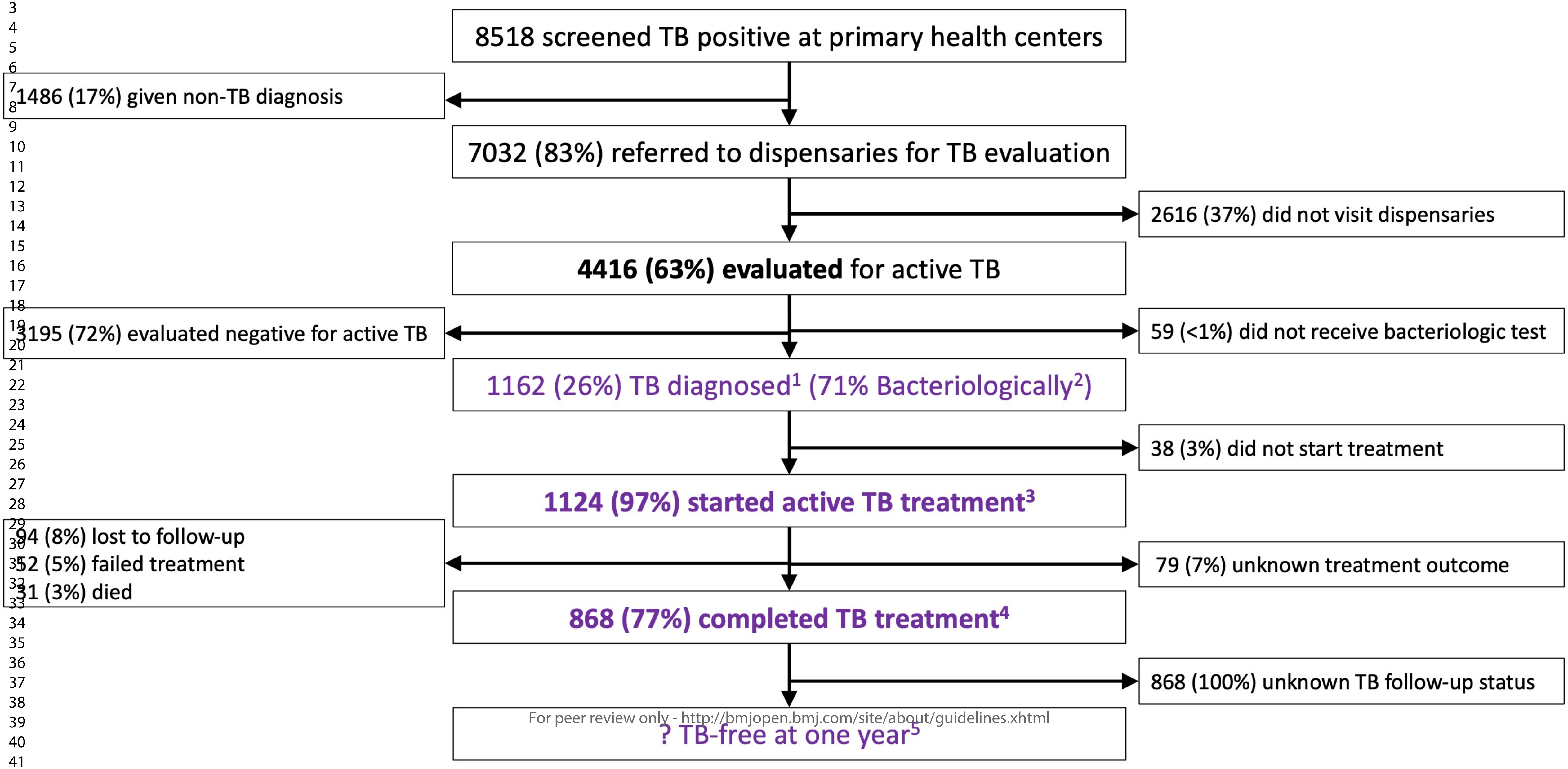

For peer review only - http://bmjopen.bmj.com/site/about/guidelines.xhtml

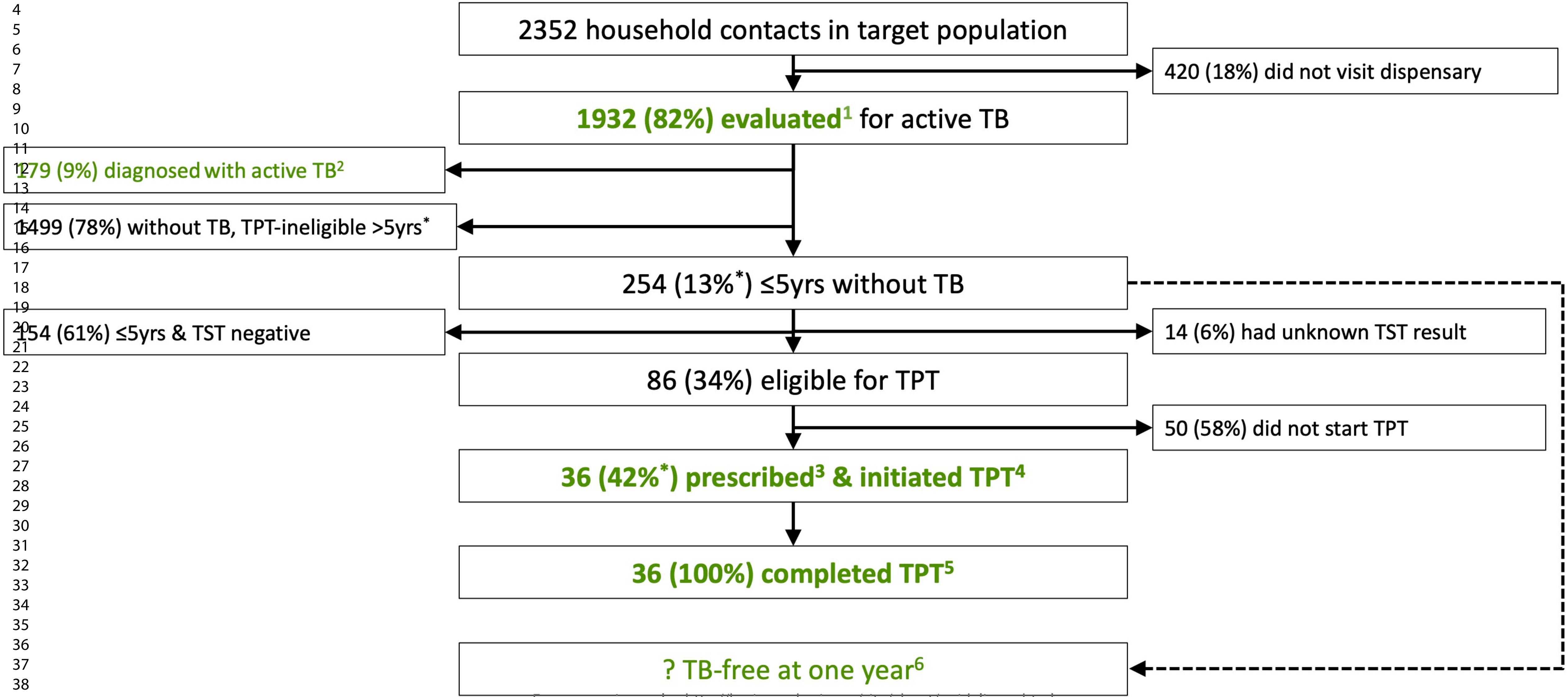

For peer review only - http://bmjopen.bmj.com/site/about/guidelines.xhtml

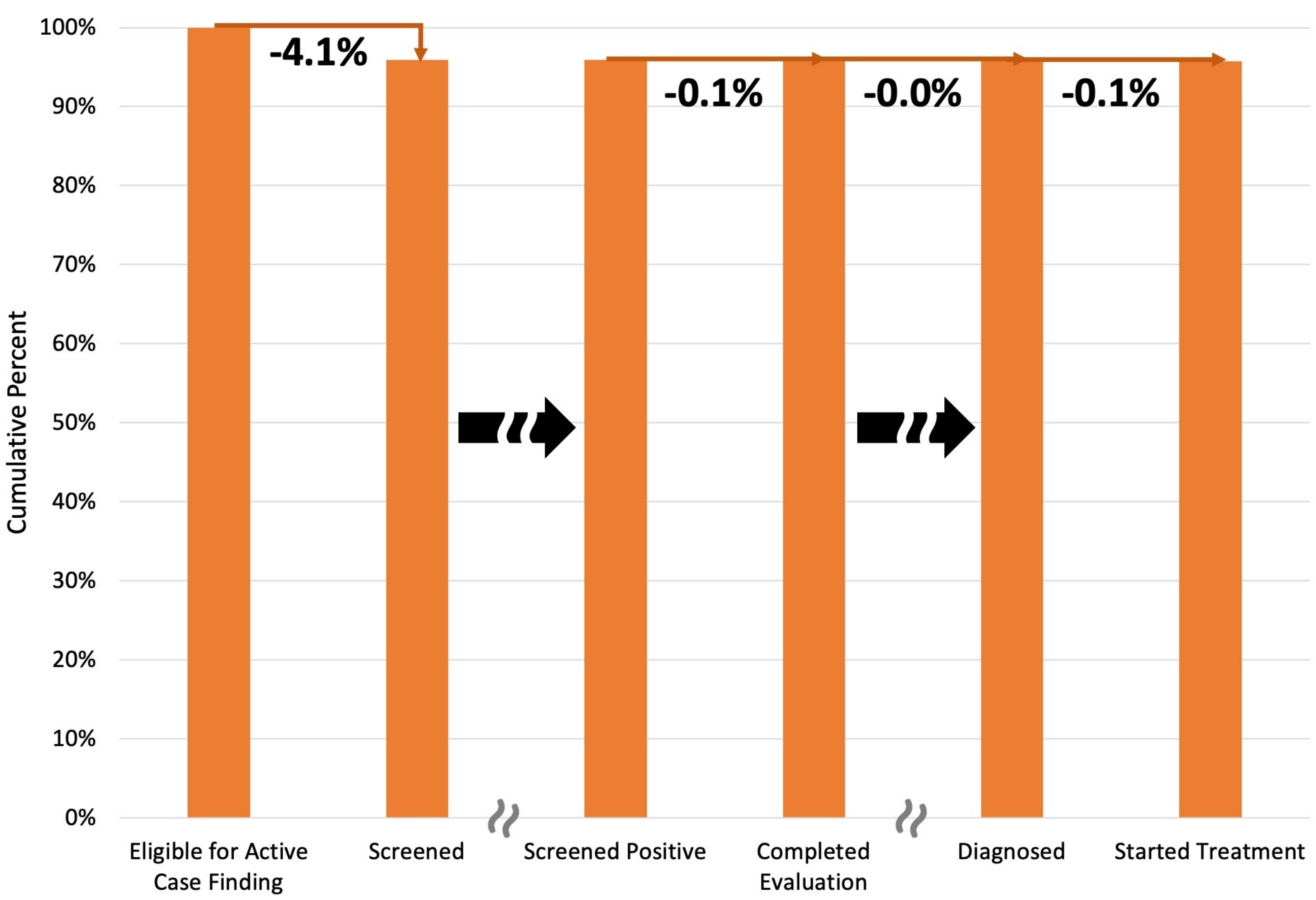
100%
90%
80%
70%
60%
50%
40%
30%
20%
10%
0%
Cumulative Percent
-4.1%
-0.1%
-0.0%
-0.1%
Eligible for Active Case Finding
Screened
Screened Positive
Completed Evaluation
Diagnosed
Started Treatment

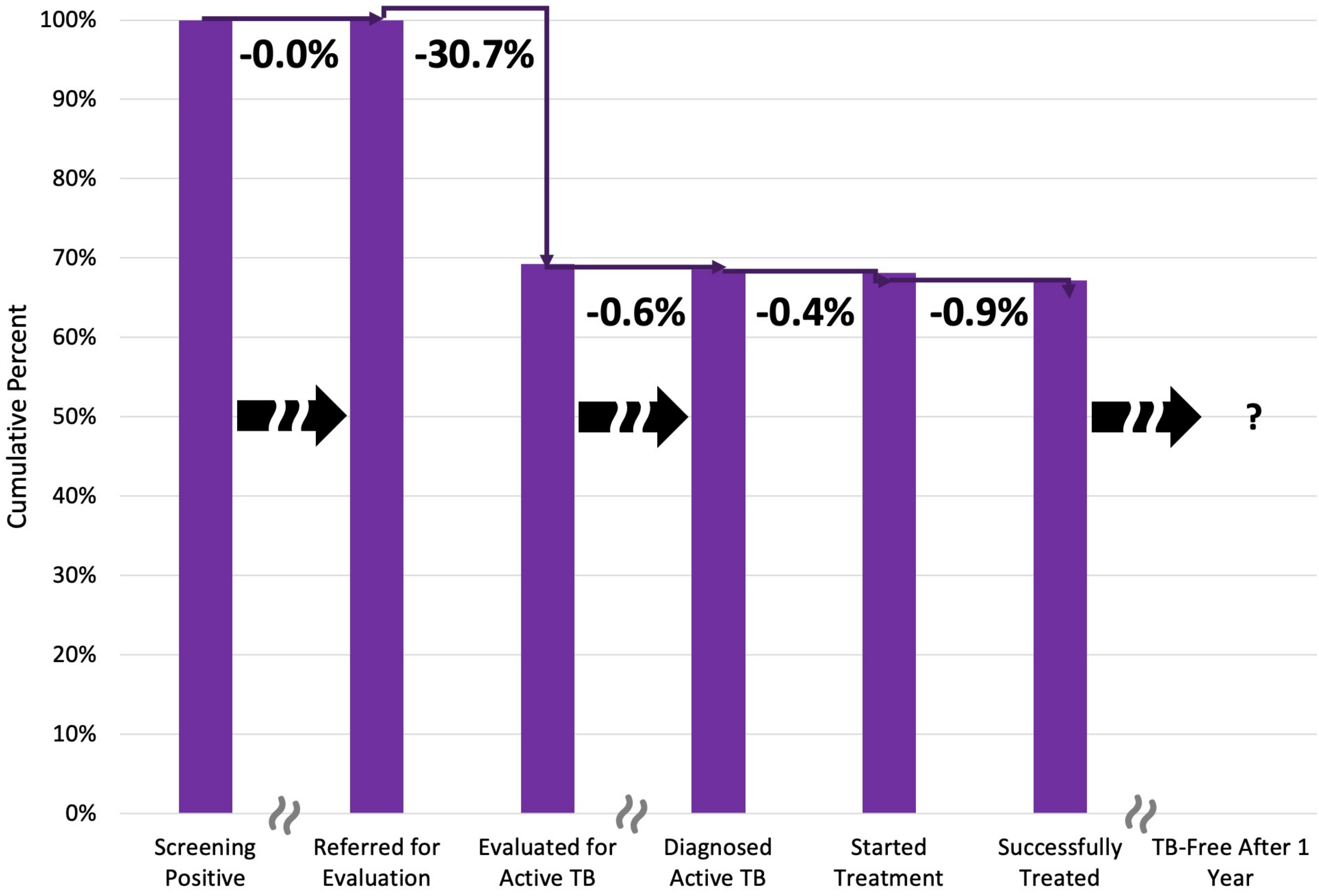
100%
90%
80%
70%
60%
50%
40%
30%
20%
10%
0%
Cumulative Percent
-0.0%
-30.7%
-0.6%
-0.4%
-0.9%
?
Screening Positive
Referred for Evaluation
Evaluated for Active TB
Diagnosed Active TB
Started Treatment
Successfully Treated
TB-Free After 1 Year

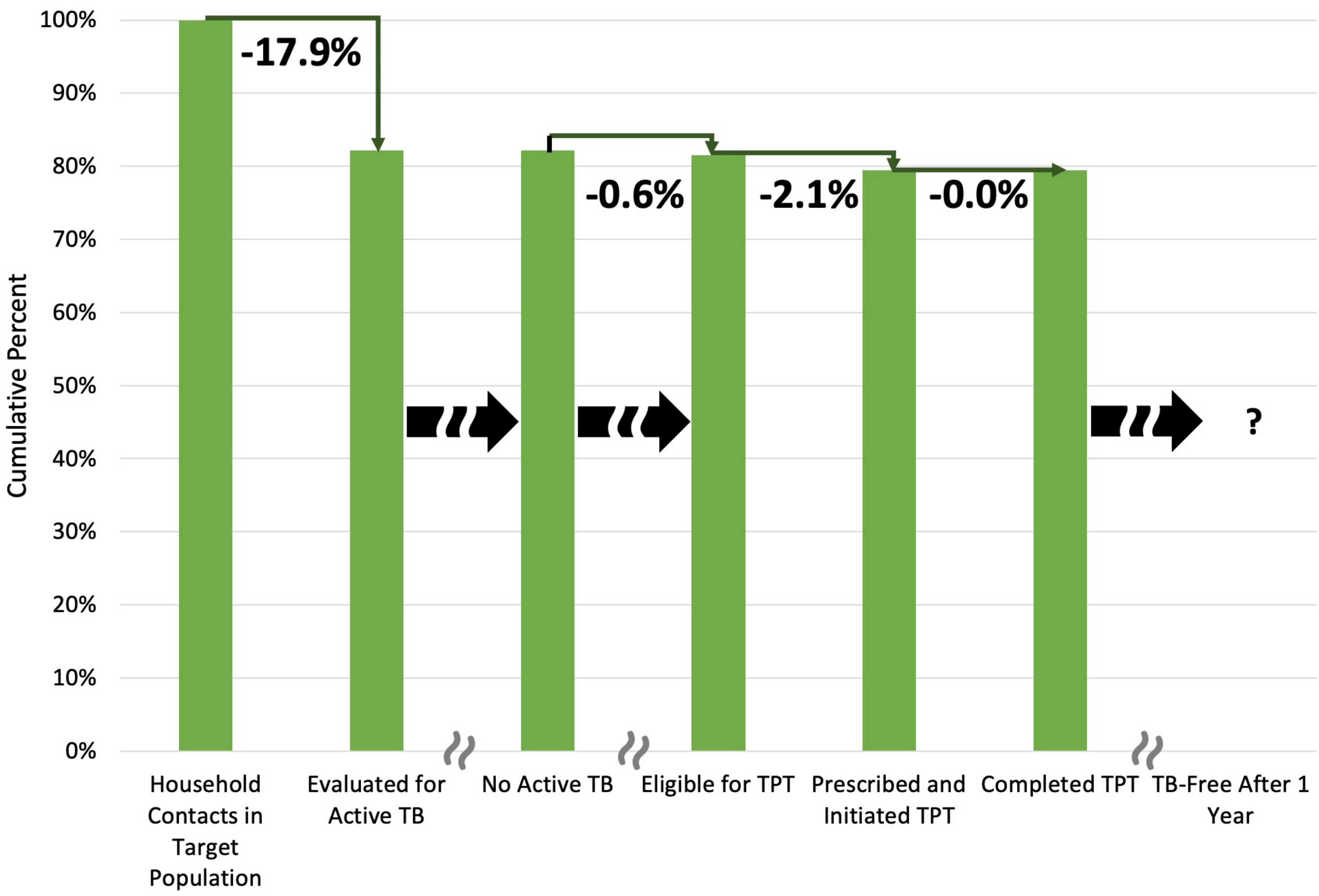
100%
90%
80%
70%
60%
50%
40%
30%
20%
10%
0%
Cumulative Percent
-17.9%
-0.6%
-2.1%
-0.0%
?
Household Contacts in Target Population
Evaluated for Active TB
No Active TB
Eligible for TPT
Prescribed and Initiated TPT
Completed TPT
TB-Free After 1 Year

SUPPLEMENTAL FIGURES AND TABLES

Table S0. Treat cascade indicators and cumulative probability of receiving high-quality TB diagnosis and treatment, stratified by study site.

Indicator	Bayanzurkh	Khan-Uul	Chingeltei	Mandal	p-value
Screened Positive	n=3,245	n=2,620	n=2,160	n=493	
Referred	2,867 (88%)	2,217 (85%)	1,455 (67%)	493 (100%)	<0.001
Evaluated	1,349 (47%)	1,372 (62%)	1,208 (83%)	487 (99%)	<0.001
Diagnosed[1]	595 (44%)	249 (18%)	276 (23%)	42 (9%)	<0.001
Started Treatment[3]	577 (97%)	237 (95%)	271 (98%)	39 (93%)	0.090
Completed Treatment[4]	416 (72%)	202 (85%)	216 (80%)	34 (87%)	<0.001
Cumulatively Diagnosed & Treated	29%	42%	44%	80%	<0.001

Legend: The Zero TB Treat indicators are numbered using superscripts as defined in Table 1. Shaded rows indicate steps whose yield is determined by the quality of care, while non-shaded rows indicate steps whose yield is determined by epidemiology and the sensitivity of screen and diagnostic tools.

Table S1. Predictors of Starting Active TB Treatment (Treatment Cascade)

Characteristic (n=1162)	aOR	95% CI	p-Value
Age (per decade)	0.72	0.66-0.79	<0.001
Male gender	0.73	0.62-0.86	<0.001

Abbreviations: aOR, adjusted odds ratio. 95% CI, 95% Confidence Intervals.

Table S2. Predictors of Completing Active TB Treatment (Treatment Cascade)

Characteristic (n=1124)	aOR	95% CI	p-Value
Age (per decade)	0.76	0.71-0.83	<0.001
Male gender	0.56	0.36-0.88	0.013

Abbreviations: aOR, adjusted odds ratio. 95% CI, 95% Confidence Intervals.

For peer review only

Table S3. Predictors of Completing TB Evaluation (Prevent Cascade)

Characteristic (n=2352)	aOR	95% CI	p-Value
Age of contact (per decade)	0.92	0.86-0.98	0.015
Male gender of contact	0.90	0.73-1.12	0.35
Immediate family member of index patient	0.92	0.71-1.17	0.48
Age of index patient (per decade)	1.01	0.93-1.10	0.81
Male gender of index patient	0.89	0.64-1.24	0.47

Abbreviations: aOR, adjusted odds ratio. 95% CI, 95% Confidence Intervals.

Table S4. Predictors of Being Prescribed and Initiating TPT (Prevent Cascade)

Characteristic (n=86)	aOR	95% CI	p-Value
Age of contact (per year)	0.76	0.55-1.06	0.11
Male gender of contact	0.78	0.22-2.82	0.71
Immediate family member of index patient	0.46	0.10-1.99	0.30
Age of index patient (per decade)	0.60	0.38-0.93	0.022
Male gender of index patient	0.97	0.37-2.57	0.95

Abbreviations: aOR, adjusted odds ratio. 95% CI, 95% Confidence Intervals.

For peer review only

Appendix S1.

Search Strategy

We search all articles in PubMed in all language published between January 1, 2000, and September 3, 2021, using the following strategy:

1. Search terms for tuberculosis

"tuberculosis"[Mesh] OR *Mycobacterium tuberculosis*[tiab] OR TB[tiab]

2. Search terms for cascade analysis

cascade[tiab] OR cascades[tiab] OR continuum[tiab] OR continua[tiab] or Process Assessment, Health Care[MESH]

3. Search terms for low- and middle-income countries

(Deprived Countries[tw] OR Deprived Population[tw] OR Deprived Populations[tw] OR Developing Countries[tw] OR Developing Country[tw] OR Developing Economies[tw] OR Developing Economy[tw] OR Developing Nation[tw] OR Developing Nations[tw] OR Developing Population[tw] OR Developing Populations[tw] OR Developing World[tw] OR LAMI Countries[tw] OR LAMI Country[tw] OR Less Developed Countries[tw] OR Less Developed Country[tw] OR Less Developed Economies [tw] OR Less Developed Nation[tw] OR Less Developed Nations[tw] OR Less Developed World[tw] OR Lesser Developed Countries[tw] OR Lesser Developed Nations[tw] OR LMIC[tw] OR LMICS[tw] OR Low GDP[tw] OR Low GNP[tw] OR Low Gross Domestic[tw] OR Low Gross National[tw] OR Low Income Countries[tw] OR Low Income Country[tw] OR Low Income Economies [tw] OR Low Income Economy[tw] OR Low Income Nations[tw] OR Low Income Population[tw] OR Low Income Populations[tw] OR Lower GDP[tw] OR lower gross domestic[tw] OR Lower Income Countries[tw] OR Lower Income Country[tw] OR Lower Income Nations[tw] OR Lower Income Population[tw] OR Lower Income Populations[tw] OR Middle Income Countries[tw] OR Middle Income Country[tw] OR Middle Income Economies [tw] OR Middle Income Nation[tw] OR Middle Income Nations[tw] OR Middle Income Population[tw] OR Middle Income Populations[tw] OR Poor Countries[tw] OR Poor Country[tw] OR Poor Economies [tw] OR Poor Economy[tw] OR Poor Nation[tw] OR Poor Nations[tw] OR Poor Population[tw] OR Poor Populations[tw] OR poor world[tw] OR Poorer Countries[tw] OR Poorer Economies [tw] OR Poorer Economy[tw] OR Poorer Nations[tw] OR Poorer Population[tw] OR Poorer Populations[tw] OR Third World[tw] OR Transitional Countries[tw] OR Transitional Country[tw] OR Transitional Economies[tw] OR Transitional Economy[tw] OR Under Developed Countries[tw] OR Under Developed Country[tw] OR under developed nations[tw] OR Under Developed World[tw] OR Under Served Population[tw] OR Under Served Populations[tw] OR Underdeveloped Countries[tw] OR Underdeveloped Country[tw] OR underdeveloped economies[tw] OR underdeveloped nations[tw] OR underdeveloped population[tw] OR Underdeveloped World[tw] OR Underserved Countries[tw] OR Underserved Nations[tw] OR Underserved Population[tw] OR Underserved Populations[tw])

For peer review only

The 23 studies that we identified were:

1. Davis JL, Katamba A, Vasquez J, Crawford E, Sserwanga A, Kakeeto S, Kizito F, Dorsey G, den Boon S, Vittinghoff E, Huang L, Adatu F, Kamya MR, Hopewell PC, Cattamanchi A. Evaluating tuberculosis case detection via real-time monitoring of tuberculosis diagnostic services. Am J Respir Crit Care Med. 2011;184(3):362-7. PMID: 21471088; PMCID: 3175538.

2. Davis JL, Dowdy DW, den Boon S, Walter ND, Katamba A, Cattamanchi A. Test and treat: a new standard for smear-positive tuberculosis. J Acquir Immune Defic Syndr. 2012;61(1):e6-e8. PMID: 22918128; PMCID: 3427531.

3. Marquez C, Davis JL, Katamba A, Haguma P, Ochom E, Ayakaka I, Chamie G, Dorsey G, Kamya MR, Charlebois E, Havlir DV, Cattamanchi A. Assessing the quality of tuberculosis evaluation for children with prolonged cough presenting to routine community health care settings in rural Uganda. PLoS One. 2014;9(8):e105935. PMID: 25170875; PMCID: 4149493.

4. Sun AY, Denkinger CM, Dowdy DW. The impact of novel tests for tuberculosis depends on the diagnostic cascade. Eur Respir J. 2014;44(5):1366-9. PMID: 25186263; PMCID: PMC4254765.

5. Chaisson LH, Katamba A, Haguma P, Ochom E, Ayakaka I, Mugabe F, Miller C, Vittinghoff E, Davis JL, Handley MA, Cattamanchi A. Theory-informed interventions to improve the quality of tuberculosis evaluation at Ugandan health centers: a quasi-experimental study. PLoS ONE. 2015;10(7):e0132573; PMCID: 4501843.

6. Alsdurf H, Hill PC, Matteelli A, Getahun H, Menzies D. The cascade of care in diagnosis and treatment of latent tuberculosis infection: a systematic review and meta-analysis. Lancet Infect Dis. 2016;16(11):1269-78. PMID: 27522233.

7. Daftary A, Jha N, Pai M. Enhancing the role of pharmacists in the cascade of tuberculosis care. Journal of Epidemiology and Global Health. 2016;7(1):1.

8. Subbaraman R, Nathavitharana RR, Satyanarayana S, Pai M, Thomas BE, Chadha VK, Rade K, Swaminathan S, Mayer KH. The Tuberculosis Cascade of Care in India's Public Sector: A Systematic Review and Meta-analysis. PLOS Medicine. 2016;13(10):e1002149.

9. Armstrong-Hough M, Turimumahoro P, Meyer AJ, Ochom E, Babirye D, Ayakaka I, Mark D, Ggita J, Cattamanchi A, Dowdy D, Mugabe F, Fair E, Haberer JE, Katamba A, Davis JL. Drop-out from the tuberculosis contact investigation cascade in a routine public health setting in urban Uganda: A prospective, multi-center study. PLoS One. 2017;12(11):e0187145. PMID: 29108007; PMCID: PMC5673209.

10. Cazabon D, Alsdurf H, Satyanarayana S, Nathavitharana R, Subbaraman R, Daftary A, Pai M. Quality of tuberculosis care in high burden countries: the urgent need to address gaps in the care cascade. Int J Infect Dis. 2017;56:111-6. PMID: 27794468; PMCID: PMC5346036.

11. Hanrahan CF, Van Rie A. A proposed novel framework for monitoring and evaluation of the cascade of HIV-associated TB care at the health facility level. Journal of the International AIDS Society. 2017;20(1):21375.

For peer review only

12. Mwangwa F, Chamie G, Kwarisiima D, Ayieko J, Owaraganise A, Ruel TD, Plenty A, Tram KH, Clark TD, Cohen CR, Bukusi EA, Petersen M, Kamya MR, Charlebois ED, Havlir DV, Marquez C. Gaps in the Child Tuberculosis Care Cascade in 32 Rural Communities in Uganda and Kenya. J Clin Tuberc Other Mycobact Dis. 2017;9:24-9. PMID: 29291251; PMCID: PMC5743212.

13. Naidoo P, Theron G, Rangaka MX, Chihota VN, Vaughan L, Brey ZO, Pillay Y. The South African Tuberculosis Care Cascade: Estimated Losses and Methodological Challenges. J Infect Dis. 2017;216(suppl_7):S702-s13. PMID: 29117342; PMCID: PMC5853316.

14. Yuen C, Becerra M, Codlin A, Creswell J, Ditiu L, Keshavjee S, Khan A, Nicholson T, Page-Shipp L, Sahu S, Wilson M. A best-practice framework of program indicators for monitoring a comprehensive approach to the tuberculosis epidemic. Zero TB Initiative, 2017.

15. Shapiro AE, van Heerden A, Schaafsma TT, Hughes JP, Baeten JM, van Rooyen H, Tumwesigye E, Celum CL, Barnabas RV. Completion of the tuberculosis care cascade in a community-based HIV linkage-to-care study in South Africa and Uganda. J Int AIDS Soc. 2018;21(1). PMID: 29381257; PMCID: PMC5810338.

16. Agins BD, Ikeda DJ, Reid MJA, Goosby E, Pai M, Cattamanchi A. Improving the cascade of global tuberculosis care: moving from the “what” to the “how” of quality improvement. The Lancet Infectious Diseases. 2019;19(12):e437-e43.

17. Arsenault C, Roder-DeWan S, Kruk ME. Measuring and improving the quality of tuberculosis care: A framework and implications from the Lancet Global Health Commission. J Clin Tuberc Other Mycobact Dis. 2019;16:100112. PMID: 31497655; PMCID: PMC6716550.

18. Kim J, Keshavjee S, Atun R. Health systems performance in managing tuberculosis: analysis of tuberculosis care cascades among high-burden and non-high-burden countries. J Glob Health. 2019;9(1):010423. PMID: 31263546; PMCID: PMC6592589 www.icmje.org/coi_disclosure.pdf (available upon request from the corresponding author), and declare no conflicts of interest.

19. Subbaraman R, Nathavitharana RR, Mayer KH, Satyanarayana S, Chadha VK, Arinaminpathy N, Pai M. Constructing care cascades for active tuberculosis: A strategy for program monitoring and identifying gaps in quality of care. PLoS Med. 2019;16(2):e1002754. PMID: 30811385; PMCID: PMC6392267.

20. Der JB, Grint D, Narh CT, Bonsu F, Grant AD. Where are patients missed in the tuberculosis diagnostic cascade? A prospective cohort study in Ghana. PLoS One. 2020;15(3):e0230604. PMID: 32191768; PMCID: PMC7081980.

21. Diaz G, Victoria AM, Meyer AJ, Nino Y, Luna L, Ferro BE, Davis JL. Evaluating the Quality of Tuberculosis Contact Investigation in Cali, Colombia: A Retrospective Cohort Study. Am J Trop Med Hyg. 2021. PMID: 33617470; PMCID: PMC8045602.

22. van de Water B, Meyer T, Wilson M, Young C, Gaunt B, le Roux K. TB prevention cascade at a district hospital in rural Eastern Cape, South Africa. Public Health Action. 2021;11(2):97-100.

For peer review only

23. Yasobant S, Bhavsar P, Kalpana P, Memon F, Trivedi P, Saxena D. Contributing Factors in the Tuberculosis Care Cascade in India: A Systematic Literature Review. Risk Management and Healthcare Policy. 2021;Volume 14:3275-86.

Findings of the systematic review

We searched PubMed in all languages using a systematic search strategy to identify all articles published between January 1, 2000, and September 3, 2021 including compound search terms related to tuberculosis ("tuberculosis"[Mesh] OR *Mycobacterium tuberculosis*[tiab] OR TB[tiab]); cascades, continua of care, or process evaluations in health care (cascade[tiab] OR cascades[tiab] OR continuum[tiab] OR continua[tiab] or Process Assessment, Health Care[MESH]); and low- and middle-income countries using a complex search strategy (Appendix S3). We screened titles for articles related to TB case-finding, treatment, and/or prevention services, and augmented our search by reviewing citations from retrieved articles to identify additional studies. We identified 22 articles and one monograph from the grey literature, all published between 2011 and 2021 and employing a wide variety of approaches to constructing TB care cascades. Sixteen articles focused on clinic-based TB case finding and treatment, two on TB contact investigation, three on TB preventive therapy, and three on case-finding, prevention, and treatment in combination. Two studies focused on children, while the remaining studies included adults only or adults and children. Eleven studies constructed cascades using primary patient data; four used parameters derived from systematic reviews; three studies used focused literature reviews to parameterize mathematical models; three mentioned the cascade of care as a concept in a review or editorial but did not construct their own cascades; and two proposed integrated frameworks for assessing the quality of case-finding, treatment, and prevention, including one focused on persons living with HIV. Two of the studies constructed cascades by integrating two of these approaches. Only two studies employed cascades as a quality improvement strategy, with both providing real-time performance feedback. Among the 11 studies that constructed cascades from patient data, only two did so using routine surveillance data, and both of these relied on aggregated

For peer review only

national data rather than on individual patient data. The remaining nine studies used subnational data collected by research staff using research databases. We did not identify any studies that collected individual-patient data from routine public health databases.

For peer review only

Appendix S2. Derivation of the variance and confidence interval for estimated cumulative probability of being diagnosed & treated

Let p_1 be the probability of being referred among subjects who were screened positive, p_2 be the probability of being evaluated among subjects who were referred, p_3 be the probability of starting treatment among subjects who were diagnosed TB positive, and p_4 be the probability of complete treatment among subjects who started treatment. Then the cumulative probability of being properly diagnosed and treated for TB given a positive TB diagnosis $p = p_1 p_2\, p_3 p_4$.

Denote n the number of subjects who were screened positive, n_e the number of subjects who were evaluated, n_d the number of subjects who were diagnosed TB positive, and n_c the number of subjects who completed the treatment. The estimated p is

$$\hat{p} = \frac{n_e}{n}\frac{n_c}{n_d},$$

where $\frac{n_e}{n}$ is an estimator of $p_1 p_2$ and $\frac{n_c}{n_d}$ is an estimator of $p_3 p_4$.

We derive the variance of $\log(\hat{p}) = \log\left(\frac{n_e}{n}\right) + \log\left(\frac{n_c}{n_d}\right)$. Suppose the two probabilities $\frac{n_e}{n}$ and $\frac{n_c}{n_d}$ are independent. This is reasonable since the evaluation process and completion of treatment are different.

$$\widehat{Var}[\log(\hat{p})] = \widehat{Var}\left[\log\left(\frac{n_e}{n}\right)\right] + \widehat{Var}\left[\log\left(\frac{n_c}{n_d}\right)\right] = \left(\frac{n_e}{n}\right)^{-2}\widehat{Var}\left(\frac{n_e}{n}\right) + \left(\frac{n_c}{n_d}\right)^{-2}\widehat{Var}\left(\frac{n_c}{n_d}\right)$$
$$= \frac{n - n_e}{n n_e} + \frac{n_d - n_c}{n_d n_c}.$$

The first equality is due to independence of probabilities, the second is due to the delta method, and the last one is simple algebra by substituting the variance of probabilities. The variance of $\hat{p}$ is then by the delta method again,

$$\widehat{Var}[\hat{p}] = \hat{p}^2 * \widehat{Var}[\log(\hat{p})],$$

and the 95% CI is

$$\left(\hat{p} * \exp\left(-1.96 * \widehat{Var}[\log(\hat{p})]\right),\quad \hat{p} * \exp\left(1.96 * \widehat{Var}[\log(\hat{p})]\right)\right).$$

For peer review only

Appendix S3. Rationale for Zero TB Indicator targets provided in Table 1.

Indicator* (Proportion)	Target/ Source	Rationale
**SEARCH CASCADE**		
1. (Screening) Coverage	100%[1]	WHO guidelines state that all patients at risk for TB should be screened.
2. Positive TB symptom screen or chest radiography	25-50%[1]	Systematic reviews undertaken to inform the WHO guidelines examined the WHO 4-symptom screen in the general population and found a positivity rate for symptom screening of approximately 36%, with the 95% Confidence Interval (CI) ranging from 25 to 48%. Concurrent reviews of chest radiography found a lower positivity rate of approximately 11%, with the 95% CI ranging from 8 to 15%. Since symptom screening is used in concert with chest radiography, we have based the target proportion on symptom screening. Substantially higher or lower proportions may suggest the need for quality assurance procedures to ensure that screeners are asking the questions appropriately.
3. Diagnostic evaluation	100%[1]	WHO guidelines state that all patients who are symptom-positive should be evaluated for TB.
4. (Active) TB Diagnosis	10-20%[2]	The Stop TB Partnership sputum smear microscopy guidelines state that the typical smear positivity rate is 10-20%.† Higher or lower proportions suggest the need for quality assurance procedures to ensure that screening and testing are being carried out appropriately.
5. Linkage to TB Treatment	100%[3]	WHO guidelines state that all patients are diagnosed with active TB should be linked to TB treatment.
**TREAT CASCADE**		
1. (Active) TB Diagnosis	10-20%[2]	The Stop TB Partnership sputum smear microscopy guidelines state that the typical smear positivity rate is 10-20%.† Higher or lower proportions suggest the need for quality assurance procedures to ensure that screening and testing are being carried out appropriately.
2. Bacteriologic Confirmation	70-90%[4,5]	WHO-commissioned systematic reviews cited in the WHO guidelines suggest that microbiologic tests detect just 70-90% of TB cases relative to a reference standard that includes clinically diagnosed cases.
3. Linkage to TB Treatment	100%[3]	WHO guidelines state that all patients who are diagnosed with active TB should be linked to TB treatment.
4. Treatment Success	≥90%[6]	WHO guidelines state that all patients at risk for TB should be screened.
5. TB-free Survival	≥95%[7]	A high-quality systematic review suggests that ≥95% of patients with drug-susceptible TB can be cured with standard 6-month drug regimens.

For peer review only

Appendix S3 continued. Rationale for Zero TB Indicator targets provided in Table 1.

**PREVENT CASCADE**		
TB Screening	100%[8]	WHO guidelines state that all close contacts of TB patients should be screened for active TB.
6. TB Evaluation	≥90%[6]	The END TB Strategy has set a target TB evaluation rate of ≥90% of symptomatic close contacts.
7. (Active) TB Diagnosis	10-20%[2]	Stop TB Partnership sputum microscopy guidelines state that the typical positivity rate is 10-20%.† Higher or lower proportions suggest the need for quality assurance procedures to ensure that screening and testing are being carried out appropriately.
TPT Eligibility	95%[9]	
8. (TPT) Prescription	≥90%[6]	The END TB Strategy has set a target prescription rate for TB preventive therapy of ≥90% of eligible individuals.
9. (TPT) Uptake	100%[6]	The END TB Strategy has set a target uptake rate for TB preventive therapy of ≥90% of eligible individuals
10. (TPT) Completion	≥80%[9]	WHO guidelines state that TPT regimen is considered complete when ≥80% doses have been completed.
11. TB-free survival	100%[9]	Systematic reviews cited within WHO guidelines had a very low TB incidence among those completing TB preventive therapy.

Abbreviations: TB, tuberculosis.
Legend: *Indicator numbers as proposed by the Zero TB Indicator Framework[10]. †The target proportion might be adjusted if a different diagnostic testing modality is in use, or based on robust empirical data.

For peer review only

SUPPLEMENTAL REFERENCES

1. World Health Organization. WHO operational handbook on tuberculosis. Module 2: Screening. Systematic screening for tuberculosis disease. Geneva: World Health Organization; 2021.
2. Stop TB Partnership. Laboratory diagnosis of tuberculosis by sputum microscopy. In: Global Laboratory Initiative, editor. Adelaide, Australia: SA Pathology; 2013.
3. World Health Organization. WHO consolidated guidelines on tuberculosis: module 4: treatment: drug-resistant tuberculosis treatment: World Health Organization; 2020.
4. World Health Organization. Policy statement: The use of liquid medium for culture and DST in low- and middle-income country settings. Geneva: World Health Organization; 2007.
5. World Health Organization. WHO Meeting Report of a Technical Expert Consultation: Non-inferiority analysis of Xpert MTB/RIF Ultra compared to Xpert MTB/RIF Geneva: World Health Organization; 2017.
6. World Health Organization. The End TB Strategy. Geneva: World Health Organization; 2015.
7. Imperial MZ, Nahid P, Phillips PPJ, et al. A patient-level pooled analysis of treatment-shortening regimens for drug-susceptible pulmonary tuberculosis. *Nat Med* 2018; **24**(11): 1708-15.
8. World Health Organization. Recommendations for investigating contacts of persons with infectious tuberculosis in low- and middle-income countries. Geneva: World Health Organization; 2012.
9. World Health Organization. Latent tuberculosis infection: Updated and consolidated guidelines for programmatic management. Geneva: World Health Organization; 2018.
10. Yuen C, Becerra M, Codlin A, et al. A best-practice framework of program indicators for monitoring a comprehensive approach to the tuberculosis epidemic: Zero TB Initiative, 2017.

For peer review only

Appendix S1 – Reflexivity Statement

1. **How does this study address local research and policy priorities?**

   This research question was developed to advance the priorities of the Mongolia Zero Tuberculosis Initiative and was conceived by the co-senior authors to provide a tool for assessment of the quality of TB care in Mongolia.

2. **How were local researchers involved in study design?**

   The study design was developed through a series of conversations involving all of the authors, many of whom are local researchers and/or current or former local public health officials.

3. **How has funding been used to support the local research team?**

   All available funding was allocated to a subcontract to support local researchers.

4. **How are research staff who conducted data collection acknowledged?**

   They are co-authors.

5. **Do all members of the research partnership have access to study data?**

   Yes, upon request of the local NGO that collected and cleaned the research data.

6. **How was data used to develop analytical skills within the partnership?**

   The first-author is a young investigator who worked closely with local and international colleagues on the analysis, including adapting the Zero TB Framework to fit locally collected indicators, and preparing the initial draft of the Methods section and helping design some of the tables.

7. **How have research partners collaborated in interpreting study data?**

   After the study team completed the initial analysis, presentations were given to local public health stakeholders to invite their feedback on the analysis and interpretation of the data.

8. **How were research partners supported to develop writing skills?**

   The first author drafted the initial version of the Methods and Discussion, and key sections of the Results.

9. **How will research products be shared to address local needs?**

   The results have been shared with a new set of Mongolian public health authorities appointed following a recent election. We are discussing a new research collaboration to improve TB preventative therapy based on opportunities identified through this analysis.

10. **How is the leadership, contribution and ownership of this work by LMIC researchers recognised within the authorship?**

    Key positions within the manuscript have been shared among researchers based in HIC and those based in LMIC, and critical work in study design, data collection, and interpretation recognized by assigning first authorship.

11. **How have early career researchers across the partnership been included within the authorship team?**

    The first- and second-authors are early career researchers.

For peer review only

12. How has gender balance been addressed within the authorship?

The manuscript includes a healthy mixture of women and men.

13. How has the project contributed to training of LMIC researchers?

Several LMIC researchers, including the first author were closely mentored by the senior authors in drafting a study protocol, IRB proposal, design of case record forms, and problem solving in the field through regular calls and meetings.

14. How has the project contributed to improvements in local infrastructure?

This specific project has not directly contributed to improvements in local infrastructure.

15. What safeguarding procedures were used to protect local study participants and researchers?

We did not identify issues, outside the usual considerations of research ethics, during our study that would put patients or researchers at risk. When potential sensitive topics arose, such as whether data could be collected on potentially sensitive data about personal habits and behaviors, we asked about community and research norms before proposing collection of such data.

For peer review only

BMJ Open

Assessing the Quality of Tuberculosis Care Using Routine Surveillance Data: A process evaluation employing the Zero TB Indicator Framework in Mongolia

Journal:	*BMJ Open*
Manuscript ID	bmjopen-2022-061229.R1
Article Type:	Original research
Date Submitted by the Author:	05-Jun-2022
Complete List of Authors:	Saranjav, Ariunzaya; Mongolian Health Initiative, Zero TB Mongolia<br>Parisi, Christina; Yale University School of Public Health<br>Zhou, Xin; Yale University School of Public Health<br>Dorjnamjil, Khulan; Mongolian Health Initiative<br>Samdan, Tumurkhuyag; Mongolian Health Initiative<br>Erdenebaatar, Sumiya; Mongolian Health Initiative<br>Chuluun, Altantogoskhon; Ulaanbaatar City Health Department, Governor’s Office of Capital City Ulaanbaatar<br>Dalkh, Tserendagva; Mongolian National University of Medical Sciences<br>Ganbaatar, Gantungalag ; National Center for Communicable Diseases, Mongolia<br>Brooks, Meredith; Harvard Medical School, Department of Global Health and Social Medicine<br>Spiegelman, Donna; Yale University School of Public Health, Center for Methods in Implementation and Preventive Science and Department of Biostatistics<br>Ganmaa, Davaasambuu ; Harvard University T H Chan School of Public Health<br>Davis, J. Lucian; Yale University School of Public Health, Epidemiology of Microbial Diseases; Yale School of Medicine,
**Primary Subject Heading**:	Public health
Secondary Subject Heading:	Infectious diseases
Keywords:	Public health < INFECTIOUS DISEASES, Respiratory infections < THORACIC MEDICINE, Tuberculosis < INFECTIOUS DISEASES, Quality in health care < HEALTH SERVICES ADMINISTRATION & MANAGEMENT

SCHOLARONE™ Manuscripts

I, the Submitting Author has the right to grant and does grant on behalf of all authors of the Work (as defined in the below author licence), an exclusive licence and/or a non-exclusive licence for contributions from authors who are: i) UK Crown employees; ii) where BMJ has agreed a CC-BY licence shall apply, and/or iii) in accordance with the terms applicable for US Federal Government officers or employees acting as part of their official duties; on a worldwide, perpetual, irrevocable, royalty-free basis to BMJ Publishing Group Ltd ("BMJ") its licensees and where the relevant Journal is co-owned by BMJ to the co-owners of the Journal, to publish the Work in this journal and any other BMJ products and to exploit all rights, as set out in our licence.

The Submitting Author accepts and understands that any supply made under these terms is made by BMJ to the Submitting Author unless you are acting as an employee on behalf of your employer or a postgraduate student of an affiliated institution which is paying any applicable article publishing charge ("APC") for Open Access articles. Where the Submitting Author wishes to make the Work available on an Open Access basis (and intends to pay the relevant APC), the terms of reuse of such Open Access shall be governed by a Creative Commons licence – details of these licences and which Creative Commons licence will apply to this Work are set out in our licence referred to above.

Other than as permitted in any relevant BMJ Author's Self Archiving Policies, I confirm this Work has not been accepted for publication elsewhere, is not being considered for publication elsewhere and does not duplicate material already published. I confirm all authors consent to publication of this Work and authorise the granting of this licence.

For peer review only

Assessing the Quality of Tuberculosis Care Using Routine Surveillance Data: A process evaluation employing the Zero TB Indicator Framework in Mongolia

Saranjav Ariunzaya, MD[*1], Christina Parisi, MPH[*5,9], Xin Zhou, PhD[6,7], Dorjnamjil Khulan, MD[1], Samdan Tumurkhuyag, BSc[1], Erdenebaatar Sumiya, BSc[1], Chuluun Altantogoskhon, MD[2], Dalkh Tserendagva, PhD[3], Ganbaatar Gantungalag, MPH[4], Meredith B. Brooks, PhD[10], Donna Spiegelman, ScD[6,7], Davaasambuu Ganmaa[†], MD[1, 11], J. Lucian Davis[†], MD[5,7,8]

*Authors contributed equally; †Authors contributed equally

Corresponding Author:

Dr. Saranjav Ariunzaya, Mongolian Health Initiative, Zero TB Mongolia, Ulaanbaatar, Mongolia

Affiliations:

[1]Mongolian Health Initiative, [2]Ulaanbaatar City Health Department, Governor's Office of Capital City Ulaanbaatar, [3]International School of Mongolian Medicine, Mongolian National University of Medical Sciences, and [4]National Center for Communicable Disease, Ulaanbaatar, Mongolia;

[5]Departments of Epidemiology of Microbial Diseases and [6]Biostatistics, and the [7]Center for Methods in Implementation and Prevention Science, Yale School of Public Health, and [8]Pulmonary, Critical Care, and Sleep Medicine Section, Yale School of Medicine, New Haven, Connecticut, USA

[9]Department of Epidemiology, University of Florida, Gainesville, Florida, USA

[10]Department of Medicine, Brigham and Women's Hospital, Harvard Medical School, and [11]Department of Nutrition, Harvard School of Public Health, Boston, Massachusetts, USA

For peer review only

For peer review only - http://bmjopen.bmj.com/site/about/guidelines.xhtml

Funding: This work was sponsored by a grant from the National Institute of Environmental Health Sciences (DP1 ES025459 to DS) and an anonymous foundation based in the United Kingdom. CP is funded by NIAAA T32AA025877.

Author Email addresses:

1. Saranjav Ariunzaya, ariunzaya.sar@gmail.com
2. Christina Parisi, christina.parisi@ufl.edu
3. Xin Zhou, xin.zhou@yale.edu
4. Dorjnamjil Khulan, khulan2111@gmail.com
5. Samdan Tumurkhuyag, tumurkhuyagsamdan@gmail.com
6. Erdenebaatar Sumiya, sumiya.mhisumiya@gmail.com
7. Chuluun Altantogoskhon, achuluun95@gmail.com
8. Dalkh Tserendagva, tserendagva_d@yahoo.com
9. Gantungalag Ganbaatar, gantungalag22@gmail.com
10. Meredith Brooks, Meredith_Brooks@hms.harvard.edu
11. Donna Spiegelman, donna.spiegelman@yale.edu
12. Davaasambuu Ganmaa, gdavaasa@hsph.harvard.edu
13. J. Lucian Davis, lucian.davis@yale.edu

Word Count: 4123

Keywords: tuberculosis; implementation science; cascade of care; continuum of care; quality of care; process evaluation

For peer review only

ABSTRACT

Objectives: To evaluate the feasibility of the Zero TB Indicator Framework as a tool for assessing the quality of tuberculosis (TB) case-finding, treatment, and prevention services in Mongolia.

Setting: Primary health centers, tuberculosis dispensaries, and surrounding communities in four districts of Mongolia.

Design: Three retrospective cross-sectional cohort studies, and two longitudinal studies each individually nested in one of the cohort studies.

Participants: 15,947 community members from high tuberculosis-risk populations; 8,518 patients screened for tuberculosis in primary health centers and referred to dispensaries; 857 index tuberculosis patients and 2,352 household contacts.

Primary and Secondary Outcome Measures: 14 indicators of the quality of tuberculosis care defined by the Zero TB Indicator Framework and organized into three care cascades, evaluating community-based active case-finding, passive case finding in health facilities, and TB screening and prevention among close contacts; individual and health-system predictors of these indicators.

Results: The cumulative proportions of participants receiving guideline-adherent care varied widely, from 96% for community-based active case-finding, to 79% for TB preventive therapy among household contacts, to only 57% for passive case-finding in primary health centers and TB dispensaries (range 29% to 80% across districts). The odds of patients completing active TB treatment decreased substantially with increasing age (aOR 0.76 per decade, 95%CI 0.71-0.83, $p<0.001$) and among men (aOR 0.56, 95%CI

For peer review only

0.36-0.88, p=0.013). Contacts of older index patients also had lower odds of initiating and completing of TB preventive therapy (aOR 0.60 per decade, 95%CI 0.38-0.93, p=0.022).

Conclusions: The Zero TB Framework provided a feasible and adaptable approach for using routine surveillance data to evaluate the quality of TB care and identify associated individual and health system factors. Future research should evaluate strategies for collecting process indicators more efficiently; gather qualitative data on explanations for low-quality care; and deploy quality improvement interventions.

Abstract Word Count (Limit 300 words): 293

Strengths and limitations of this study:

- This study provides a comprehensive evaluation of the quality of TB diagnostic, treatment, and prevention services in Mongolia using routine surveillance data.
- This study provides a method for identifying individual and health-system factors associated with lower quality care, which may facilitate targeting of quality improvement interventions.
- Routine tuberculosis (TB) surveillance data is susceptible to outcome misclassification in the absence of unique identifiers.
- Results may not be generalizable to all TB units in Mongolia given the purposive sampling strategy.

For peer review only

INTRODUCTION

Tuberculosis (TB) incidence and mortality rates have been declining worldwide for two decades, but with recent global disruptions to TB services and surging poverty rates related to the COVID-19 pandemic, the absolute number of annual TB cases (10 million) and deaths (1.4 million) is likely to increase significantly in coming years (1). As the global TB community prepares for surging caseloads and declining resources, the crisis in the quality of TB care has never been more salient. Before the pandemic, one-third of TB patients did not access care or were not reported to public health authorities, and even among those who sought care for TB, there was wide geographic variability in treatment coverage and success rates (2). Other evidence-based strategies for reducing TB incidence, including active case finding (3, 4) and TB preventive therapy (5) remain unavailable in most low and middle-income countries.(6) The Lancet Commission on High Quality Health Systems has estimated that almost 50% of annual TB deaths may be attributable to low-quality care (7).

Although improving access to high-quality services is the ultimate goal, there is first a need for a revolution in measuring and reporting on quality. The general metrics of high-quality care—safety, timeliness, effectiveness, efficiency, equity, patient-centeredness, and integration of services—are widely agreed upon (8, 9). However, these metrics have not been widely adapted or adopted for reporting data from TB programs in real time. For example, national TB programs have traditionally focused public health reporting on treatment coverage and treatment success rates, two narrow measures of effectiveness, and only recently have added process measures such as the proportion of eligible patients initiating TB preventive therapy (10-12). If improving quality is to be “hard-wired” into national TB programs, as a recent Lancet Commission on Tuberculosis recommended (13), there is an urgent need for research on expanding measures of quality for use as benchmarks, as called for by the

For peer review only - http://bmjopen.bmj.com/site/about/guidelines.xhtml

WHO TB Program (14) and others (15), starting with measures that make better use of routine TB surveillance data (16).

Structured evaluations of care processes through cascade analyses have increasingly been proposed (15, 17-22) and implemented to describe gaps in quality of TB care in low- and middle-income countries, including for clinic-based TB diagnosis and treatment in adults (23-32) and children (33, 34), active case-finding (29, 35, 36), and prevention (29, 37, 38) (Appendix S1). The Zero TB Initiative (39) has introduced an expansive quality indicator framework that integrates all three of these cascade types (19); similar approaches have been proposed for persons living with HIV (18). Here, we evaluate the feasibility of the Zero TB Indicator Framework as a tool for assessing the quality of TB services, using data from Mongolia, a Zero TB Initiative country.

METHODS

Setting

Mongolia, a lower middle-income country of 3 million people, has the fourth-highest TB incidence in the WHO Western Pacific Region, at 428 cases per 100,000, and among the world's lowest TB treatment coverage rates, at 31% (1). Mongolia's high TB prevalence rate (757 per 100,000) exceeds its annual TB incidence rate, indicating a prolonged average duration of illness and the presence of diagnostic delays before treatment (12, 40). More favorably, Mongolia's TB treatment success rate of 91% surpasses the global average, and the country funds 77% of its TB program from domestic sources (6).

For peer review only - http://bmjopen.bmj.com/site/about/guidelines.xhtml

The Mongolian Government provides free TB services in centralized TB dispensaries located in every urban district and rural province. National TB Program (NTP) guidelines recommend TB symptom screening of all individuals attending primary health centers and referral of those screening positive to centralized dispensaries for diagnostic evaluation with sputum smear microscopy and chest radiography. Molecular testing is available only in select dispensaries and the National Center for Communicable Diseases (NCCD), where mycobacterial culture is also available. NTP guidelines recommend active TB case-finding among high-risk groups (people living with HIV; miners; those unhoused, incarcerated, or living in poverty; people living in remote areas; healthcare workers; school staff; orphans) using community-based symptom screening and referral to dispensaries for chest radiography. NTP guidelines also recommend referral of all household contacts of sputum smear-positive TB patients to dispensaries for TB symptom screening and chest radiography, plus tuberculin skin testing (TST); TST-positive children without evidence of active TB are eligible for TB preventive therapy with six months of daily isoniazid.

The NTP oversees management of the paper registries used for TB reporting, as well as the entry of data from these source documents into the national electronic TB information database. NTP compiles surveillance data quarterly and provides technical oversight and assistance to TB providers in Mongolia’s 21 provinces and 9 capital city districts. Since 2017, the NTP has implemented enhanced human resource management practices for health workers in TB dispensaries, including on-the-job training in TB care, performance incentives, and retirement benefits.

Study Design, Participants, and Sites

For peer review only - http://bmjopen.bmj.com/site/about/guidelines.xhtml

Between January 1 and December 31, 2017, we retrospectively performed three retrospective cross-sectional cohort studies, and two longitudinal studies each nested in one of the cohort studies, to construct the three Zero TB Indicator Framework cascades. For the Search, Treat, and Prevent cascades, we conducted retrospective cross-sectional cohort studies. For the Treat and Prevent cascades, we had access to individual data, so we were able to conduct nested longitudinal cohort studies to examine long-term outcomes of treatment in addition to the baseline cross-sectional cohort studies. The Search cascade examined community-based, active TB case-finding among selected high-risk populations (people living in poverty; school staff and students; household contacts of TB index patients) screened by NTP at NCCD and its partner non-governmental organizations ((NGOs), *e.g.*, Korean National Tuberculosis Association; Mongolian Health Initiative). High-risk populations were identified for screening using data from the Mongolian National Statistics Office on census tracts having lower socioeconomic status, recommendations from TB unit staff of a school with a recent TB outbreak, and records from TB index cases, but only in the capital city districts. The Treat cascade examined clinic-based TB diagnosis and treatment among possible TB patients identified in primary health centers and referred for evaluation at TB dispensaries, including first episodes of care only. The indications for referral followed NTP guidelines and included reporting one or more TB symptoms, defined as a cough and/or fever lasting ≥2 weeks, weight loss, or blood-tinged sputum; or being found to have abnormalities on chest radiography. Finally, the Prevent cascade examined diagnosis of latent TB infection (LTBI) and delivery of TB preventive therapy (TPT) among household contacts of index TB patients.

Bayanzurkh (population 280,000), Khan-Uul (population 170,000), and Chingeltei (population 150,000) are three densely populated districts among nine total districts in the Mongolian capital, Ulaanbaatar, all of which have both urban and rural areas. Mandal is the highest-population sub-province in rural

For peer review only - http://bmjopen.bmj.com/site/about/guidelines.xhtml

Selenge province and has established active surveillance and screening programs at high schools; and Chingeltei because it was recognized as a model district at the 2018 National TB Forum. Mandal has two prisons and three large mines, both of which are high-risk settings for TB. Bayanzurkh has 15 TB staff (7 TB specialist doctors, 6 TB nurses, 2 laboratory technicians); Khan-Uul, 7 TB staff (4 TB specialist doctors, 2 TB nurses, 1 laboratory technicians). Chingeltei, 5 TB staff (2 specialist TB doctors, 2 nurses, 1 laboratory technician); and Mandal 7 TB staff (1 TB doctor, 5 TB nurses, 1 laboratory technician). Each dispensary also has 2-4 TB volunteers.

Measurements

Mongolian Health Initiative staff (S.A., D. K., S.T., E.S.) digitally photographed official NTP laboratory, treatment, and contact registers during site visits. They double-entered demographic and clinical data on possible TB patients, confirmed TB patients, and household TB contacts from these photos into electronic data collection forms (REDCap, Nashville, TN, USA). They also extracted aggregated data on active case-finding from NGO reports to the NTP, and on TB symptom screening in primary health centers from records of the Mongolian Association of Family Medicine Specialists.

We successfully defined and adapted 14 of 16 Zero TB Indicators to fit data routinely collected by the Mongolia NTP and partners (Table 1) (19), and proposed evidence-based performance targets derived from published guidelines or systematic reviews (Appendix S2). We could not obtain data on which individuals were TB-free one year after completing active TB treatment or TPT because post-treatment follow-up is not routinely performed in Mongolia. Patients with TB treatment failure are managed according to national guidelines, which in 2017 recommended repeat sputum examination for

For peer review only - http://bmjopen.bmj.com/site/about/guidelines.xhtml

mycobacterial culture and drug-susceptibility testing, and empiric initiation of a WHO Category II re-treatment regimen.

For peer review only

For peer review only - http://bmjopen.bmj.com/site/about/guidelines.xhtml

Table 1 – Process indicators of performance, adapted for Mongolia from the Zero TB Indicator Framework.

Indicator (Proportion)	Numerator (Count)	Denominator (Count)	Interpretation	Target/Source
**SEARCH CASCADE[a]**				
1. (Screening) Coverage	Screened for active TB disease[b]	Estimated individuals in target population$_2$	Effectiveness of outreach to target population	100% (41)
2. Positive TB symptom screen	Positive TB symptoms or CXR screening	Screened for active TB disease	Accuracy & efficiency of screening implementation	25-50% (41)
3. Diagnostic evaluation	Completed TB evaluation[c]	Positive TB symptom or CXR screening	Effectiveness of linkage to testing	100% (41)
4. (Active) TB Diagnosis	Diagnosed with active TB disease[d]	Completed TB evaluation	Yield of diagnostic evaluation	10-20% (42)
5. Linkage to TB Treatment	Prescribed active TB treatment[e]	Diagnosed with active TB disease	Effectiveness of linkage to treatment	100% (43)
**TREAT CASCADE**				
1. (Active) TB Diagnosis	Diagnosed with active TB disease[d]	Evaluated for active TB disease	Yield of diagnostic evaluation	10-20% (42)
2. Bacteriologic Confirmation	MTB-positive bacteriologic test[f]	Diagnosed with active TB disease	Accuracy & efficiency of screening implementation	70-90% (44, 45)
3. Linkage to TB Treatment	Prescribed active TB treatment[e]	Diagnosed with active TB disease	Effectiveness of linkage to treatment	100% (43)
4. Treatment Success	Treatment outcome successful[g]	Started active TB treatment	Effectiveness of treatment	≥90% (11)
5. TB-free Survival	TB-free one year later	Treatment outcome successful	Effectiveness of treatment follow-up	≥95% (46)
**PREVENT CASCADE**				
1. TB Screening	Completed TB screening[h]	Estimated household contacts of smear-positive index TB patients	Effectiveness of outreach to target population	100% (47)
2. TB Evaluation	Completed TB clinical/lab evaluation[i]	Completed TB screening	Effectiveness of linkage to testing	≥90% (11)
3. (Active) TB Diagnosis	Diagnosed with active TB disease[d]	Completed TB clinical/lab evaluation	Yield of diagnostic evaluation	10-20% (42)
4. TPT Eligibility	Confirmed not to have active TB disease & TST-positive[i]	Completed TB clinical/lab evaluation	Accuracy & efficiency of testing implementation	95% (5)

For peer review only - http://bmjopen.bmj.com/site/about/guidelines.xhtml

5. (TPT) Prescription	Prescribed preventive therapy[j]	Eligible for preventive therapy	Adoption of TPT by providers	≥90% (11)
6. (TPT) Uptake	Started preventive therapy	Prescribed preventive therapy	Reach of TPT to patients	
7. (TPT) Completion	Completed preventive therapy	Started preventive therapy	Effectiveness of TPT implementation	≥80% (5)
8. TB-free survival	TB-free one year later	No TB present at initial evaluation	Effectiveness of treatment follow-up	100% (5)

Abbreviations: CXR, chest radiography; NTP, national tuberculosis program; TB, tuberculosis; TPT, TB preventive therapy; TST, tuberculin skin test

Legend: Indicators, cascades, and numbering derived from the Zero TB Indicator Framework (1), with numerators adapted using the Mongolian National TB Guidelines and source data, and targets derived from WHO guidelines and targets. Additional details available in Appendix S2.

[a]The target population included people living in poverty, school staff and students at a school with a recent TB outbreak, and household contacts of TB index patients.

[b]Screening includes questions about a history of TB symptoms (cough or fever lasting ≥2 weeks, weight loss, or bloody sputum) and/or CXR, with any symptom or CXR abnormality (*e.g.*, consolidation, infiltrates, nodules, cavities) defining a positive screen.

[c]Completing TB evaluation is defined as receiving a clinical assessment and all tests required by the TB clinician at the TB dispensary. According to NTP guidelines, these may include direct (sputum smear microscopy, mycobacterial culture, or Xpert MTB/RIF assay) or indirect (biopsy, adenosine deaminase test (ADA), TST) tests.

[d]Diagnosed with active TB disease is defined as being recorded in the National TB Program TB lab register and/or the treatment register as a new TB patient, confirmed as of one of the following categories: bacteriologically confirmed drug-susceptible TB, bacteriologically confirmed drug-resistant TB, bacteriologically confirmed multidrug-resistant TB, bacteriologically confirmed extensively drug-resistant TB, or unconfirmed TB based on clinician judgment.

[e]Defined as being recorded in the National TB Program treatment register as a new TB patient prescribed TB treatment.

[f]Bacteriologic confirmation may occur by sputum smear microscopy, sputum mycobacterial culture, and/or Xpert MTB/RIF.

[g]Treatment outcome successful was defined to include all patients who completed the recommended duration of treatment, whether documented to have negative smear microscopy or a negative mycobacterial culture result within the final month of treatment ("TB cured", per NTP guidelines) or not ("Completed TB treatment", per NTP guidelines). Patients lost to follow-up (*i.e.*, those who did not start treatment or had treatment interrupted for ≥2 consecutive months); those not evaluated; and those who transferred to another treatment unit in a different area, were defined as treatment outcome not successful.

[h]Those eligible for screening for possible preventive TB treatment include all household and other close contacts of a TB index patient. Screening should be performed within 14 days following the diagnosis of an TB index patient.

[i]According to NTP guidelines, contacts 0-15 years of age should be evaluated with a TST (defined as positive for TB infection among close contacts when ≥5 mm skin induration is documented within 2-3 days of placement); TST may be repeated 12 weeks after exposure. For contacts ≥15 years, a chest X-ray (CXR) is required. If TST is positive or CXR abnormalities are identified, bacteriological testing with sputum Xpert MTB/RIF or mycobacterial culture is recommended. If TB is bacteriologically confirmed, active TB treatment is recommended. For children under 5 years of age who are TST positive and in whom active TB has been excluded, TB preventive therapy is recommended, and may be prescribed in other situations (*e.g.*, concerns about false negative results) at the clinician's discretion.

[j]Preventive therapy for children is 10 mg/kg of isoniazid for 6 months. The daily maximum dose of isoniazid is 300 mg.

For peer review only - http://bmjopen.bmj.com/site/about/guidelines.xhtml

Analysis

We presented baseline demographic and clinical characteristics of those completing referral for TB evaluation for the Treat and Prevent studies only; demographic and clinical characteristics were not available for the Search study. We calculated proportions for dichotomous variables and medians with upper and lower quartiles for continuous variables. For t-tests and chi-square tests for significance, a p-value <0.05 was considered significant.

For the process evaluation, we determined indicators for the Search, Treat, and Prevent cascades differently. The Search and Treat cascade indicators were calculated as simple proportions, and the yield as counts diagnosed and initiating treatment divided by total counts screened, with the number-needed-to-screen reported as the inverse of this ratio. Within the Prevent Cascade, we assessed for correlation of process outcomes among contacts within households by calculating Intraclass Correlation Coefficients (ICC). For outcomes with ICC ≥0.10, we estimated the probability of completion by fitting a multivariate, logistic regression model using generalized estimating equations (GEE) (48) with an exchangeable working correlation structure to account for households. For each cascade, we presented indicators within flow diagrams (Figure 1) and calculated stepwise and cumulative probabilities of receiving guideline-adherent care. For the Treat Cascade, we also calculated site-level cumulative probabilities of symptomatic individuals at primary health centers being referred to a dispensary, evaluated for active TB, and starting and completing treatment if diagnosed. We derived a formula to estimate 95% confidence intervals for cumulative probabilities (Appendix S3). We assessed between-site differences in performance using the chi-squared test of independence. Finally, for process outcomes with the greatest losses (*i.e.*, the largest percentage drops in retention between steps), we constructed multivariate, logistic regression models using GEE to identify individual demographic (age, gender)

For peer review only - http://bmjopen.bmj.com/site/about/guidelines.xhtml

predictors of dropout, with 95% CI and p-values provided by Wald tests. We did this for the "starting active TB treatment" and "completing active TB treatment" steps in the Treat cascade (see Supplement Tables S1 and S2) and the "completing TB evaluation" and "being prescribed and initiating TPT" steps in the Prevent cascade (see Supplement Tables S3 and S4). We did not do this for the other steps because completion rates were high and the numbers who were lost were not sufficient to power a predictive model. We based sample size on available participants. We performed all analyses in SAS (Version 9.4, The SAS Institute, Cary, NC, USA). The study protocol was approved by the Yale University institutional review board (IRB), the Harvard School of Public Health IRB, and the Mongolian National University of Medical Sciences IRB as minimal risk research, all of which waived the requirement for informed consent.

Patient and Public Involvement in Research

Patients who concurrently or previously had TB were not involved in defining the research design or implementation, but members of the research team (S.A., D.K.) met intermittently with staff at the primary health centers and the dispensaries during data collection to refine the study measures and to disseminate the research findings.

RESULTS

Search Cascade

For peer review only - http://bmjopen.bmj.com/site/about/guidelines.xhtml
For peer review only

There were 15,947 people in high-risk populations screened for active TB within the four study communities, including 6,670 (42%) in Chingeltei district, 4,632 (29%) in Bayanzurkh district, 3,865 (24%) in Khan-Uul district, and 780 (5%) in Mandal sub-province. This included 12,082 (76%) vulnerable individuals of low socioeconomic status in Chingeltei and Bayanzurkh districts and in Mandal sub-province; and 3,865 (24%) students, teachers, and administrative officials in Khan-Uul district. Of the original 15,947, 15,294 (96%) underwent symptom screening and chest radiography for possible active TB disease (Figure 2). Screening was positive for 1,592 (10%). Of these, 1,571 (99%) completed TB evaluation, with 97 (6%) diagnosed with active TB disease and 89 (92%) starting treatment.

Of all 15,947 community members screened for active TB in the Search Cascade, 15,261 (95.7%) completed each of the required steps of the cascade, an indication of high-quality care (Figure 3a). The cumulative yield of active TB treatments among these communities was 558 per 100,000 screened, providing a number-needed-to-screen of 179.

Treat Cascade

There were 8,518 people who screened positive for TB symptoms at 62 primary health care centers spread across the four communities (Figure 4), including 3245 (38%) from Bayanzurkh, 2620 (31%) from Khan-Uul, 2160 (25%) from Chingeltei, and 493 (6%) from Mandal. Of the 8,518 individuals screened, 1,486 (17%) were assigned non-TB diagnoses. The remaining 7,032 (83%) were referred to dispensaries for TB evaluation, but only 4,416 (63%) arrived and underwent and completed TB testing. Those tested included 2,348 males (53%) and four people living with HIV (0.1%). Median age was 40 years (lower quartile [Q1] 26 to upper quartile [Q3] 55). Of those visiting the dispensaries and tested, 1,162 (26%) were diagnosed with active TB, 824 (71%) of whom were bacteriologically confirmed. Of these, 192 (23%) had drug-resistant TB. Among the 1,124 (97%) who began treatment, 868 (77%) were successfully

For peer review only - http://bmjopen.bmj.com/site/about/guidelines.xhtml
For peer review only

treated, including 81% of drug-susceptible and 55% of drug-resistant cases. Among those who did not complete treatment, 94 (8%) were lost to follow up, 52 (5%) failed treatment, 31 (3%) died, and 79 (7%) had unknown treatment outcomes.

Of all 8,518 individuals entering the Treat Cascade by screening positive for TB symptoms at primary health centers, 5,726 (67.2%) received all services defined as indicative of high-quality care in terms of timeliness and efficiency (8, 9) (Figure 3b).

Treat cascade indicators showed significant variation across sites (Table S0). Among those referred from primary health centers, evaluation rates ranged widely, from nearly 100% in Mandal *soum* to just 47% in Bayanzurkh (Chi-squared test for independence, p<0.001). The proportions starting treatment, in contrast, were consistently high, from 93% to 98% across sites (p=0.090). Finally, treatment success varied markedly, with a high of 87% in Mandal *soum* to a low of 72% in Bayanzurkh (p<0.001). The cumulative probabilities of being properly diagnosed and treated for TB given a positive TB diagnosis also varied substantially(p<0.001) and were highest at Mandal *soum* (80%, 95% CI 68%-92%) and lowest at Bayanzurkh (29%, 95% CI 27%-31%), with similarly low probabilities at Khan-Uul (42%, 95% CI 40%-45%) and Chingeltei (44%, 95% CI 41%-47%).

The odds of starting treatment among active TB patients were lower among older individuals (adjusted Odds Ratio [aOR] 0.72 per decade, 95%CI 0.66-0.79, p<0.001) and males (aOR 0.73, 95%CI 0.62-0.86, p<0.001) (Table S1). The odds of completing TB treatment were also lower in older individuals (aOR 0.76 per decade; 95% CI 0.71-0.83, p<0.001), and males (aOR 0.56; 95% CI 0.36-0.88, p=0.013) (Table S2).

Prevent Cascade

For peer review only - http://bmjopen.bmj.com/site/about/guidelines.xhtml

Among 857 index patients diagnosed with smear-positive active TB, 75 lived alone or did not report on household contacts, leaving 782 (91%) eligible index patients with 2,352 household contacts (mean 3 household contacts per household) eligible for contact investigation. Among contacts in the same household, there was modest correlation in eligibility for TPT (ICC=0.14), and a moderate-to-high correlation for evaluation completion (ICC=0.30) and prescription and initiation of TPT (ICC=0.41).

Of the 2352 household contacts in the target population, including 270 children under 5 years old, only 1932 (82%, 95%CI 80-84%) visited the dispensary and were evaluated for active TB (Figure 5).

Older patients were less likely to complete TB evaluation (aOR 0.92 per decade, 95%CI 0.86-0.98, p=0.015), after adjusting for the gender of the contact, the contact's relationship to index patient, and the age and gender of the index patient (Table S3).

Of the household contacts who reached the dispensary, 179 (9%) were diagnosed with active TB, yielding 7,600 cases per 100,000, providing a number-needed-to-screen among all household contacts of 13. Concurrently, 1753 (91%) contacts were found not to have active TB, including 254 (15%; 95%CI 13-16%) under 5 years of age who were eligible for screening for latent TB infection (LTBI). Eighty-six (34%; 95%CI 29-41%) child contacts were tuberculin skin test (TST) positive. Among these, 36 (43%; 95%CI 33-55%) were prescribed and initiated IPT and all 36 (100%) completed TPT. Of all 8,518 individuals entering the Prevent Cascade as household contacts being screened for active TB, 1,868 (79.4%) received all services defined as indicative of high-quality care in terms of timeliness and efficiency (8, 9) (Figure 3c).

Older index patient age was associated with substantially lower odds of an eligible child being prescribed preventive therapy (aOR 0.60 per decade, 95%CI 0.38-0.93, p=0.022), after adjusting for age

For peer review only - http://bmjopen.bmj.com/site/about/guidelines.xhtml

and gender of the contact, the contact's relationship to index patient, and gender of the index patient (Table S4).

DISCUSSION

We systematically evaluated the quality of TB care at the sub-national and facility levels by applying the Zero TB Indicator Framework to routinely collected TB surveillance data from four administrative regions of Mongolia. Overall, the quality of care in the Search Cascade was high, with almost everyone receiving all recommended active case-finding services. In contrast, the quality of care in the Prevent Cascade was only moderate-to-high, with only four out of every five receiving all recommended household TB screening and prevention services, and the quality of care in the Treat Cascade was only moderate, with only two out of every three receiving all recommended passive case finding and treatment services. The exercise also enabled us to localize important gaps in the efficiency, individual and public health effectiveness, and equity of TB active and passive case finding and prevention in reference to evidence-based standards. In addition, we identified individual and facility-level factors associated with poor outcomes, providing a starting point for quality improvement interventions.

This work is an important contribution in several ways. First, it is among the first efforts to assimilate analyses of quality across the spectrum of TB services, including TB active case-finding, TB diagnosis and treatment, and TB preventive therapy simultaneously. Second, the cascades were constructed directly using routinely collected data, without the requirement for intermediate epidemiological assumptions, an important advance toward the goal of integrating actionable, real-time quality assessments into routine TB surveillance at local level. Third, using the Zero TB Indicator Framework to achieve this goal

For peer review only - http://bmjopen.bmj.com/site/about/guidelines.xhtml

provided the opportunity to evaluate its feasibility as a tool for monitoring the quality of TB care in a resource-constrained, high TB-incidence country, a critical need in global public health.

In addition, our analysis illustrates how the systematic evaluation of care processes using the Zero TB Indicator framework could enable national programs to identify areas for additional evaluation and quality improvement activities. First, in Mongolia, although 96% of community members invited to screen in the “Search” cascade ultimately received all recommended services, the cumulative effectiveness as inferred from the diagnostic and treatment yield was unexpectedly low, barely exceeding the national TB prevalence rate. The low yield is partly attributable to the use of sputum smear microscopy for confirmatory testing, a low sensitivity diagnostic test in the context of active-case finding for early stage disease, rather than mycobacterial culture or molecular methods as recommended in international guidelines (49). These higher quality diagnostics remain prohibitively costly, even in middle-income countries; continued efforts are needed to increase financial commitments to advance TB elimination efforts by implementing internationally recommended diagnostics. Also, the absolute number of participants reached and the additional cases identified by these small, short-term active case-finding initiatives were modest, pointing to the need to better target active case-finding towards TB “hotspots” (50) and to sustain screening efforts over time (4). In contrast, the yield of active TB case finding among household contacts in the Prevent Cascade was an order of magnitude higher and contact investigation also facilitated TB prevention efforts, potentially offering much greater value.

For peer review only - http://bmjopen.bmj.com/site/about/guidelines.xhtml

Second, we identified the largest gaps in quality of care within the Treat cascade, where small numbers of patients dropping out from individual care processes accumulated into large losses across the cascade. In Mongolia's centralized system for TB evaluation, one-third of symptomatic individuals did not reach diagnostic centers, a metric that varied widely by geographic area. Even after patients reached TB dispensaries, diagnostic yield and treatment completion also varied widely. While one low-volume, rural site delivered effective diagnosis and treatment to 80% of those presenting for evaluation, only one-quarter to one-half of patients at the other three sites completed all the Treat Cascade steps that we defined as high quality of care. In addition, treatment completion was below the national average at all four sites, especially among older adults and men, who were also less likely to initiate treatment than younger adults and women (6). These data suggest a need to further investigate patient-level barriers to accessing diagnostics and treatment at Mongolia's centralized TB dispensaries. In a systematic review of nearly 60 studies of TB diagnostic and treatment delay worldwide, initial presentation to low-level government facilities, geographic isolation, and poverty were three of the most important risk factors (51). According to a 2017 Mongolia NTP survey, >50% TB patients are unemployed and 70% have incomes below the poverty line. If our data are representative of the broader symptomatic population identified at primary health centers, as found elsewhere (52, 53), travel and related opportunity costs may be prohibitive to completing referrals and treatment. Decentralizing TB care to primary health centers is one policy intervention to be considered. In a small study from Southern Brazil, a variety of diagnostic and treatment outcomes were better in a municipality with decentralized care than in a comparable municipality with centralized care (54). In central Sulawesi, Indonesia, a TB program that began offering education, evaluation, and treatment in a community-based setting tripled their case-detection rates relative to baseline and also increased treatment completion considerably (55).

For peer review only - http://bmjopen.bmj.com/site/about/guidelines.xhtml

Third, in our evaluation of the Prevent cascade, almost 80% of household contacts completed TB evaluation, approaching the WHO's 90% target (56). This compares favorably to the 20-53% who completed contact investigation in prior studies, and the number needed to screen of 13 was far below the 56 reported in a prior systematic review (35, 36, 57, 58). Nevertheless, over half of child contacts eligible for TPT did not start it, similar to results reported elsewhere (58-60), and the likelihood of initiation decreased substantially with increasing age of index patients. In a prior systematic review (37), a low perceived risk of active TB for one's children was among the most frequent reasons for low uptake, emphasizing the need for personalized education and counseling to address parental concerns. In addition, high rates of TPT completion among a small group children and high rates of referral completion among adults suggest additional capacity to expand TPT in Mongolia to older child and adult contacts, as recommended by the 2018 WHO guidelines (5).

Strengths

Our study had several strengths. Most prior studies have focused on only one specific aspect of TB services such diagnosis and treatment, or prevention. In contrast, we adopted a more comprehensive perspective (20), evaluating multiple evidence-based interventions concurrently using 14 indicators within the Zero TB Indicator Framework. We successfully adapted the indicators to fit local data and guidelines and developed evidence-based targets as comparators. Second, building on previous studies that relied on research data, aggregated national data, or incorporated epidemiologic assumptions about TB prevalence (61), we generated cascades directly from data collected at the basic TB management unit and referring diagnostic centers. We believe this approach could help address the global need to operationalize standardized measures of quality within routine data collection systems,

For peer review only - http://bmjopen.bmj.com/site/about/guidelines.xhtml

so that cascades may be actionable at facility and district level (14, 27). Although this study's manual extraction procedures would not be feasible for routine use, our approach could help justify adapting routine TB registers to include process measures of quality. Third, we identified individual and health-system factors associated with failure to complete key steps of the cascade, providing important information for tailoring future quality improvement interventions.

Limitations

Our study also had some limitations. First, we were unable to evaluate one-year post-completion outcomes of active TB treatment and TB preventive therapy as called for by the Zero TB indicator Framework. Although this follow-up data is important for evaluating effectiveness, few programs in high-incidence, resource-constrained countries collect it, and the feasibility and costs of doing so must be considered given low rates of relapse of active TB (46) or progression of latent TB after treatment completion (5). Second, our study sites may not be representative of all sites in Mongolia, given our purposive selection strategy. However, evaluating a large random sample was beyond the scope of this preliminary evaluation, so we selected communities representing different population densities, housing types, income levels, and economic settings. Additionally, the high quality of Search cascade services may reflect the greater resources available to the implementing NGOs and therefore not be generalizable. Third, some records may have been duplicated because we relied on aggregated data for some analyses and lacked unique identifiers to link different visits to the same individual. Similarly, some outcome misclassification may be related to inaccurate tracking of handwritten records across multiple official primary data sources, and some records were incomplete. These challenges highlight the need to standardize and expand use of Mongolia's nascent electronic TB registration system (*i.e.,* TUBIS.mn), to improve both the quality of reporting and the quality of care. In Zambia, for example, regular review and

For peer review only - http://bmjopen.bmj.com/site/about/guidelines.xhtml
For peer review only

cross-validation of TB registers at diagnostic and treatment units was associated with significant reductions in the proportion of non-assessed cases (62) Finally, we did not evaluate the readiness of the systems and structures in these districts to deliver TB care, which would provide important and complementary information for assessing quality (57).

Conclusion

We found that using standardized treatment indicators and cascades to evaluate core processes of evidence-based TB interventions was feasible and informative for evaluating the quality of TB services. This approach may be generalizable to other high-burden settings that employ WHO's standardized approach to TB monitoring and reporting (63, 64). To complement such analyses, there is a need to incorporate qualitative and mixed-methods studies (32, 65, 66) to contextualize the barriers and facilitators of care processes, as well as structural assessments of quality such as service availability and readiness surveys (67), to help enhance the outcomes of TB care (68). Above all, it is time for researchers and practitioners to strengthen routine TB surveillance data systems to collect rigorous, accessible, and reliable data on the quality of TB services worldwide.

For peer review only

For peer review only - http://bmjopen.bmj.com/site/about/guidelines.xhtml

ACKNOWLEDGMENTS

The authors wish to acknowledge the Mongolian National TB Program; doctors, nurses, and staff at the primary health centers and the TB dispensaries who provided the care under evaluation in this project; the patients and community members whose data contributed to this analysis; Courtney Yuen, Mercedes Becerra, and Michael Wilson for their assistance in establishing the Zero TB Initiative in Mongolia; the Mongolian Association of Family Medicine Specialists, and the staff of the Mongolian Health Initiative.

For peer review only

AUTHOR CONTRIBUTIONS

1. Conceptualization: JLD, DG, DS
2. Data Curation: SA, CP, DK, ST, CA
3. Formal Analysis: SA, CP, XZ, JLD
4. Funding Acquisition: DS
5. Methodology: JLD, XZ, DS
6. Project Administration: SA, DG, JLD, GG, DT, CA, MB
7. Verification of Underlying Data: SA, DK, DG, ES
8. Visualization: CP, JLD
9. Writing – Original Draft: SA, CP, JLD
10. Writing – Review & Editing: SA, CP, XZ, DK, ST, ES, CA, DT, GG, MB, DS, DG, JLD

Legend

Saranjav Ariunzaya (SA)

Christina Parisi (CP)

Xin Zhou (XZ)

Dorjnamjil Khulan (DK)

Samdan Tumurkhuyag (ST)

Erdenebaatar Sumiya (ES)

Chuluun Altantogoskhon (CA)

Dalkh Tserendagva (DT)

Ganbaatar Gantungalag (GG)

Meredith Brooks (MB)

Donna Spiegelman (DS)

Davaasambuu Ganmaa (DG)

J. Lucian Davis (JLD)

For peer review only

Funding Statement

This work was sponsored by a grant from the National Institute of Environmental Health Sciences (DP1 ES025459 to DS) and an anonymous foundation based in the United Kingdom. Christina Parisi is funded by NIAAA T32AA025877. The study funders had no role in design, collection, analysis, or interpretation of the data, in writing this report, or in the decision to submit it for publication.

Conflict of Interest Statement

The authors declare that there is no conflict of interest.

Data Availability Statement

The data that support the findings of this study are available from the corresponding author, Ariunzaya Saranjav, upon reasonable request.

Ethics Statement

Institutional review boards at Yale University (#2000025951), the Harvard School of Public Health (#19-1489), and the Mongolian National University of Medical Sciences (#2019/3-10) approved the study protocol as minimal risk research and waived requirements for informed consent.

For peer review only

REFERENCES

1. World Health Organization. Global tuberculosis control: WHO Report 2020. Geneva: World Health Organization; 2020.

2. Cattamanchi A, Berger CA, Shete PB, Turyahabwe S, Joloba M, Moore DAJ, et al. Implementation science to improve the quality of tuberculosis diagnostic services in Uganda. Journal of Clinical Tuberculosis and Other Mycobacterial Diseases. 2020;18:100136.

3. Corbett EL, Bandason T, Duong T, Dauya E, Makamure B, Churchyard GJ, et al. Comparison of two active case-finding strategies for community-based diagnosis of symptomatic smear-positive tuberculosis and control of infectious tuberculosis in Harare, Zimbabwe (DETECTB): A cluster-randomised trial. Lancet. 2010;376(9748):1244-53.

4. Marks GB, Nguyen NV, Nguyen PTB, Nguyen TA, Nguyen HB, Tran KH, et al. Community-wide Screening for Tuberculosis in a High-Prevalence Setting. N Engl J Med. 2019;381(14):1347-57.

5. World Health Organization. Latent tuberculosis infection: Updated and consolidated guidelines for programmatic management. Geneva: World Health Organization; 2018.

6. World Health Organization. Global tuberculosis control: WHO Report 2020. Geneva: World Health Organization, 2020.

7. Kruk ME, Gage AD, Arsenault C, Jordan K, Leslie HH, Roder-DeWan S, et al. High-quality health systems in the Sustainable Development Goals era: time for a revolution. The Lancet Global Health. 2018;6(11):e1196-e252.

8. Delivering Quality Health Services: A Global Imperative for Universal Health Coverage. Geneva: World Health Organization, Organisation for Economic Co-operation and Development, and The World Bank; 2018.

9. Improving Quality of Care in Low- and Middle-Income Countries: Workshop Summary. Buckley GJ, Pittluck RE, editors. Washington, DC: The National Academies Press; 2015. 126 p.

For peer review only

For peer review only - http://bmjopen.bmj.com/site/about/guidelines.xhtml

10. World Health Organization. Framework for conducting reviews of tuberculosis programmes: World Health Organization; 2014.

11. World Health Organization. The End TB Strategy. Geneva: World Health Organization; 2015.

12. World Health Organization. Global tuberculosis control: WHO Report 2019. Geneva: World Health Organization, 2019.

13. Reid MJA, Goosby E. Improving quality is necessary to building a TB-free world: Lancet Commission on Tuberculosis. Journal of clinical tuberculosis and other mycobacterial diseases. 2020;19:100156-.

14. Floyd K, Glaziou P, Houben RMGJ, Sumner T, White RG, Raviglione M. Global tuberculosis targets and milestones set for 2016-2035: Definition and rationale. International Journal of Tuberculosis and Lung Disease. 2018;22(7):723-30.

15. Subbaraman R, Nathavitharana RR, Mayer KH, Satyanarayana S, Chadha VK, Arinaminpathy N, et al. Constructing care cascades for active tuberculosis: A strategy for program monitoring and identifying gaps in quality of care. PLoS Med. 2019;16(2):e1002754.

16. Theron G, Jenkins HE, Cobelens F, Abubakar I, Khan AJ, Cohen T, et al. Data for action: collection and use of local data to end tuberculosis. The Lancet. 2015;386(10010):2324-33.

17. Daftary A, Jha N, Pai M. Enhancing the role of pharmacists in the cascade of tuberculosis care. Journal of Epidemiology and Global Health. 2016;7(1):1.

18. Hanrahan CF, Van Rie A. A proposed novel framework for monitoring and evaluation of the cascade of HIV-associated TB care at the health facility level. Journal of the International AIDS Society. 2017;20(1):21375.

19. Yuen C, Becerra M, Codlin A, Creswell J, Ditiu L, Keshavjee S, et al. A best-practice framework of program indicators for monitoring a comprehensive approach to the tuberculosis epidemic. Zero TB Initiative, 2017.

For peer review only

For peer review only - http://bmjopen.bmj.com/site/about/guidelines.xhtml

20. Cazabon D, Alsdurf H, Satyanarayana S, Nathavitharana R, Subbaraman R, Daftary A, et al. Quality of tuberculosis care in high burden countries: the urgent need to address gaps in the care cascade. Int J Infect Dis. 2017;56:111-6.

21. Agins BD, Ikeda DJ, Reid MJA, Goosby E, Pai M, Cattamanchi A. Improving the cascade of global tuberculosis care: moving from the “what” to the “how” of quality improvement. The Lancet Infectious Diseases. 2019;19(12):e437-e43.

22. Arsenault C, Roder-DeWan S, Kruk ME. Measuring and improving the quality of tuberculosis care: A framework and implications from the Lancet Global Health Commission. J Clin Tuberc Other Mycobact Dis. 2019;16:100112.

23. Davis JL, Katamba A, Vasquez J, Crawford E, Sserwanga A, Kakeeto S, et al. Evaluating tuberculosis case detection via real-time monitoring of tuberculosis diagnostic services. Am J Respir Crit Care Med. 2011;184(3):362-7.

24. Davis JL, Dowdy DW, den Boon S, Walter ND, Katamba A, Cattamanchi A. Test and treat: a new standard for smear-positive tuberculosis. J Acquir Immune Defic Syndr. 2012;61(1):e6-e8.

25. Sun AY, Denkinger CM, Dowdy DW. The impact of novel tests for tuberculosis depends on the diagnostic cascade. Eur Respir J. 2014;44(5):1366-9.

26. Chaisson LH, Katamba A, Haguma P, Ochom E, Ayakaka I, Mugabe F, et al. Theory-informed interventions to improve the quality of tuberculosis evaluation at Ugandan health centers: a quasi-experimental study. PloS one. 2015;10(7):e0132573.

27. Subbaraman R, Nathavitharana RR, Satyanarayana S, Pai M, Thomas BE, Chadha VK, et al. The Tuberculosis Cascade of Care in India’s Public Sector: A Systematic Review and Meta-analysis. PLOS Medicine. 2016;13(10):e1002149.

For peer review only - http://bmjopen.bmj.com/site/about/guidelines.xhtml

For peer review only

28. Naidoo P, Theron G, Rangaka MX, Chihota VN, Vaughan L, Brey ZO, et al. The South African Tuberculosis Care Cascade: Estimated Losses and Methodological Challenges. J Infect Dis. 2017;216(suppl_7):S702-s13.

29. Shapiro AE, van Heerden A, Schaafsma TT, Hughes JP, Baeten JM, van Rooyen H, et al. Completion of the tuberculosis care cascade in a community-based HIV linkage-to-care study in South Africa and Uganda. J Int AIDS Soc. 2018;21(1).

30. Kim J, Keshavjee S, Atun R. Health systems performance in managing tuberculosis: analysis of tuberculosis care cascades among high-burden and non-high-burden countries. J Glob Health. 2019;9(1):010423.

31. Der JB, Grint D, Narh CT, Bonsu F, Grant AD. Where are patients missed in the tuberculosis diagnostic cascade? A prospective cohort study in Ghana. PloS one. 2020;15(3):e0230604.

32. Yasobant S, Bhavsar P, Kalpana P, Memon F, Trivedi P, Saxena D. Contributing Factors in the Tuberculosis Care Cascade in India: A Systematic Literature Review. Risk Management and Healthcare Policy. 2021;Volume 14:3275-86.

33. Marquez C, Davis JL, Katamba A, Haguma P, Ochom E, Ayakaka I, et al. Assessing the quality of tuberculosis evaluation for children with prolonged cough presenting to routine community health care settings in rural Uganda. PloS one. 2014;9(8):e105935.

34. Mwangwa F, Chamie G, Kwarisiima D, Ayieko J, Owaraganise A, Ruel TD, et al. Gaps in the Child Tuberculosis Care Cascade in 32 Rural Communities in Uganda and Kenya. J Clin Tuberc Other Mycobact Dis. 2017;9:24-9.

35. Armstrong-Hough M, Turimumahoro P, Meyer AJ, Ochom E, Babirye D, Ayakaka I, et al. Drop-out from the tuberculosis contact investigation cascade in a routine public health setting in urban Uganda: A prospective, multi-center study. PloS one. 2017;12(11):e0187145.

For peer review only

For peer review only - http://bmjopen.bmj.com/site/about/guidelines.xhtml

36. Diaz G, Victoria AM, Meyer AJ, Nino Y, Luna L, Ferro BE, et al. Evaluating the Quality of Tuberculosis Contact Investigation in Cali, Colombia: A Retrospective Cohort Study. Am J Trop Med Hyg. 2021.QQ

37. Alsdurf H, Hill PC, Matteelli A, Getahun H, Menzies D. The cascade of care in diagnosis and treatment of latent tuberculosis infection: a systematic review and meta-analysis. Lancet Infect Dis. 2016;16(11):1269-78.

38. van de Water B, Meyer T, Wilson M, Young C, Gaunt B, le Roux K. TB prevention cascade at a district hospital in rural Eastern Cape, South Africa. Public Health Action. 2021;11(2):97-100.

39. Zero TB Initiative. The Zero TB Initiative 2021 [cited 2021 January 10]. Available from: https://www.zerotbinitiative.org/.

40. Mongolia Ministry of Health, National Center for Communicable Diseases. Report of the First National Tuberculosis Prevalence Survey in Mongolia, 2014-2015. Ulaanbaatar, Mongolia2016.

41. World Health Organization. WHO operational handbook on tuberculosis: module 2: screening: systematic screening for tuberculosis disease. 2021.

42. Stop TB Partnership. Laboratory diagnosis of tuberculosis by sputum microscopy. In: Global Laboratory Initiative, editor. Adelaide, Australia: SA Pathology; 2013.

43. World Health Organization. WHO consolidated guidelines on tuberculosis: module 4: treatment: drug-resistant tuberculosis treatment: World Health Organization; 2020.

44. World Health Organization. Policy statement: The use of liquid medium for culture and DST in low- and middle-income country settings. Geneva: World Health Organization; 2007.

45. World Health Organization. WHO Meeting Report of a Technical Expert Consultation: Non-inferiority analysis of Xpert MTB/RIF Ultra compared to Xpert MTB/RIF Geneva: World Health Organization; 2017. 11. p.

For peer review only - http://bmjopen.bmj.com/site/about/guidelines.xhtml

46. Imperial MZ, Nahid P, Phillips PPJ, Davies GR, Fielding K, Hanna D, et al. A patient-level pooled analysis of treatment-shortening regimens for drug-susceptible pulmonary tuberculosis. Nat Med. 2018;24(11):1708-15.

47. World Health Organization. Recommendations for investigating contacts of persons with infectious tuberculosis in low- and middle-income countries. Geneva: World Health Organization; 2012.

48. Diggle P, Diggle PJ, Heagerty P, Liang K-Y, Heagerty PJ, Zeger S. Analysis of longitudinal data: Oxford University Press; 2002.

49. World Health Organization. Tuberculosis Diagnostics. Geneva: World Health Organization, 2016.

50. Shrestha S, Reja M, Gomes I, Baik Y, Pennington J, Islam S, et al. Quantifying geographic heterogeneity in TB incidence and the potential impact of geographically targeted interventions in South and North City Corporations of Dhaka, Bangladesh: a model-based study. Epidemiology and Infection. 2021;149:1-27.

51. Storla DG, Yimer S, Bjune GA. A systematic review of delay in the diagnosis and treatment of tuberculosis. BMC Public Health. 2008;8(1):15.

52. Liu X, Thomson R, Gong Y, Zhao F, Squire SB, Tolhurst R, et al. How affordable are tuberculosis diagnosis and treatment in rural China? An analysis from community and tuberculosis patient perspectives. Tropical Medicine & International Health. 2007;12(12):1464-71.

53. Xu L, Gai R, Wang X, Liu Z, Cheng J, Zhou C, et al. Socio-economic factors affecting the success of tuberculosis treatment in six counties of Shandong Province, China. The international journal of tuberculosis and lung disease : the official journal of the International Union against Tuberculosis and Lung Disease. 2010;14(4):440-6.

54. Scheffer MC, Prim RI, Wildner LM, Medeiros TF, Maurici R, Kupek E, et al. Performance of centralized versus decentralized tuberculosis treatment services in Southern Brazil, 2006–2015. BMC Public Health. 2018;18(1):554.

For peer review only

For peer review only - http://bmjopen.bmj.com/site/about/guidelines.xhtml

55. Becx-Bleumink M, Wibowo H, Apriani W, Vrakking H. High tuberculosis notification and treatment success rates through community participation in central Sulawesi, Republic of Indonesia. International Journal of Tuberculosis and Lung Disease. 2001;5(10):920-5.

56. World Health Organization. Implementing the End TB strategy: The essentials. Geneva: World Health Organization, 2015.

57. Kigozi G, Engelbrecht M, Heunis C, Janse van Rensburg A. Household contact non-attendance of clinical evaluation for tuberculosis: A pilot study in a high burden district in South Africa. BMC Infectious Diseases. 2018;18(1):106.

58. Blok L, Sahu S, Creswell J, Alba S, Stevens R, Bakker MI. Comparative meta-analysis of tuberculosis contact investigation interventions in eleven high burden countries. PloS one. 2015;10(3):e0119822.

59. Singh AR, Kharate A, Bhat P, Kokane AM, Bali S, Sahu S, et al. Isoniazid preventive therapy among children living with tuberculosis patients: Is it working? A mixed-method study from Bhopal, India. Journal of Tropical Pediatrics. 2017;63(4):274-85.

60. Yuen CM, Millones AK, Puma D, Jimenez J, Galea JT, Calderon R, et al. Closing delivery gaps in the treatment of tuberculosis infection: lessons from implementation research in Peru. PloS one. 2021;16(2):e0247411.

61. Subbaraman R, Jhaveri T, Nathavitharana RR. Closing gaps in the tuberculosis care cascade: An action-oriented research agenda. Journal of clinical tuberculosis and other mycobacterial diseases. 2020;19:100144-.

62. Mfungwe V, Ota M, Koyama K, Samungole GKV, Takemura Y, Hirao S, et al. 'Transfer out' tuberculosis patients: Treatment outcomes after cross-checking registers, 2012-2013, Lusaka, Zambia. Public Health Action. 2016;6(2):118-21.

For peer review only

For peer review only - http://bmjopen.bmj.com/site/about/guidelines.xhtml

63. World Health Organization. Standards and benchmarks for tuberculosis surveillance and vital registration systems. Geneva: World Health Organization; 2014.

64. World Health Organization. Toolkit for Analysis and Use of Routine Health Facility Data: Guidance for tuberculosis programme managers. Geneva: World Health Organization; 2020.

65. Armstrong-Hough M, Ggita J, Ayakaka I, Dowdy D, Cattamanchi A, Haberer JE, et al. Brief Report: "Give Me Some Time": Facilitators of and Barriers to Uptake of Home-Based HIV Testing During Household Contact Investigation for Tuberculosis in Kampala, Uganda. J Acquir Immune Defic Syndr. 2018;77(4):400-4.

66. Armstrong-Hough M, Ggita J, Turimumahoro P, Meyer AJ, Ochom E, Dowdy D, et al. 'Something so hard': a mixed-methods study of home sputum collection for tuberculosis contact investigation in Uganda. The international journal of tuberculosis and lung disease : the official journal of the International Union against Tuberculosis and Lung Disease. 2018;22(10):1152-9.

67. World Health Organization. Service Availability and Readiness Assessment (SARA): an annual monitoring system for service delivery. In: Health Statistics and Information Systems, editor. Geneva: World Health Organization; 2015.

68. Donabedian A. The quality of Care: How can it be assessed? JAMA. 1988;260(12).

For peer review only

For peer review only - http://bmjopen.bmj.com/site/about/guidelines.xhtml

FIGURE LEGENDS

Figure 1. Zero TB Indicator Framework Cascades, adapted for Mongolia.

Abbreviations: TB, tuberculosis.

Legend: Flow diagrams showing the movement of individuals through each individual step of the three TB delivery cascades within the Zero TB Indicator Framework. These have been modified to fit data available in Mongolia, and color coded to differentiate among the Search, Treat, and Prevent service cascades. Steps framed within a rectangular box are derived from the Zero TB Indicator Framework, while unframed steps indicate additional important steps within the cascades. Solid arrows show the direction of patient flow through the cascades; a dashed arrow shows where excluded individuals re-enter the cascade. Colored box labels correspond to the 16 Zero TB Framework indicators, with superscripts identifying indicator numbers as defined in Table 1; 5 come from the Search Cascade, 5 from the Treat Cascade, and 6 from the Prevent Cascade. The indicators in **bolded text** describe the quality of care at a given step in the cascade, while the non-bolded indicators describe the diagnostic and/or epidemiological yield of that step in TB care. Finally, brief references to the following supplemental tables are located adjacent to the steps where they will identify barriers to TB care through multivariate models: Table S1, Predictors of Starting Active TB treatment (Treat Cascade); Table S2, Predictors of Completing Active TB Treatment (Treat Cascade); Table S3, Predictors of Completing TB Evaluation (Prevent Cascade); Table S4, Predictors of Being Prescribed and Initiating TPT (Prevent Cascade).

For peer review only - http://bmjopen.bmj.com/site/about/guidelines.xhtml
For peer review only

Figure 2. Flow diagram showing the Search Cascade as adapted from the Zero TB Indicator Framework for Mongolia.

Abbreviations: TB, tuberculosis.

Legend: Flow diagrams showing individuals entering and exiting at each step of the three cascades, either appropriately to the left side after having received guideline-adherent care or inappropriately to the right side after having received guideline-nonadherent care. Zero TB Indicators Treat indicators are shown in orange, with superscripts identifying indicator numbers as defined in Table 1. The indicators in **bolded text** describe the quality of care at a given step in the cascade, while the non-bolded indicators describe the diagnostic and/or epidemiological yield of that step in TB care.

For peer review only

For peer review only - http://bmjopen.bmj.com/site/about/guidelines.xhtml

Figure 3. Cumulative proportions of participants receiving high-quality TB care, by delivery cascade

Panel A. Search Cascade (n=15,947)

Panel B. Treat Cascade (n=8,518)

Panel C. Prevent Cascade (n=2352)

Abbreviations: TB, tuberculosis.

Legend: Bar graphs showing the cumulative probability of completing the key processes with each of the three TB delivery cascades. The probabilities for each step, shown as bars, were calculated as the simple proportion of all individuals receiving guideline-recommended care at the end of that step and all previous steps, divided by the total number entering the cascade. Thin elbow connector lines show the percentage lost with each step, calculated as the simple proportion of individuals not receiving guideline-recommended care at that step divided by the total number entering the cascade. Block arrows with discontinuity lines shows steps where individuals exit the cascade having received all guideline-recommended care. The question mark indicates that follow-up data on outcomes at one year were not available as these are not routinely collected in Mongolia.

For peer review only

Figure 4. Flow diagram showing the Treat Cascade as adapted from the Zero TB Indicator Framework for Mongolia

Abbreviations: TB, tuberculosis.

Legend: Flow diagrams showing individuals entering and exiting at each step of the three cascades, either appropriately to the left side after having received guideline-adherent care or inappropriately to the right side after having received guideline-nonadherent care. Zero TB Treat indicators are shown in purple, with superscripts identifying indicator numbers as defined in Table 1. The indicators in **bolded text** describe the quality of care at a given step in the cascade, while the non-bolded indicators describe the diagnostic and/or epidemiological yield of that step in TB care.

For peer review only

Figure 5. Flow diagram showing the Prevent Cascade, as adapted from the Zero TB Indicator Framework for Mongolia.

Abbreviations: TB, tuberculosis.

Legend: Flow diagrams showing individuals entering and exiting at each step of the three cascades, either appropriately to the left side after having received guideline-adherent care or inappropriately to the right side after having received guideline-nonadherent care. The adapted Zero TB Treat indicators are shown in green and numbered using superscripts as defined in Table 1. The indicators in **bolded text** describe the quality of care at a given step in the cascade, while the non-bolded indicators describe the diagnostic and/or epidemiological yield of that step in TB care. *Percentages derived from multivariate, logistic regression models using generalizing estimations and may differ from crude proportions. ?, unknown.

For peer review only

For peer review only - http://bmjopen.bmj.com/site/about/guidelines.xhtml

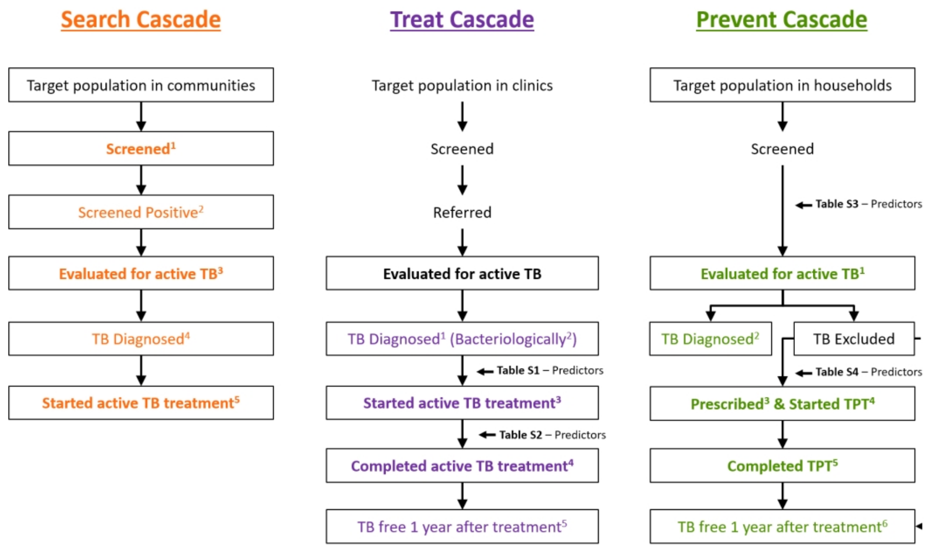

Figure 1. Zero TB Indicator Framework Cascades, adapted for Mongolia.
Abbreviations: TB, tuberculosis.
Legend: Flow diagrams showing the movement of individuals through each individual step of the three TB delivery cascades within the Zero TB Indicator Framework. These have been modified to fit data available in Mongolia, and color coded to differentiate among the Search, Treat, and Prevent service cascades. Steps framed within a rectangular box are derived from the Zero TB Indicator Framework, while unframed steps indicate additional important steps within the cascades. Solid arrows show the direction of patient flow through the cascades; a dashed arrow shows where excluded individuals re-enter the cascade. Colored box labels correspond to the 16 Zero TB Framework indicators, with superscripts identifying indicator numbers as defined in Table 1; 5 come from the Search Cascade, 5 from the Treat Cascade, and 6 from the Prevent Cascade. The indicators in bolded text describe the quality of care at a given step in the cascade, while the non-bolded indicators describe the diagnostic and/or epidemiological yield of that step in TB care. Finally, brief references to the following supplemental tables are located adjacent to the steps where they will identify barriers to TB care through multivariate models: Table S1, Predictors of Starting Active TB treatment (Treat Cascade); Table S2, Predictors of Completing Active TB Treatment (Treat Cascade); Table S3, Predictors of Competing Evaluation (Prevent Cascade); Table S4, Predictors of Being Prescribed and Initiating TPT (Prevent Cascade).

849x481mm (57 x 57 DPI)

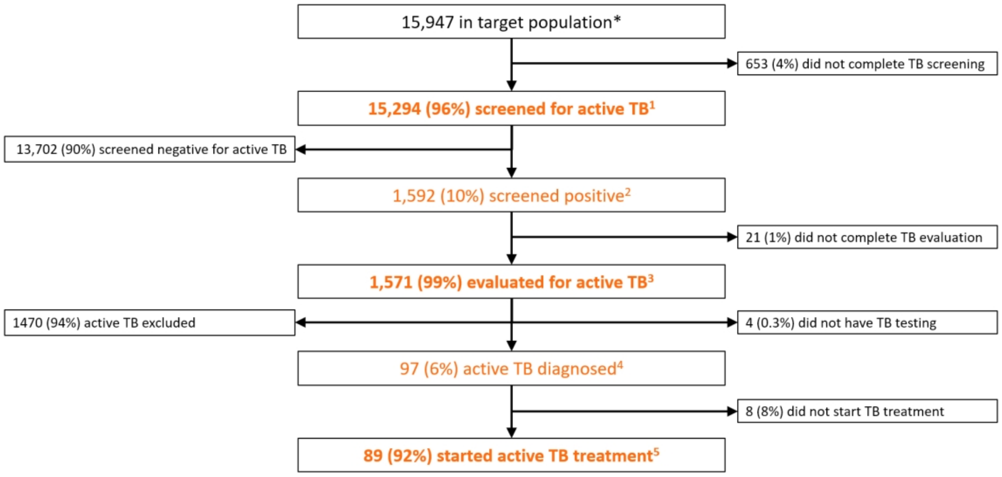

Figure 2. Flow diagram showing the Search Cascade as adapted from the Zero TB Indicator Framework for Mongolia.
Abbreviations: TB, tuberculosis.
Legend: Flow diagrams showing individuals entering and exiting at each step of the three cascades, either appropriately to the left side after having received guideline-adherent care or inappropriately to the right side after having received guideline-nonadherent care. Zero TB Indicators Treat indicators are shown in orange, with superscripts identifying indicator numbers as defined in Table 1. The indicators in bolded text describe the quality of care at a given step in the cascade, while the non-bolded indicators describe the diagnostic and/or epidemiological yield of that step in TB care.

839x415mm (57 x 57 DPI)

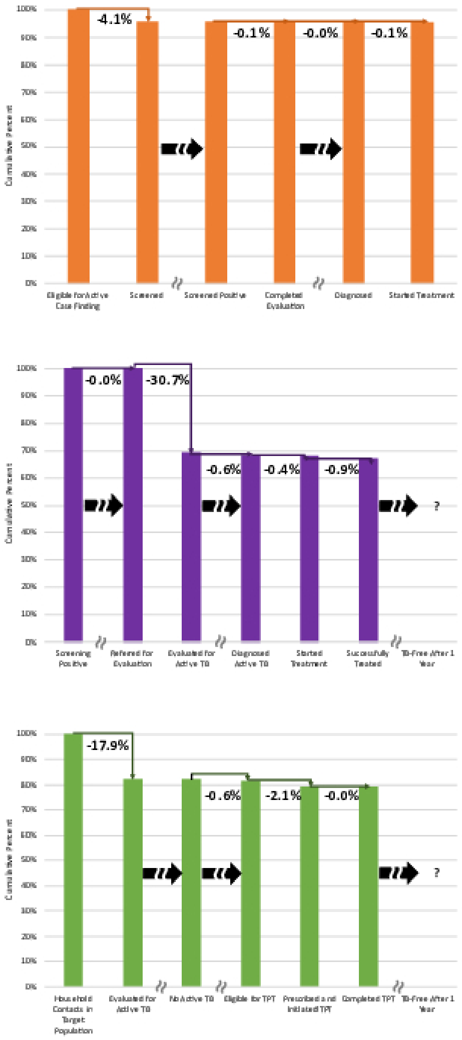

Figure 3. Cumulative proportions of participants receiving high-quality TB care, by delivery cascade. Panel A. Search Cascade (n=15,947); Panel B. Treat Cascade (n=8,518); Panel C. Prevent Cascade (n=2352). Abbreviations: TB, tuberculosis. Legend: Bar graphs showing the cumulative probability of completing the key processes with each of the three TB delivery cascades. The probabilities for each step, shown as bars, were calculated as the simple proportion of all individuals receiving guideline-recommended care at the end of that step and all previous steps, divided by the total number entering the cascade. Thin elbow connector lines show the percentage lost with each step, calculated as the simple proportion of individuals not receiving guideline-recommended care at that step divided by the total number entering the cascade. Block arrows with discontinuity lines shows steps where individuals exit the cascade having received all guideline-recommended care. The question mark indicates that follow-up data on outcomes at one year were not available as these are not routinely collected in Mongolia.

215x279mm (72 x 72 DPI)

For peer review only - http://bmjopen.bmj.com/site/about/guidelines.xhtml

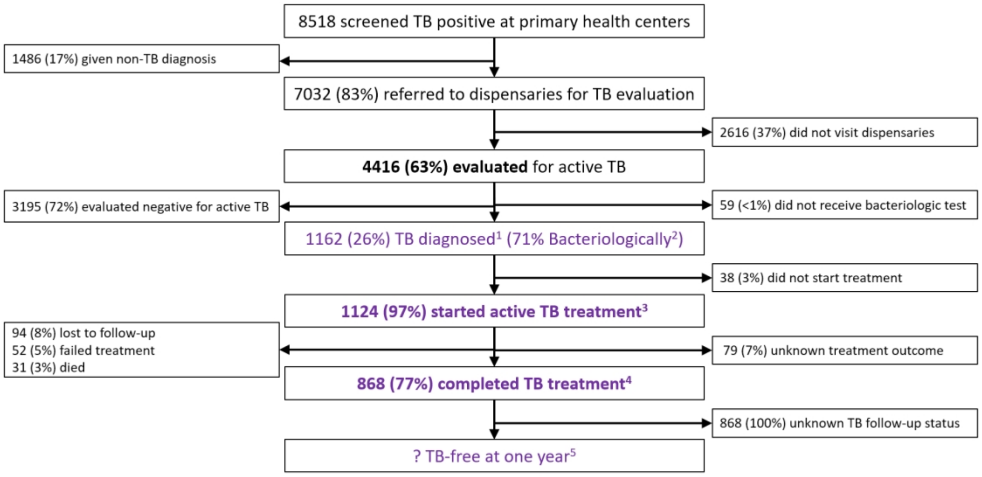

Figure 4. Flow diagram showing the Treat Cascade as adapted from the Zero TB Indicator Framework for Mongolia. Abbreviations: TB, tuberculosis. Legend: Flow diagrams showing individuals entering and exiting at each step of the three cascades, either appropriately to the left side after having received guideline-adherent care or inappropriately to the right side after having received guideline-nonadherent care. Zero TB Treat indicators are shown in purple, with superscripts identifying indicator numbers as defined in Table 1. The indicators in bolded text describe the quality of care at a given step in the cascade, while the non-bolded indicators describe the diagnostic and/or epidemiological yield of that step in TB care.

864x410mm (57 x 57 DPI)

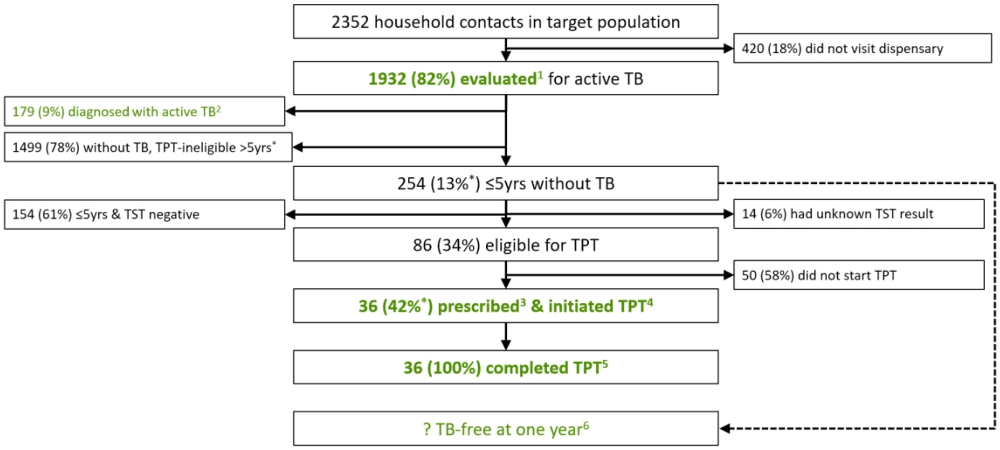

Figure 5. Flow diagram showing the Prevent Cascade, as adapted from the Zero TB Indicator Framework for Mongolia. Abbreviations: TB, tuberculosis. Legend: Flow diagrams showing individuals entering and exiting at each step of the three cascades, either appropriately to the left side after having received guideline-adherent care or inappropriately to the right side after having received guideline-nonadherent care. The adapted Zero TB Treat indicators are shown in green and numbered using superscripts as defined in Table 1. The indicators in bolded text describe the quality of care at a given step in the cascade, while the non-bolded indicators describe the diagnostic and/or epidemiological yield of that step in TB care. *Percentages derived from multivariate, logistic regression models using generalizing estimations and may differ from crude proportions. ?, unknown.

846x379mm (57 x 57 DPI)

SUPPLEMENTAL FIGURES AND TABLES

Table S0. Treat cascade indicators and cumulative probability of receiving high-quality TB diagnosis and treatment, stratified by study site.

Indicator	Bayanzurkh	Khan-Uul	Chingeltei	Mandal	p-value
Screened Positive	n=3,245	n=2,620	n=2,160	n=493	
Referred	2,867 (88%)	2,217 (85%)	1,455 (67%)	493 (100%)	<0.001
Evaluated	1,349 (47%)	1,372 (62%)	1,208 (83%)	487 (99%)	<0.001
Diagnosed[1]	595 (44%)	249 (18%)	276 (23%)	42 (9%)	<0.001
Started Treatment[3]	577 (97%)	237 (95%)	271 (98%)	39 (93%)	0.090
Completed Treatment[4]	416 (72%)	202 (85%)	216 (80%)	34 (87%)	<0.001
Cumulatively Diagnosed & Treated	29%	42%	44%	80%	<0.001

Legend: The Zero TB Treat indicators are numbered using superscripts as defined in Table 1. Shaded rows indicate steps whose yield is determined by the quality of care, while non-shaded rows indicate steps whose yield is determined by epidemiology and the sensitivity of screen and diagnostic tools.

Table S1. Predictors of Starting Active TB Treatment (Treatment Cascade)

Characteristic (n=1162)	aOR	95% CI	p-Value
Age (per decade)	0.72	0.66-0.79	<0.001
Male gender	0.73	0.62-0.86	<0.001

Abbreviations: aOR, adjusted odds ratio. 95% CI, 95% Confidence Intervals.

Table S2. Predictors of Completing Active TB Treatment (Treatment Cascade)

Characteristic (n=1124)	aOR	95% CI	p-Value
Age (per decade)	0.76	0.71-0.83	<0.001
Male gender	0.56	0.36-0.88	0.013

Abbreviations: aOR, adjusted odds ratio. 95% CI, 95% Confidence Intervals.

For peer review only

Table S3. Predictors of Completing TB Evaluation (Prevent Cascade)

Characteristic (n=2352)	aOR	95% CI	p-Value
Age of contact (per decade)	0.92	0.86-0.98	0.015
Male gender of contact	0.90	0.73-1.12	0.35
Immediate family member of index patient	0.92	0.71-1.17	0.48
Age of index patient (per decade)	1.01	0.93-1.10	0.81
Male gender of index patient	0.89	0.64-1.24	0.47

Abbreviations: aOR, adjusted odds ratio. 95% CI, 95% Confidence Intervals.

Table S4. Predictors of Being Prescribed and Initiating TPT (Prevent Cascade)

Characteristic (n=86)	aOR	95% CI	p-Value
Age of contact (per year)	0.76	0.55-1.06	0.11
Male gender of contact	0.78	0.22-2.82	0.71
Immediate family member of index patient	0.46	0.10-1.99	0.30
Age of index patient (per decade)	0.60	0.38-0.93	0.022
Male gender of index patient	0.97	0.37-2.57	0.95

Abbreviations: aOR, adjusted odds ratio. 95% CI, 95% Confidence Intervals.

For peer review only

Appendix S1.

Search Strategy

We search all articles in PubMed in all language published between January 1, 2000, and September 3, 2021, using the following strategy:

1. Search terms for tuberculosis

"tuberculosis"[MeSH] OR *Mycobacterium tuberculosis*[tiab] OR TB[tiab]

2. Search terms for cascade analysis

cascade[tiab] OR cascades[tiab] OR continuum[tiab] OR continua[tiab] or Process Assessment, Health Care[MeSH]

3. Search terms for low- and middle-income countries

(Deprived Countries[tw] OR Deprived Population[tw] OR Deprived Populations[tw] OR Developing Countries[tw] OR Developing Country[tw] OR Developing Economies[tw] OR Developing Economy[tw] OR Developing Nation[tw] OR Developing Nations[tw] OR Developing Population[tw] OR Developing Populations[tw] OR Developing World[tw] OR LAMI Countries[tw] OR LAMI Country[tw] OR Less Developed Countries[tw] OR Less Developed Country[tw] OR Less Developed Economies [tw] OR Less Developed Nation[tw] OR Less Developed Nations[tw] OR Less Developed World[tw] OR Lesser Developed Countries[tw] OR Lesser Developed Nations[tw] OR LMIC[tw] OR LMICS[tw] OR Low GDP[tw] OR Low GNP[tw] OR Low Gross Domestic[tw] OR Low Gross National[tw] OR Low Income Countries[tw] OR Low Income Country[tw] OR Low Income Economies [tw] OR Low Income Economy[tw] OR Low Income Nations[tw] OR Low Income Population[tw] OR Low Income Populations[tw] OR Lower GDP[tw] OR lower gross domestic[tw] OR Lower Income Countries[tw] OR Lower Income Country[tw] OR Lower Income Nations[tw] OR Lower Income Population[tw] OR Lower Income Populations[tw] OR Middle Income Countries[tw] OR Middle Income Country[tw] OR Middle Income Economies [tw] OR Middle Income Nation[tw] OR Middle Income Nations[tw] OR Middle Income Population[tw] OR Middle Income Populations[tw] OR Poor Countries[tw] OR Poor Country[tw] OR Poor Economies [tw] OR Poor Economy[tw] OR Poor Nation[tw] OR Poor Nations[tw] OR Poor Population[tw] OR Poor Populations[tw] OR poor world[tw] OR Poorer Countries[tw] OR Poorer Economies [tw] OR Poorer Economy[tw] OR Poorer Nations[tw] OR Poorer Population[tw] OR Poorer Populations[tw] OR Third World[tw] OR Transitional Countries[tw] OR Transitional Country[tw] OR Transitional Economies[tw] OR Transitional Economy[tw] OR Under Developed Countries[tw] OR Under Developed Country[tw] OR under developed nations[tw] OR Under Developed World[tw] OR Under Served Population[tw] OR Under Served Populations[tw] OR Underdeveloped Countries[tw] OR Underdeveloped Country[tw] OR underdeveloped economies[tw] OR underdeveloped nations[tw] OR underdeveloped population[tw] OR Underdeveloped World[tw] OR Underserved Countries[tw] OR Underserved Nations[tw] OR Underserved Population[tw] OR Underserved Populations[tw])

For peer review only

The 23 studies that we identified were:

1. Davis JL, Katamba A, Vasquez J, Crawford E, Sserwanga A, Kakeeto S, Kizito F, Dorsey G, den Boon S, Vittinghoff E, Huang L, Adatu F, Kamya MR, Hopewell PC, Cattamanchi A. Evaluating tuberculosis case detection via real-time monitoring of tuberculosis diagnostic services. Am J Respir Crit Care Med. 2011;184(3):362-7. PMID: 21471088; PMCID: 3175538.

2. Davis JL, Dowdy DW, den Boon S, Walter ND, Katamba A, Cattamanchi A. Test and treat: a new standard for smear-positive tuberculosis. J Acquir Immune Defic Syndr. 2012;61(1):e6-e8. PMID: 22918128; PMCID: 3427531.

3. Marquez C, Davis JL, Katamba A, Haguma P, Ochom E, Ayakaka I, Chamie G, Dorsey G, Kamya MR, Charlebois E, Havlir DV, Cattamanchi A. Assessing the quality of tuberculosis evaluation for children with prolonged cough presenting to routine community health care settings in rural Uganda. PLoS One. 2014;9(8):e105935. PMID: 25170875; PMCID: 4149493.

4. Sun AY, Denkinger CM, Dowdy DW. The impact of novel tests for tuberculosis depends on the diagnostic cascade. Eur Respir J. 2014;44(5):1366-9. PMID: 25186263; PMCID: PMC4254765.

5. Chaisson LH, Katamba A, Haguma P, Ochom E, Ayakaka I, Mugabe F, Miller C, Vittinghoff E, Davis JL, Handley MA, Cattamanchi A. Theory-informed interventions to improve the quality of tuberculosis evaluation at Ugandan health centers: a quasi-experimental study. PLoS ONE. 2015;10(7):e0132573; PMCID: 4501843.

6. Alsdurf H, Hill PC, Matteelli A, Getahun H, Menzies D. The cascade of care in diagnosis and treatment of latent tuberculosis infection: a systematic review and meta-analysis. Lancet Infect Dis. 2016;16(11):1269-78. PMID: 27522233.

7. Daftary A, Jha N, Pai M. Enhancing the role of pharmacists in the cascade of tuberculosis care. Journal of Epidemiology and Global Health. 2016;7(1):1.

8. Subbaraman R, Nathavitharana RR, Satyanarayana S, Pai M, Thomas BE, Chadha VK, Rade K, Swaminathan S, Mayer KH. The Tuberculosis Cascade of Care in India's Public Sector: A Systematic Review and Meta-analysis. PLOS Medicine. 2016;13(10):e1002149.

9. Armstrong-Hough M, Turimumahoro P, Meyer AJ, Ochom E, Babirye D, Ayakaka I, Mark D, Ggita J, Cattamanchi A, Dowdy D, Mugabe F, Fair E, Haberer JE, Katamba A, Davis JL. Drop-out from the tuberculosis contact investigation cascade in a routine public health setting in urban Uganda: A prospective, multi-center study. PLoS One. 2017;12(11):e0187145. PMID: 29108007; PMCID: PMC5673209.

10. Cazabon D, Alsdurf H, Satyanarayana S, Nathavitharana R, Subbaraman R, Daftary A, Pai M. Quality of tuberculosis care in high burden countries: the urgent need to address gaps in the care cascade. Int J Infect Dis. 2017;56:111-6. PMID: 27794468; PMCID: PMC5346036.

11. Hanrahan CF, Van Rie A. A proposed novel framework for monitoring and evaluation of the cascade of HIV-associated TB care at the health facility level. Journal of the International AIDS Society. 2017;20(1):21375.

12. Mwangwa F, Chamie G, Kwarisiima D, Ayieko J, Owaraganise A, Ruel TD, Plenty A, Tram KH, Clark TD, Cohen CR, Bukusi EA, Petersen M, Kamya MR, Charlebois ED, Havlir DV, Marquez C. Gaps in the Child Tuberculosis Care Cascade in 32 Rural Communities in Uganda and Kenya. J Clin Tuberc Other Mycobact Dis. 2017;9:24-9. PMID: 29291251; PMCID: PMC5743212.

13. Naidoo P, Theron G, Rangaka MX, Chihota VN, Vaughan L, Brey ZO, Pillay Y. The South African Tuberculosis Care Cascade: Estimated Losses and Methodological Challenges. J Infect Dis. 2017;216(suppl_7):S702-s13. PMID: 29117342; PMCID: PMC5853316.

14. Yuen C, Becerra M, Codlin A, Creswell J, Ditiu L, Keshavjee S, Khan A, Nicholson T, Page-Shipp L, Sahu S, Wilson M. A best-practice framework of program indicators for monitoring a comprehensive approach to the tuberculosis epidemic. Zero TB Initiative, 2017.

15. Shapiro AE, van Heerden A, Schaafsma TT, Hughes JP, Baeten JM, van Rooyen H, Tumwesigye E, Celum CL, Barnabas RV. Completion of the tuberculosis care cascade in a community-based HIV linkage-to-care study in South Africa and Uganda. J Int AIDS Soc. 2018;21(1). PMID: 29381257; PMCID: PMC5810338.

16. Agins BD, Ikeda DJ, Reid MJA, Goosby E, Pai M, Cattamanchi A. Improving the cascade of global tuberculosis care: moving from the "what" to the "how" of quality improvement. The Lancet Infectious Diseases. 2019;19(12):e437-e43.

17. Arsenault C, Roder-DeWan S, Kruk ME. Measuring and improving the quality of tuberculosis care: A framework and implications from the Lancet Global Health Commission. J Clin Tuberc Other Mycobact Dis. 2019;16:100112. PMID: 31497655; PMCID: PMC6716550.

18. Kim J, Keshavjee S, Atun R. Health systems performance in managing tuberculosis: analysis of tuberculosis care cascades among high-burden and non-high-burden countries. J Glob Health. 2019;9(1):010423. PMID: 31263546; PMCID: PMC6592589 www.icmje.org/coi_disclosure.pdf (available upon request from the corresponding author), and declare no conflicts of interest.

19. Subbaraman R, Nathavitharana RR, Mayer KH, Satyanarayana S, Chadha VK, Arinaminpathy N, Pai M. Constructing care cascades for active tuberculosis: A strategy for program monitoring and identifying gaps in quality of care. PLoS Med. 2019;16(2):e1002754. PMID: 30811385; PMCID: PMC6392267.

20. Der JB, Grint D, Narh CT, Bonsu F, Grant AD. Where are patients missed in the tuberculosis diagnostic cascade? A prospective cohort study in Ghana. PLoS One. 2020;15(3):e0230604. PMID: 32191768; PMCID: PMC7081980.

21. Diaz G, Victoria AM, Meyer AJ, Nino Y, Luna L, Ferro BE, Davis JL. Evaluating the Quality of Tuberculosis Contact Investigation in Cali, Colombia: A Retrospective Cohort Study. Am J Trop Med Hyg. 2021. PMID: 33617470; PMCID: PMC8045602.

22. van de Water B, Meyer T, Wilson M, Young C, Gaunt B, le Roux K. TB prevention cascade at a district hospital in rural Eastern Cape, South Africa. Public Health Action. 2021;11(2):97-100.

For peer review only

23. Yasobant S, Bhavsar P, Kalpana P, Memon F, Trivedi P, Saxena D. Contributing Factors in the Tuberculosis Care Cascade in India: A Systematic Literature Review. Risk Management and Healthcare Policy. 2021;Volume 14:3275-86.

Findings of the systematic review

We searched PubMed in all languages using a systematic search strategy to identify all articles published between January 1, 2000, and September 3, 2021 including compound search terms related to tuberculosis ("tuberculosis"[MeSH] OR *Mycobacterium tuberculosis*[tiab] OR TB[tiab]); cascades, continua of care, or process evaluations in health care (cascade[tiab] OR cascades[tiab] OR continuum[tiab] OR continua[tiab] or Process Assessment, Health Care[MeSH]); and low- and middle-income countries using a complex search strategy (Appendix S1). We screened titles for articles related to TB case-finding, treatment, and/or prevention services, and augmented our search by reviewing citations from retrieved articles to identify additional studies. We identified 22 articles and one monograph from the grey literature, all published between 2011 and 2021 and employing a wide variety of approaches to constructing TB care cascades. Sixteen articles focused on clinic-based TB case finding and treatment, two on TB contact investigation, three on TB preventive therapy, and three on case-finding, prevention, and treatment in combination. Two studies focused on children, while the remaining studies included adults only or adults and children. Eleven studies constructed cascades using primary patient data; four used parameters derived from systematic reviews; three studies used focused literature reviews to parameterize mathematical models; three mentioned the cascade of care as a concept in a review or editorial but did not construct their own cascades; and two proposed integrated frameworks for assessing the quality of case-finding, treatment, and prevention, including one focused on persons living with HIV. Two of the studies constructed cascades by integrating two of these approaches. Only two studies employed cascades as a quality improvement strategy, with both providing real-time performance feedback. Among the 11 studies that constructed cascades from patient data, only two did so using routine surveillance data, and both of these relied on aggregated

For peer review only

national data rather than on individual patient data. The remaining nine studies used subnational data collected by research staff using research databases. We did not identify any studies that collected individual-patient data from routine public health databases.

For peer review only

Appendix S2. Rationale for Zero TB Indicator targets provided in Table 1.

Indicator* (Proportion)	Target/ Source	Rationale
**SEARCH CASCADE**		
1. (Screening) Coverage	100%[1]	WHO guidelines state that all patients at risk for TB should be screened.
2. Positive TB symptom screen or chest radiography	25-50%[1]	Systematic reviews undertaken to inform the WHO guidelines examined the WHO 4-symptom screen in the general population and found a positivity rate for symptom screening of approximately 36%, with the 95% Confidence Interval (CI) ranging from 25 to 48%. Concurrent reviews of chest radiography found a lower positivity rate of approximately 11%, with the 95% CI ranging from 8 to 15%. Since symptom screening is used in concert with chest radiography, we have based the target proportion on symptom screening. Substantially higher or lower proportions may suggest the need for quality assurance procedures to ensure that screeners are asking the questions appropriately.
3. Diagnostic evaluation	100%[1]	WHO guidelines state that all patients who are symptom-positive should be evaluated for TB.
4. (Active) TB Diagnosis	10-20%[2]	The Stop TB Partnership sputum smear microscopy guidelines state that the typical smear positivity rate is 10-20%.† Higher or lower proportions suggest the need for quality assurance procedures to ensure that screening and testing are being carried out appropriately.
5. Linkage to TB Treatment	100%[3]	WHO guidelines state that all patients are diagnosed with active TB should be linked to TB treatment.
**TREAT CASCADE**		
1. (Active) TB Diagnosis	10-20%[2]	The Stop TB Partnership sputum smear microscopy guidelines state that the typical smear positivity rate is 10-20%.† Higher or lower proportions suggest the need for quality assurance procedures to ensure that screening and testing are being carried out appropriately.
2. Bacteriologic Confirmation	70-90%[4,5]	WHO-commissioned systematic reviews cited in the WHO guidelines suggest that microbiologic tests detect just 70-90% of TB cases relative to a reference standard that includes clinically diagnosed cases.
3. Linkage to TB Treatment	100%[3]	WHO guidelines state that all patients who are diagnosed with active TB should be linked to TB treatment.
4. Treatment Success	≥90%[6]	WHO guidelines state that all patients at risk for TB should be screened.
5. TB-free Survival	≥95%[7]	A high-quality systematic review suggests that ≥95% of patients with drug-susceptible TB can be cured with standard 6-month drug regimens.

Appendix S2 continued. Rationale for Zero TB Indicator targets provided in Table 1.

**PREVENT CASCADE**		
TB Screening	100%[8]	WHO guidelines state that all close contacts of TB patients should be screened for active TB.
6. TB Evaluation	≥90%[6]	The END TB Strategy has set a target TB evaluation rate of ≥90% of symptomatic close contacts.
7. (Active) TB Diagnosis	10-20%[2]	Stop TB Partnership sputum microscopy guidelines state that the typical positivity rate is 10-20%.[†] Higher or lower proportions suggest the need for quality assurance procedures to ensure that screening and testing are being carried out appropriately.
TPT Eligibility	95%[9]	
8. (TPT) Prescription	≥90%[6]	The END TB Strategy has set a target prescription rate for TB preventive therapy of ≥90% of eligible individuals.
9. (TPT) Uptake	100%[6]	The END TB Strategy has set a target uptake rate for TB preventive therapy of ≥90% of eligible individuals
10. (TPT) Completion	≥80%[9]	WHO guidelines state that TPT regimen is considered complete when ≥80% doses have been completed.
11. TB-free survival	100%[9]	Systematic reviews cited within WHO guidelines had a very low TB incidence among those completing TB preventive therapy.

Abbreviations: TB, tuberculosis.
Legend: [*]Indicator numbers as proposed by the Zero TB Indicator Framework[10]. [†]The target proportion might be adjusted if a different diagnostic testing modality is in use, or based on robust empirical data.

For peer review only

Appendix S3. Derivation of the variance and confidence interval for estimated cumulative probability of being diagnosed & treated

Let p_1 be the probability of being referred among subjects who were screened positive, p_2 be the probability of being evaluated among subjects who were referred, p_3 be the probability of starting treatment among subjects who were diagnosed TB positive, and p_4 be the probability of complete treatment among subjects who started treatment. Then the cumulative probability of being properly diagnosed and treated for TB given a positive TB diagnosis $p = p_1 p_2\, p_3 p_4$.

Denote n the number of subjects who were screened positive, n_e the number of subjects who were evaluated, n_d the number of subjects who were diagnosed TB positive, and n_c the number of subjects who completed the treatment. The estimated p is

$$\hat{p} = \frac{n_e}{n}\frac{n_c}{n_d},$$

where $\frac{n_e}{n}$ is an estimator of $p_1 p_2$ and $\frac{n_c}{n_d}$ is an estimator of $p_3 p_4$.

We derive the variance of $\log(\hat{p}) = \log\left(\frac{n_e}{n}\right) + \log\left(\frac{n_c}{n_d}\right)$. Suppose the two probabilities $\frac{n_e}{n}$ and $\frac{n_c}{n_d}$ are independent. This is reasonable since the evaluation process and completion of treatment are different.

$$\widehat{Var}[\log(\hat{p})] = \widehat{Var}\left[\log\left(\frac{n_e}{n}\right)\right] + \widehat{Var}\left[\log\left(\frac{n_c}{n_d}\right)\right] = \left(\frac{n_e}{n}\right)^{-2}\widehat{Var}\left(\frac{n_e}{n}\right) + \left(\frac{n_c}{n_d}\right)^{-2}\widehat{Var}\left(\frac{n_c}{n_d}\right)$$
$$= \frac{n - n_e}{n n_e} + \frac{n_d - n_c}{n_d n_c}.$$

The first equality is due to independence of probabilities, the second is due to the delta method, and the last one is simple algebra by substituting the variance of probabilities. The variance of $\hat{p}$ is then by the delta method again,

$$\widehat{Var}[\hat{p}] = \hat{p}^2 * \widehat{Var}[\log(\hat{p})],$$

and the 95% CI is

$$\left(\hat{p} * \exp\left(-1.96 * \widehat{Var}[\log(\hat{p})]\right),\quad \hat{p} * \exp\left(1.96 * \widehat{Var}[\log(\hat{p})]\right)\right).$$

For peer review only

SUPPLEMENTAL REFERENCES

1. World Health Organization. WHO operational handbook on tuberculosis. Module 2: Screening. Systematic screening for tuberculosis disease. Geneva: World Health Organization; 2021.
2. Stop TB Partnership. Laboratory diagnosis of tuberculosis by sputum microscopy. In: Global Laboratory Initiative, editor. Adelaide, Australia: SA Pathology; 2013.
3. World Health Organization. WHO consolidated guidelines on tuberculosis: module 4: treatment: drug-resistant tuberculosis treatment: World Health Organization; 2020.
4. World Health Organization. Policy statement: The use of liquid medium for culture and DST in low- and middle-income country settings. Geneva: World Health Organization; 2007.
5. World Health Organization. WHO Meeting Report of a Technical Expert Consultation: Non-inferiority analysis of Xpert MTB/RIF Ultra compared to Xpert MTB/RIF Geneva: World Health Organization; 2017.
6. World Health Organization. The End TB Strategy. Geneva: World Health Organization; 2015.
7. Imperial MZ, Nahid P, Phillips PPJ, et al. A patient-level pooled analysis of treatment-shortening regimens for drug-susceptible pulmonary tuberculosis. *Nat Med* 2018; **24**(11): 1708-15.
8. World Health Organization. Recommendations for investigating contacts of persons with infectious tuberculosis in low- and middle-income countries. Geneva: World Health Organization; 2012.
9. World Health Organization. Latent tuberculosis infection: Updated and consolidated guidelines for programmatic management. Geneva: World Health Organization; 2018.
10. Yuen C, Becerra M, Codlin A, et al. A best-practice framework of program indicators for monitoring a comprehensive approach to the tuberculosis epidemic: Zero TB Initiative, 2017.

For peer review only
